# Reversible Substrate-Specific Photocontrol of the Chemotherapeutic Asparaginase(-Glutaminase) from *Escherichia coli*

**DOI:** 10.1021/acscatal.5c01608

**Published:** 2025-05-06

**Authors:** Mona Wieland, Jonnely Luizaga, Cristina Duran, Barbara Germscheid, Johanna Rein, Astrid Bruckmann, Caroline Hiefinger, Sílvia Osuna, Andrea Hupfeld

**Affiliations:** † Institute of Biophysics and Physical Biochemistry and Regensburg Center for Biochemistry, University of Regensburg, Universitätsstraße 31, D-93053 Regensburg, Germany; ‡ Institut de Química Computacional i Catàlisi and Departament de Química, Universitat de Girona, c/Maria Aurèlia Capmany 69, 17003 Girona, Spain; § Institute of Biochemistry, Genetics and Microbiology, University of Regensburg, Universitatsstrasse 31, D-93053 Regensburg, Germany; ∥ ICREA, Pg. Lluís Companys 23, 08010 Barcelona, Spain

**Keywords:** l-asparaginase, photocontrol, photoswitches, protein engineering, unnatural amino acids, molecular dynamics simulations, conformational landscapes

## Abstract

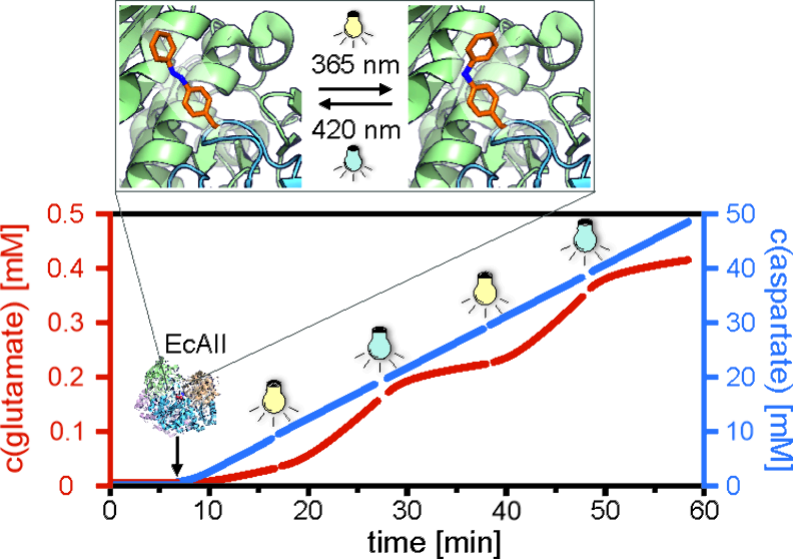

Photoswitchable unnatural amino acids are valuable engineering tools in biotechnology, particularly for the reversible control of enzymes with light. Here, we explore some basic principles of this protein engineering technique to simplify its approach and increase its success rate. To this end, we have selected *Escherichia coli* type II asparaginase (EcAII), which is a prominent chemotherapeutic enzyme that is limited by detrimental side effects associated with its promiscuous glutaminase activity. Incorporation of phenylalanine-4′-azobenzene (AzoF) combined with extensive biophysical characterizations identified two light-sensitive variants, in which glutamine hydrolysis could be reversibly (de)activated up to 9-fold, whereas asparaginase hydrolysis was only marginally light-responsive. Computationally determined conformational landscapes elucidated this substrate-specificity of photocontrol defining a clear engineering principle: An exchange between less and more productive states at the active site helps AzoF to reshape the conformational landscape and makes enzymes more susceptible toward photocontrol. Moreover, our findings mark EcAII-AzoF variants as potential chemotherapeutic precursors.

## Introduction

The spatiotemporal control of enzymes is of growing interest for diverse research fields ranging from chemical biology to clinical and industrial biotechnology.^1^ Allosteric control and light induced regulation with photoreceptors are two natural regulation mechanisms of enzymes that take advantage of their high flexibility. Particularly the latter has many benefits as tool in experimental sciences because light is an external stimulus that is cost-effective, environmentally friendly and easy to apply. Photoreceptors have therefore found a broad interest for the spatiotemporal control of biological processes in the field of optogenetics.^2−4^ Likewise, the technological progress to reprogram the genetic code by amber suppression has facilitated the incorporation of synthetic photoswitches into proteins in the form of unnatural amino acids (UAAs),^5,6^ a strategy that holds complementary advantages for the spatiotemporal control of enzymes compared to optogenetics. We have dubbed this method photoxenoprotein engineering^1^ and introduced the term “photoxenase” to distinguish enzymes that have been rendered light-sensitive in this way from noncatalytic proteins (by using the common suffix “-ase” that stands for “enzyme”).

**Figure 1:**
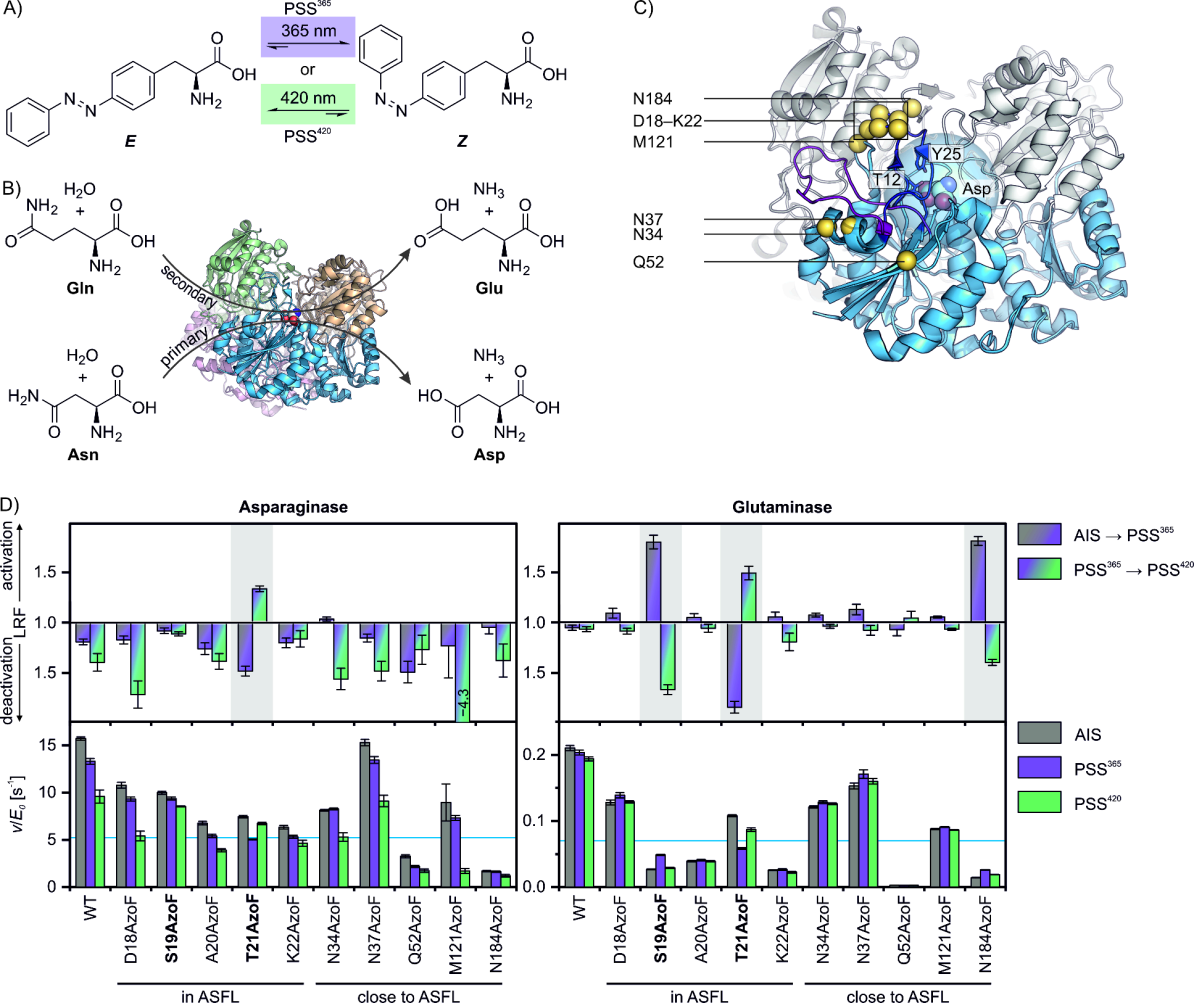
Engineering of photocontrol in EcAII. (A) Irradiation with different wavelengths establishes either an *E*- or *Z*-enriched PSS in AzoF. (B) Primary and secondary reaction of the homotetrameric EcAII. Asn: asparagine; Asp: aspartate; Gln: glutamine; Glu: glutamate. (C) Selected positions (yellow spheres) for incorporation of AzoF that are in or close to the ASFL (open: PDB-ID 7p9c, magenta; closed: PDB-ID 3eca, deep purple) and that are at least 10 Å distant to the Cγ atom of aspartate (highlighted by a 10 Å light-blue sphere around Cγ) as a marker for the active site. Note that residue N184 is located in chain D (gray), whereas the others are located in chain C (blue). (D) Asparaginase and glutaminase activities of WT-EcAII and EcAII-AzoF variants. Each bottom subpanel shows the fitted catalytic rate normalized to the enzyme concentration (*v/E*_0_) ± standard error of fit (SE). Blue lines mark a third of the as-isolated wild-type activity. Each top subpanel shows the fitted LRF value ± standard error of fit (SE) as a measure of photocontrol efficiency. For more details see the raw data plots in Figures S4–S5 and Tables S1–S2.

Hitherto, two photoswitches have been designed and incorporated as UAA, azobenzene^7−11^ and arylazopyrazole.^12,13^ Phenylalanine-4′-azobenzene (AzoF),^7^ which can be readily synthesized and incorporated,^7,14,15^ exists in two states, an elongated *E* and a bent *Z* isomer that is thermally less stable (Figure 1A). Exposure to light establishes a photoinduced equilibrium between *E* and *Z* called photostationary state (PSS) that depends on the wavelength of irradiation. Thus, ultraviolet (UV) light of, e.g., 365 nm, achieves a *Z*-enriched PSS^365^ and visible (Vis) light of, e.g., 420 nm, an *E*-enriched PSS^420^. Encouragingly, this wavelength steered switch between PSSs allows for the desired reversible regulation of enzyme activity. However, the engineering of switchable photoxenases with AzoF remains a difficult challenge, especially compared to the engineering with photocaged UAAs,^1,16^ which facilitate an irreversible photoactivation of enzymes.

Remarkably, we were able to efficiently photocontrol the allosteric bienzyme complex imidazole glycerol phosphate synthase (ImGPS) in recent studies using photoxenoprotein engineering.^17,18^ Incorporation of AzoF at two strategic positions distant from the active site facilitated the reversible regulation of enzyme allostery and activity with a light regulation factor (LRF) of up to 10. One of the incorporation sites is positioned in a flexible loop close to the allosteric ligand binding site, and the other is crucial for the allosterically important hinge motion at the enzyme interface. Simultaneously, our accelerated molecular dynamics (MD) simulations have shown a heterogeneous conformational landscape in ImGPS that shifts upon ligand binding or *E*→*Z* isomerization of AzoF and that is crucial for the catalytic activity.^18,19^ These findings led to the hypothesis that a pre-existing equilibrium between conformations of low and high productivity in the enzyme target might benefit the successful engineering of photoxenases with AzoF.

To test this hypothesis, we looked for a model system that exhibits such conformational diversity and is of significance for an application of topical interest. The nonallosteric homotetrameric type II asparaginase from *Escherichia coli* (EcAII), which catalyzes the hydrolysis of asparagine to aspartate and in a promiscuous secondary reaction the hydrolysis of glutamine to glutamate (Figure 1B), meets these criteria. Substrate-induced closure of its active site flexible loop (ASFL) shifts EcAII from an inactive to an active state (Figure S1A). Its catalytic mechanism with asparagine follows an induced fit with a rate-limiting chemical step,^20^ which includes a threonine (T12) for the nucleophilic attack of the substrate, four supporting catalytic residues (Y25, T89, D90 and K162) and two conserved water molecules (Figure S1B).^21,22^ Notably, EcAII has been used as chemotherapeutic enzyme with the brand names Elspar and Oncaspar since the late 1970s. Its mode of action leads to the depletion of asparagine and glutamine in the extracellular environment of tumor cells, which strongly depend on the uptake of both amino acids due to a rewiring of the cell, and hence to their selective cell death.^23^ While it is approved for the treatment of acute lymphoblastic leukemia (ALL),^24,25^ it also targets solid tumors such as pancreatic adenocarcinoma, for which a phase III trial is ongoing.^26^ Remarkably, its promiscuous glutaminase activity, which is necessary for the deleterious effect on tumor cells,^25,27^ evokes toxic side effects when applied systemically.^28^ This makes it a particularly interesting enzyme target for photocontrol, by which it could be locally activated at the site of the tumor.

In the present work, we tested our assumption that an exchange between conformations with low or high catalytic productivity and therefore high conformational heterogeneity might ease the engineering of a switchable photoxenase. For this, we introduced AzoF into the ASFL resulting in the reversible light-dependence of glutamine hydrolysis but not asparagine hydrolysis in two variants. We substantiated the photocontrol efficiency as well as its reversibility in an extensive biophysical characterization and finally correlated the low and high photocontrol efficiencies of the asparaginase and glutaminase reaction with alterations in ASFL conformation as determined through computational conformational landscape reconstruction.

## Results

### Identification of EcAII Based Photoxenases

We determined four criteria to select positions of AzoF incorporation in a rational design approach (Figure 1C). First, AzoF should be inserted in the ASFL or close to its open or closed state to influence its conformation. Second, the positions should at least be ∼10 Å distant to the active site. This approach is similar to the engineering of our previous ImGPS- photoxenases^17,18^ and avoids direct interference of AzoF with catalysis. Third, we excluded loop motion mediating glycine positions in the ASFL. Finally, AzoF should only minimally clash with the van der Waals radii of neighboring residues, which we tested by in silico incorporation using a previously calculated rotamer library of AzoF^18^ and the SwissSideChain^29^ tool of PyMOL.^30^ Applying these criteria, we selected nine positions. D18, S19, T21 and K22 are located between the two catalytic residues T12 and Y25 on the ASFL. N34, N37, Q52, M121 and N184 are within 6 Å to the ASFL. Notably, Q52 is the only highly conserved residue (Figure S2). We produced and purified wild-type (WT-)EcAII and the nine EcAII-AzoF variants using a previously designed orthogonal aaRS/tRNA pair that incorporates AzoF with high efficiency (Figure S3).^7,18^ For the sake of brevity, we refer to EcAII enzymes that contain AzoF at position “pos” with the term EcAII-posAzoF, e.g., incorporation of AzoF at position D18 obtains the variant EcAII-D18AzoF. To screen the EcAII-AzoF variants, we initially established coupled enzymatic assays for continuous, spectrophotometric monitoring of the asparagine and glutamine reactions (Figure S4A, Figure S5A). The rate of asparagine and glutamine turnover linearly increased with growing enzyme concentrations for all our variants validating that the assays reliably reflect the catalytic activity of EcAII (Figure S4B, Figure S5B). We then deduced the *v*/*E*_0_ values for each variant in its as-isolated state (AIS), which is thermally equilibrated to ∼100% *E*, in its *Z*-enriched PSS^365^ and in its *E*-enriched PSS^420^ (Figure 1D bottom subpanels; Table S1 and Table S2), and determined two LRFs comparing the difference in activity between either the AIS and PSS^365^ or PSS^365^ and PSS^420^, where an LRF of one implies no change in activity upon irradiation (Figure 1D, top subpanels).

Incorporation of AzoF only led to an overall asparaginase activity loss to less than a third of the as-isolated wild-type activity in two positions including the highly conserved Q52. Notably, WT-EcAII activity decreased to ∼85% after 365 nm irradiation (LRF ∼ 1.2) and to ∼ 61% after 420 nm irradiation (LRF ∼ 1.4) compared to its AIS. Most EcAII-AzoF variants shared this general loss in activity upon irradiation except EcAII-T21AzoF, which showed a very weak reversible photocontrol of asparaginase hydrolysis with a ∼1.5-fold decrease in PSS^365^ and a ∼1.3-fold increase in PSS^420^.

Next, we analyzed whether promiscuous glutamine hydrolysis might be more susceptible toward photocontrol in EcAII. The derived *v/E*_*0*_ values of the EcAII-AzoF variants demonstrated that glutamine turnover was more strongly hampered compared to WT-EcAII than asparagine turnover with four variants exhibiting less than a third of the as-isolated wild-type activity. Interestingly, glutaminase activity was generally less affected by irradiation with a decrease to ∼97% after 365 nm (LRF ∼ 1.0) and to ∼92% after 420 nm exposure (LRF ∼ 1.0) in WT-EcAII. The three variants EcAII-S19AzoF, EcAII-T21AzoF and EcAII-N184AzoF showed a difference in activity upon 365 nm irradiation (LRF > 1.5) that was largely reversible by 420 nm irradiation. Notably, all of these positions are evolutionarily preferred with major occurrences in a multiple sequence alignment of serine in position 19, threonine in position 21 and asparagine or aspartate in position 184 (Figure S2). Moreover, they cluster together structurally with positions 18, 22, and 121 in the closed ASFL conformation (Figure 1C). However, EcAII-D18AzoF, EcAII-K22AzoF and EcAII-M121AzoF exhibited only weak reversible activity changes (LRFs < 1.2). Surprisingly, while all other EcAII variants with AzoF in this cluster increased their activity upon 365 nm irradiation and decreased their activity upon 420 nm irradiation, EcAII-T21AzoF showed an inverse effect. These observations prompted us to incorporate AzoF in position A20 that is also located in this cluster connecting S19 and T21. We initially excluded this residue position because AzoF showed significant clashes with the van der Waals radii of neighboring residues after in silico incorporation. In fact, consistent with these clashes EcAII-A20AzoF exhibited reduced asparagine turnover of ∼34% compared to as-isolated WT-EcAII and even lower glutamine turnover of ∼19%. Furthermore, both reactions remained largely unchanged upon irradiation (Figure 1D).

In conclusion, we identified three EcAII based photoxenases, i.e. EcAII-S19AzoF, EcAII-T21AzoF and EcAII-N184AzoF. Owing to its drastically reduced activity, we decided to exclude EcAII-N184AzoF from our subsequent studies.

### Kinetic Behavior of EcAII-S19AzoF and EcAII-T21AzoF

In the next step, we biophysically and kinetically examined the two photoxenases in comparison to WT-EcAII to confirm the photocontrol efficiencies for both reactions. For this, we first verified the identity of each protein by tryptic digest coupled to mass spectrometry (MS) (Figure S6). Circular dichroism spectroscopy then demonstrated that both photoxenases are properly folded and as stable as WT-EcAII with denaturation midpoints of ∼60 °C (Figure S7). Moreover, analytical size-exclusion chromatography in combination with static light scattering detection corroborated that WT-EcAII, EcAII-S19AzoF and EcAII-T21AzoF exist as homotetramers (Figure S8). Finally, we determined the *E*:*Z* distributions at the PSS (PSD) using UV/vis analysis of both photoxenases (Figure S9–11). The PSD^365^ exhibited 29*E*:71*Z* and 23*E*:77*Z*, and the PSD^420^ 94*E*:6*Z* and 92*E*:8*Z* for EcAII-S19AzoF and EcAII-T21AzoF, respectively. Furthermore, the establishment of both photoinduced equilibria was quite fast with half-times of *t*_*1/2*_ < 0.2 s for PSS^365^ and *t*_*1/2*_ ∼ 0.8 s for PSS^420^. Hence, the photoinduced isomer switch of AzoF proceeded with comparable efficiencies in both photoxenases.

**Figure 2:**
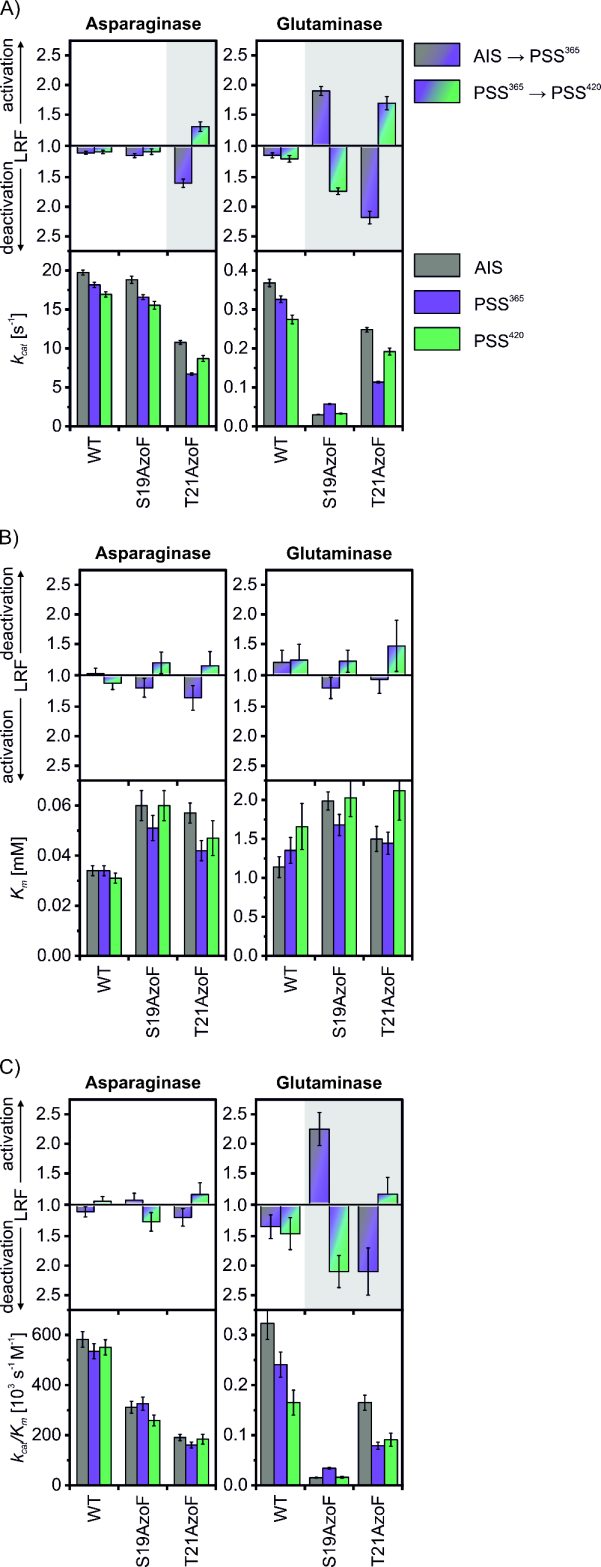
Comparison of steady state kinetic values *k*_*cat*_ (A), *K*_*m*_ (B) *k*_*cat*_/*K*_*m*_ (C) with their respective LRFs of WT-EcAII, EcAII-S19AzoF and EcAII-T21AzoF. *Statistics*: Each subpanel shows the fitted values ± SE of three technical replicates. For more details see the raw data plots in Figures S11 and Tables S3–S4.

For our kinetic analysis, we initially determined the Michaelis–Menten plots of WT-EcAII, EcAII-S19AzoF and EcAII-T21AzoF (Figure S12). For WT-EcAII, the asparaginase values of *k*_*cat*_ ∼ 20 s^–1^, *K*_*m*_ ∼ 0.03 mM and *k*_*cat*_/*K*_*m*_ ∼ 582 × 10^3^ s^–1^ M^–1^ (Figure 2, Table S3) coincided with previous reports (*k*_*cat*_: ∼24–60 s^–1^, *K*_*m*_: 0.01–0.1 mM, *k*_*cat*_/*K*_*m*_: 300–1975 × 10^3^ s^–1^ M^–1^).^20,31,32^ In comparison, as-isolated EcAII-S19AzoF exhibited a similar *k*_*cat*_, a 2-fold increased *K*_*m*_, and a 2-fold reduced *k*_*cat*_/*K*_*m*_. Likewise, as-isolated EcAII-T21AzoF demonstrated a 2-fold decreased *k*_*cat*_, a 2-fold increased *K*_*m*_, and a 3-fold reduced *k*_*cat*_/*K*_*m*_. Irradiation prior to the reaction led to a nonreversible reduction of the asparaginase *k*_*cat*_ in WT-EcAII and EcAII-S19AzoF to 88–92% (365 nm) and 82–86% (420 nm) compared to the AIS. In contrast, EcAII-T21AzoF reversibly decreased its *k*_*cat*_ value upon 365 and 420 nm irradiation (LRF ∼1.6 and ∼1.3). Both results confirm the findings of the initial screening. Interestingly, the effect of EcAII-T21AzoF was less pronounced for the *K*_*m*_ and the *k*_*cat*_/*K*_*m*_ values.

The glutaminase values for WT-EcAII of *k*_*cat*_ ∼ 0.37 s^–1^, *K*_*m*_ ∼ 1.1 mM and *k*_*cat*_/*K*_*m*_ ∼ 0.32 × 10^3^ s^–1^ M^–1^ (Figure 2, Table S4) were again in good agreement with previously reported values (*k*_*cat*_: 0.33–0.51 s^–1^, *K*_*m*_: 3.5–4.0 mM, *k*_*cat*_/*K*_*m*_: 0.09–0.13 × 10^3^ s^–1^ M^–1^).^31,32^ Remarkably, as-isolated EcAII-S19AzoF obtained a ∼12-fold reduced *k*_*cat*_, a 2-fold increased *K*_*m*_ and a ∼21-fold reduced *k*_*cat*_/*K*_*m*_ compared to WT-EcAII. In contrast, as-isolated EcAII-T21AzoF was comparably active to WT-EcAII with a similar *k*_*cat*_ and *K*_*m*_, and an only 2-fold reduced *k*_*cat*_/*K*_*m*_. Irradiation of WT-EcAII again caused a nonreversible reduction of the glutaminase *k*_*cat*_ to overall 74%. While EcAII-S19AzoF reversibly increased its *k*_*cat*_ value (LRF ∼1.9 and ∼1.7), EcAII-T21AzoF reversibly decreased its *k*_*cat*_ value (LRF ∼2.2 and ∼1.7), again corroborating the results of our initial screening. Comparable to the asparaginase reaction, the photocontrol effect was less pronounced for the *K*_*m*_ value for both photoxenases, however, the *k*_*cat*_/*K*_*m*_ values showed a partly reversible regulation upon irradiation (EcAII-S19AzoF: LRF ∼2.2 and ∼2.1; EcAII-T21AzoF: LRF ∼2.1 and ∼1.1).

So far, we irradiated the enzymes in their apo conformation with an open ASFL prior to the measurement. Since substrate binding induces the closure of the ASFL to generate the holo conformation, we wondered whether irradiation of apo and holo EcAII might lead to different photoinduced conformational states resulting in diverging photocontrol efficiencies in the two photoxenases. To this end, we irradiated EcAII either in its apo AIS before addition to the reaction or in its holo AIS during the reaction with substrate concentrations in saturation since the *k*_*cat*_ showed the largest effects for both reactions. To prevent the photodamage of auxiliary enzymes through irradiation of the coupled assay and to minimize the loss in activity of WT-EcAII we optimized the length of irradiation for this approach (Extended Text S1; Figure S13). While EcAII-S19AzoF demonstrated the same low photocontrol efficiency of asparagine hydrolysis (LRF ∼ 1.1) in both conformational states, EcAII-T21AzoF appeared to be less prone to photocontrol in the holo state (LRF ∼ 1.1) than in the apo state (LRF ∼ 1.7). Surprisingly, both photoxenases exhibited a higher photocontrol efficiency of glutamine hydrolysis in the holo state (LRF ∼ 3.9 and ∼ 3.2) than in the apo state (LRF ∼ 2.3 and ∼ 2.4). Importantly, the irradiated apo state could be converted to the irradiated holo state with similar activity values. These results (details see Extended Text S2; Figures S14–S16) implied that the photocontrol efficiency depends on the conformational state, in which EcAII is irradiated.

In summary, we showed that the asparaginase activities of both photoxenases were largely retained with steady state constants affected up to 3-fold. In contrast, while the glutaminase activity of EcAII-T21AzoF was similar to WT-EcAII, EcAII-S19AzoF was hampered up to 21-fold. Moreover, we substantiated that both photoxenases are able to control the glutaminase reaction, for which the photocontrol efficiencies are higher in the presence of substrate and the photocontrol effect is primarily caused by a change in *k*_*cat*_. Intriguingly, we found that the asparaginase reaction appears to be only marginally affected in holo EcAII-S19AzoF and holo EcAII-T21AzoF.

### Validation of Substrate-Specific Photocontrol in EcAII-S19AzoF and EcAII-T21AzoF

**Figure 3:**
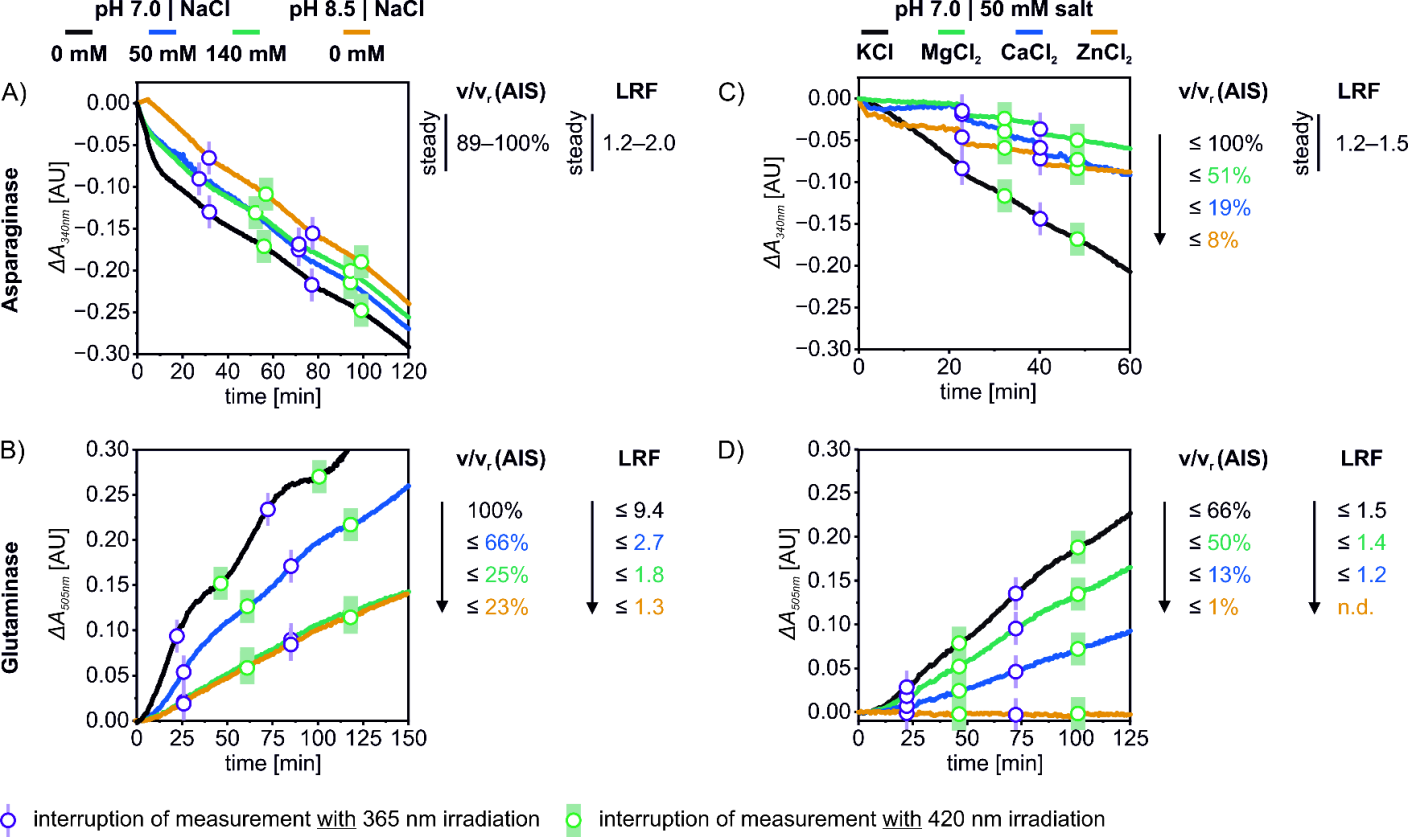
Exemplary progress curves supplemented with a summary of the AIS activity and the LRF for EcAII-T21AzoF presenting the effects of various reaction conditions on the asparaginase (A, C) and glutaminase (B, D) reactions. A, B) Comparison of pH 7.0 and pH 8.5 with 0 mM NaCl, and 0 mM, 50 mM and 140 mM NaCl at pH 7.0. C, D) Comparison of different types of salt (50 mM) at pH 7.0. Note: v/v_r_ (AIS) delineates the ratio of each AIS activity (v) compared to the reference AIS activity in pH 7.0 and 0 mM salt (v_r_); the v/v_r_ and LRF values correspond to the upper values reached throughout all replicates. Photocontrol efficiencies were determined via irradiation of holo EcAII during the turnover measurement. For details see Figure S17–20 and Figure S23.

Our previous studies on photoxenases indicated that the LRF is strongly dependent on factors such as pH and salt.^18,33^ The negligible photocontrol of asparagine hydrolysis therefore prompted us to investigate whether the asparaginase and glutaminase reactions might require different conditions for an optimal photocontrol efficiency. To this end, we repeatedly irradiated holo EcAII with 365 or 420 nm during an interruption of the reaction measurement as exemplarily shown for EcAII-T21AzoF in Figure 3 (cf. Extended Text S1). We summarized the effects of each reaction condition in secondary plots providing the mean LRFs obtained after irradiation with 365 or 420 nm, respectively, and the activity of the AIS as control (Figures S17–S20). Notably, WT-EcAII showed LRFs of only 1.0–1.2 throughout all reaction screens.

We first repeated our previous measurements comprising Tris/HCl pH 7.0 without the addition of salt (Figure S17–S20), which obtained LRFs ≤ 1.3 (EcAII-S19AzoF) and ≤ 1.7 (EcAII-T21AzoF) for the asparaginase reaction and ≤ 3.3 (EcAII-S19AzoF) and ≤ 9.4 (EcAII-T21AzoF) for the glutaminase reaction. We then increased the pH stepwise to pH 8.5 and tested different salt concentrations using initially sodium chloride. An extended activity screen in all pH and c(NaCl) combinations (Figure S17–S20) demonstrated that the asparaginase activity and LRF is steady over various reaction conditions, whereas the glutaminase activity and LRF is adversely affected by increasing pH and high salt concentrations (Figure 3A,B). To examine whether this difference in photocontrol efficiency might result from a change in the PSDs, we recorded UV/vis spectra in high salt concentrations (Figure S21). The determined PSDs and half-times of PSS formation were similar to the ones obtained without salt (Figure S22) indicating that the decreased photocontrol efficiency originates from an interference of the salt with the enzyme but not with the photoswitch.

While addition of increasing sodium chloride concentrations could not improve the photocontrol efficiency of asparagine hydrolysis, we wondered whether other salts might be more effective. To this end, we tested different salts with various ionic strengths (Figure 3C,D). Interestingly, the asparaginase activities with sodium and potassium salts were comparable (50–100%) to the reaction condition without salt but dropped to 19–51% with magnesium or calcium salts and even to <8% with zinc salts (Figure S23A,B). The photocontrol efficiencies remained low (LRF < 1.5) throughout all measurements. The glutaminase reaction followed a similar trend with slightly decreased activities in the presence of sodium and potassium salts (38–66%), more strongly reduced activities with magnesium and calcium salts (13–50%), and almost no measurable activities with zinc salts (<1%) compared to the reaction without salt (Figure S23C,D). Moreover, while the photocontrol efficiencies of EcAII-S19AzoF remained consistent (LRF ∼ 2), the photocontrol was hampered for EcAII-T21AzoF [LRF(Na^+^) 2–3, LRF(K^+^, Mg^2+^, Ca^2+^) 1.2–2] compared to the reaction without salt (LRF ≤ 8.6). The LRFs in the presence of zinc salts could not be determined for either photoxenase due to the extremely low activities. Notably, the enzyme activities as well as the LRFs showed differences when using the same ionic strength of 150 mM, i.e. with Na_2_SO_4_, K_2_SO_4_, MgCl_2_, CaCl_2_ and ZnCl_2_. Conclusively, the inhibition of activity and photocontrol efficiency of glutamine hydrolysis appears to depend solely on the type of salt, particularly the type of cation.

In a last attempt to find reaction conditions that promote photocontrol of asparagine hydrolysis, we tested various buffer systems. By this, the asparaginase activities as well as the LRFs were largely maintained. Similarly, the glutaminase activities as well as the LRFs showed comparable values in Tris, HEPES and Bis-Tris buffer, whereas they dropped in potassium phosphate and MOPS buffer (Figure S24).

These findings corroborated that asparagine hydrolysis shows generally poor light-responsiveness (LRF 1–2.5), whereas glutamine turnover can be reproducibly controlled. Interestingly, while the asparagine reaction retained its activity in various reaction conditions, the glutaminase reaction was highly sensitive with regard to the catalytic strength as well as the photocontrol efficiency (LRF 1.5–9).

### Reversibility of Photocontrol in Both EcAII-Photoxenases

The main advantage of photoswitchable UAAs for the engineering of photoxenases, is the reversibility of photocontrol. Hence, we evaluated whether AzoF is able to switch repeatedly between its PSS^365^ and PSS^420^ in EcAII and whether the photoxenases are able to respond to these repeated switches with a consistent change in activity.

**Figure 4:**
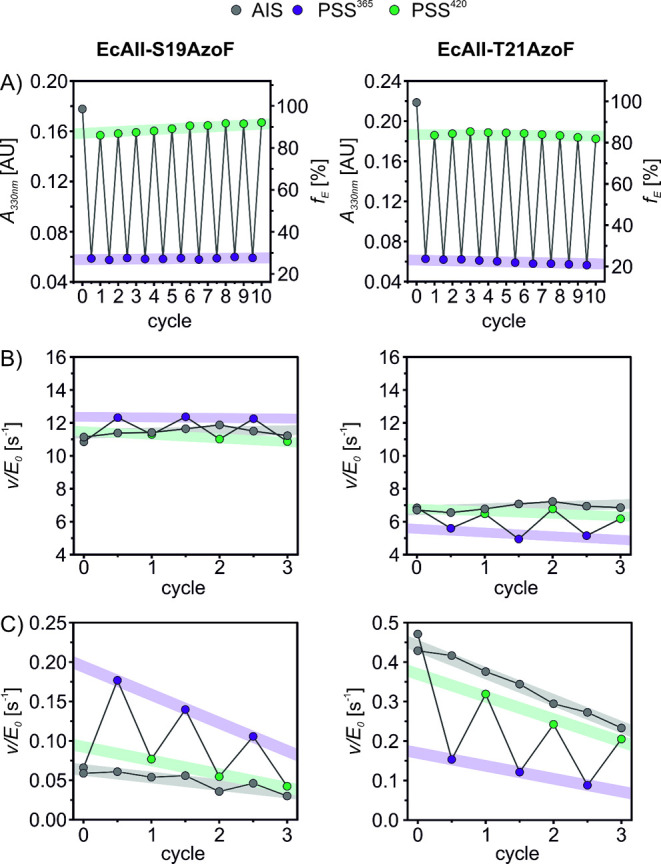
Cycle performance of EcAII-S19AzoF and EcAII-T21AzoF. A) Repeated irradiation of EcAII and measurement of UV/vis spectra indicate a stable reversibility over ten cycles of AzoF isomerization. For details see Figure S25. B) Repeated irradiation during the turnover measurement demonstrates a good reversibility of the minimal photocontrol over the asparaginase activity. For details and second replicate see Figure S26. C) Repeated irradiation during the turnover measurement shows a good reversibility of photocontrol over the glutaminase activity. Note the loss of activity in the AIS that was kept in the dark, which was similarly pronounced for WT-EcAII (gray). For details and second replicate see Figure S27 and S29.

We recorded UV/vis spectra of both photoxenases in their AIS and throughout ten cycles of alternating 365 and 420 nm irradiation (Figure S25). We then plotted the absorbance values of the AzoF ππ* peak at 330 nm, which is indicative of the *E* isomer fraction (*f*_*E*_), against the cycle number (Figure 4A). As a result, both PSSs showed consistent *f*_*E*_ values upon repeated irradiation in the range of 27–29% (EcAII-S19AzoF) and 21–24% (EcAII-T21AzoF) for PSS^365^, and 86–92% (EcAII-S19AzoF) and 82–85% (EcAII-T21AzoF) for PSS^420^.

This allowed us to further investigate the cycle performance of photocontrol for the asparaginase and glutaminase reaction of both photoxenases. For this, we used the same experimental setup as described above, in which we interrupted the turnover measurements and irradiated the reaction throughout three cycles of alternating 365 and 420 nm (cf. Figure 3). We also performed the assays with asparagine (>80•*K*_*m*_) and glutamine (>30•*K*_*m*_) in saturation resulting in a pseudozero order reaction, for which we expect a quasi-linear reaction course. As control, we included a sample for each EcAII variant that was kept in the dark keeping its AIS throughout the reaction progress (“as-isolated reaction”). Each reaction cycle was then fitted with a linear regression model after the steady state was established to obtain *v*/*E*_*0*_ values, which were plotted against the cycle number (Figure 4B). The as-isolated asparaginase reactions demonstrated consistent activities over all cycles for both photoxenases. As expected, the activities of the irradiated sample switched reversibly between lower and higher values after each irradiation step with minor LRFs of ∼1.1 (EcAII-S19AzoF) and ∼1.3 (EcAII-T21AzoF). These results were confirmed in a biological replicate of measurements (Figure S26A,B). Moreover, we repeated the same experiment for WT-EcAII, which further endorsed that its activity is unaffected by irradiation (Figure S26C,D). Interestingly, the as-isolated glutaminase reactions followed an exponential instead of a linear course (Figure S27). We excluded that this effect is caused (i) by the auxiliary enzymes through careful adjustment of the coupled enzymatic assay to reduce the lag phase and through comparison to the glutaminase reaction of ImGPS, which remains linear, or (ii) by an inhibitory effect of α-ketoglutarate (Extended Text S3 and Table S5; Figure S28). Hence, we assume that the exponential behavior is directly associated with the glutaminase activity of EcAII. By plotting the obtained apparent *v*/*E*_*0*_ values of the as-isolated glutaminase reaction against the cycle number, the exponential course of the progress curve translated into a linear decline (Figure 4C). Accordingly, the activities of the irradiated sample switched reversibly between lower and higher values after each irradiation step along this decline in both EcAII-S19AzoF (LRF ∼ 2.3) and EcAII-T21AzoF (LRF ∼ 2.5). This was confirmed in a second replicate of measurements (Figure S29A,B). Again, the activity of WT-EcAII remained unaffected by irradiation (Figure S29C).

With this, we confirmed that AzoF switches between PSSs of consistent *E*:*Z* compositions, which translates into a comparably reversible photocontrol of asparaginase activities with minor LRFs and glutaminase activities with higher LRFs.

### Correlation of Photocontrol Efficiency with Conformational Traits of EcAII

**Figure 5:**
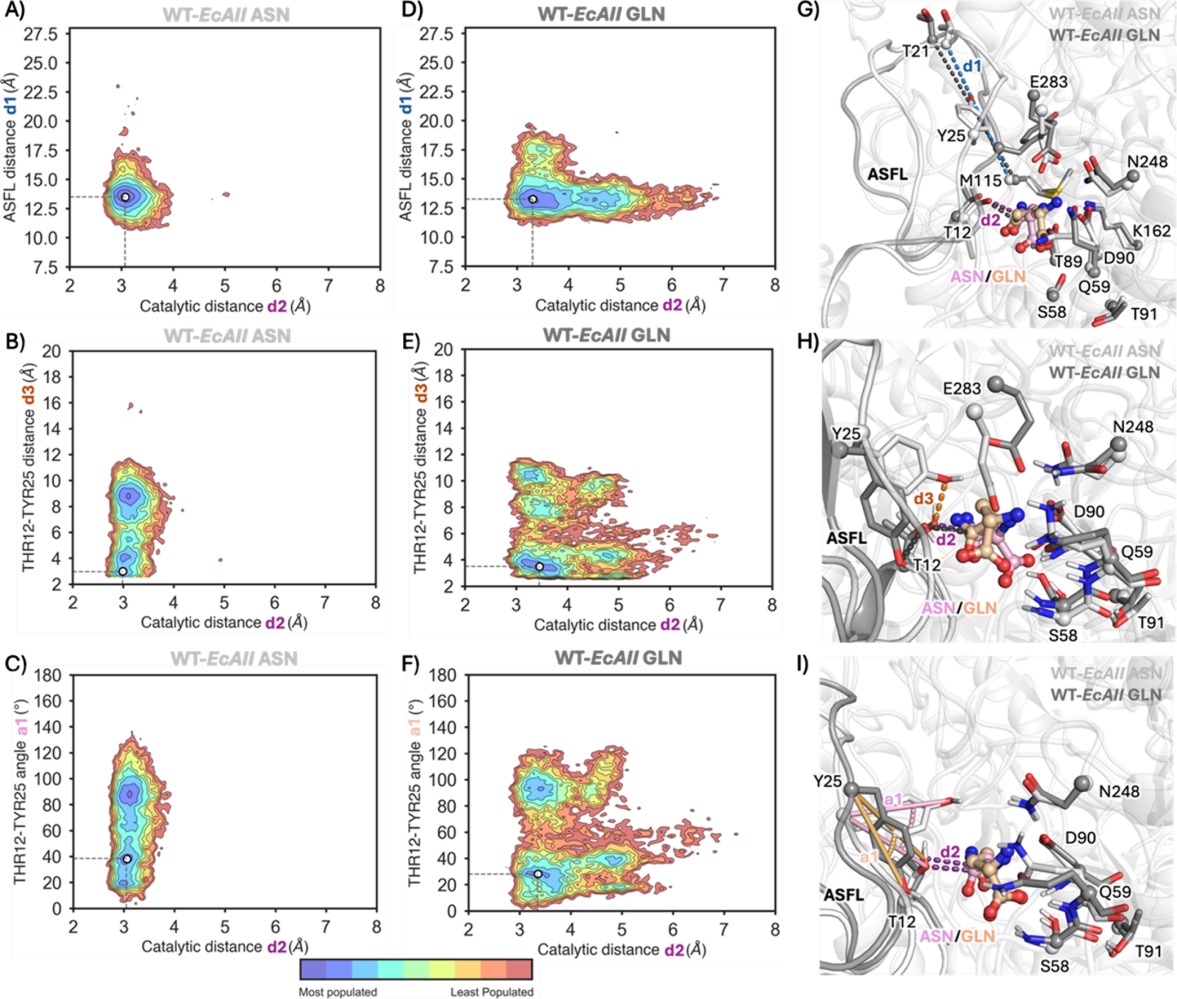
Reconstructed conformational landscapes for WT-EcAII. (A–F) Conformational landscapes derived from MD simulations for WT-EcAII in the presence of asparagine (A–C) and glutamine (D–F). The conformational landscapes are reconstructed based on the nucleophilic attack distance d2 between T12 and asparagine/glutamine and either: distance d1 (T21-M115) representing the ASFL closed-to-open conformation (A, D), the catalytic Y15-T12 distance d3 (B, E), or angle a1 between Y25 and T12 (C, F). (G–I) Overlay of a representative structure extracted from the most populated minimum (white dot in A–F) in the presence of either asparagine (protein in light gray, asparagine in pink) or glutamine (protein in dark gray and glutamine in orange). Asparagine and glutamine are shown in a ball-and-stick representation.

The combination of negligible photocontrol for one reaction and significant photocontrol for another reaction within the same enzyme environment made EcAII an excellent model to deepen our understanding of the requirements for successful photocontrol of enzyme targets with photoswitchable UAAs. Following our original hypothesis, we considered conformational differences to be the cause of this effect. Hence, we performed molecular dynamics (MD) simulations with an initial focus on WT-EcAII in the presence of either asparagine or glutamine. We then reconstructed the conformational landscape of the active site region considering the primary changes in *k*_*cat*_ and *k*_*cat*_/*K*_*m*_, but not in *K*_*m*_, upon irradiation, which imply an interference of AzoF with the chemical step and the substrate-induced closure of the ASFL, but not substrate binding. For this, we regarded four critical angle/distances (cf. Figure S1): (i) distance d1 between T21 and M115 at the active site related to the closed-to-open transition of the ASFL; (ii) distance d2 between T12 and the carbonyl group of either substrate relevant for the nucleophilic attack; and iii/iv) distance d3 and angle a1 between Y25 and T12 facilitating the catalytically important deprotonation of T12. As a result, the reconstructed conformational landscapes for asparagine-bound WT-EcAII demonstrate that the ASFL maintains a closed conformation (d1 < 15 Å), that asparagine is well retained in the active site pocket with short catalytically productive distances d2 and that Y25 can be properly positioned for T12 deprotonation despite displaying a great flexibility (Figure 5A–C). As suggested previously,^34^ glutamine binding instead results in a higher flexibility of the ASFL destabilizing the closed state, an enlarged nucleophilic attack distance d2, but a similar deprotonation distance d3 (Figure 5D–F). Moreover, representative structures extracted from the most populated minimum of asparagine- or glutamine-bound WT-EcAII show a different conformation of the ASFL favoring a different disposition of Y25 for T12 deprotonation (Figure 5G–I). Altogether these data indicate a higher conformational heterogeneity of EcAII in the presence of glutamine instead of asparagine, which might ease a conformational modulation by incorporation and/or isomerization of AzoF.

**Figure 6:**
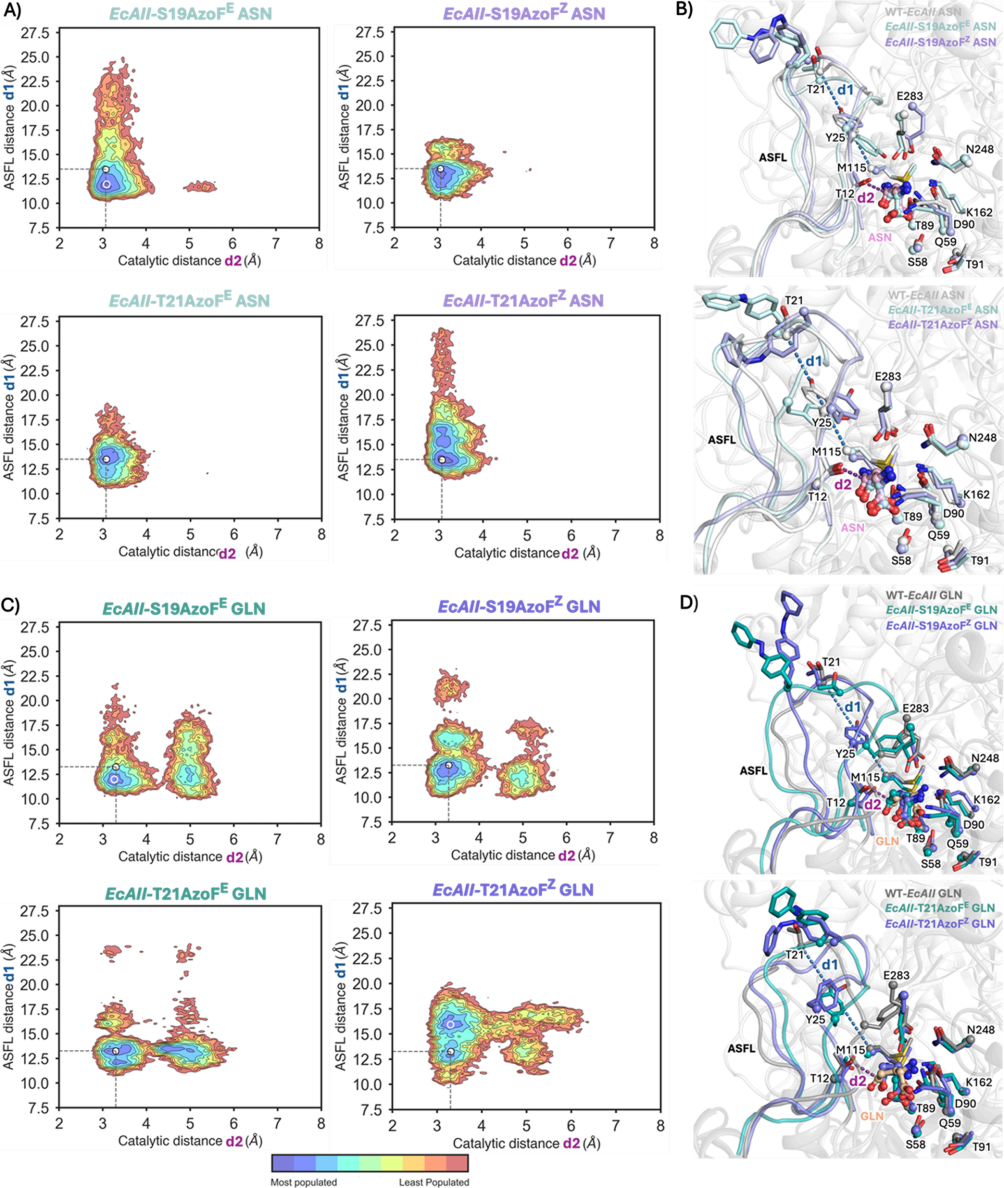
Reconstructed conformational landscapes for asparagine-bound (A, B), and glutamine-bound (C, D) EcAII-photoxenases. (A, C) Conformational landscapes derived from MD simulations using distances d1 and d2. As a reference, the position of the minima found in the case of WT-EcAII is marked using a white dot. (B, D upper panels) Overlay of a representative structure extracted from the most populated minimum (white non-filled circle) for EcAII-S19AzoF^E^ (cyan), EcAII-S19AzoF^Z^ (purple) and WT-EcAII (gray) as reference. (B, D lower panels) Overlay of a representative structure extracted from the most populated minimum (white non-filled circle) for EcAII-T21AzoF^E^ (cyan), EcAII-T21AzoF^Z^ (purple) and WT-EcAII (gray) as reference. Asparagine and glutamine are shown in a ball-and-stick representation.

Thus, we *in silico* incorporated either the *E* or *Z* configuration of AzoF into positions S19 and T21 next. As a result, EcAII-S19AzoF^E/Z^ and EcAII-T21AzoF^E/Z^ contain 100% *E/Z* isomer and are indicative of either the *E*-enriched AIS and PSS^420^ or the *Z*-enriched PSS^365^, respectively, that we used in our experimental analyses. Evaluation of the same four critical angle/distances for the asparagine-bound photoxenases revealed overall very similar conformational landscapes to WT-EcAII (Figure 6A; Figure S30A,B; Figure S31A,B). Higher flexibilities of the ASFL (d1) for EcAII-S19AzoF^E^ and EcAII-T21AzoF^Z^ thereby correlate with the minimally reduced activities of EcAII-S19AzoF in its AIS (compared to PSS^365^) and EcAII-T21AzoF in its PSS^365^ (compared to the AIS). Intriguingly, analysis of the four critical angle/distances for the glutaminase-bound photoxenases demonstrated conformational landscapes that strongly deviated from WT-EcAII (Figure 6C; Figure S32A,B; Figure S33A,B). In fact, conformational heterogeneity was so far enhanced that it was difficult to correlate the conformational landscapes of the *E* and *Z* states with the catalytic activity of the *E*-enriched AIS and *Z*-enriched PSS^365^. Finally, we evaluated our MD simulations regarding a possible explanation for the 2-fold decreased asparaginase *k*_*cat*_ of EcAII-T21AzoF as well as the 12-fold reduced glutaminase *k*_*cat*_ of EcAII-S19AzoF. Interestingly, we found significant deviations in the ASFL and Y25 conformation in the overlay of representative structures from the most populated minima that result in less productive conformations (Extended Text S4; Figure 6B,D; Figures S30–S35).

## Discussion

The engineering of photoxenases is an emerging subdiscipline of optochemistry, which facilitates the reversible regulation of enzyme activity with photoswitchable unnatural amino acids. However, the lack of knowledge on the basic principles of this method limits its applicability. Within this work, we have therefore focused on a key aspect of successful photoxenase engineering, the requirements on the enzyme target. For this, we have used the chemotherapeutic EcAII as model enzyme and could identify two photoxenases, EcAII-S19AzoF and EcAII-T21AzoF, in which AzoF is positioned within the ASFL.

Both photoxenases showed minor photocontrol efficiencies for the main asparaginase reaction (LRFs 1–2), but exhibited higher photocontrol efficiencies for the promiscuous glutaminase reaction, which notably fluctuated in the same reaction conditions (pH 7.0 and 0 mM salt) for both EcAII-S19AzoF (LRF 2–3) and EcAII-T21AzoF (LRF 3–9). While activity related fluctuations are often found when using enzymes purified from different expression harvests, we observed various factors that might additionally influence the photocontrol effect. First, apo WT-EcAII reacted sensitive toward irradiation, which we correlated with partial denaturation and a potential light-induced cleavage of the disulfide bond. However, this effect was negligible for irradiated holo WT-EcAII in substrate excess suggesting that substrate binding might stabilize the enzyme. Second, the presence of cations, monovalent less than divalent and Mg^2+^/Ca^2+^ less than Zn^2+^, hampered EcAII activity as well as its photocontrol, which coincides with previous reports.^35,36^ This inhibition might be explained by a cation binding site that was previously identified in crystal structures of Zn^2+^ bound EcAII^37,38^ and that appears to be allosterically connected to the catalytic residues T89, D90 and K162 via an α helix and a β sheet, respectively (Figure S23E). Initial measurements revealed that catalysis is affected by a coupled V- and K-type allosteric inhibition in physiological conditions (Figure S36). Hence, the presence of residual cations bound to EcAII after purification might be one explanation for the fluctuations in activity and LRF. Alternatively, the fluctuations might be related to the exponential behavior during glutamine turnover in substrate saturation. Based on our experimental experience with EcAII and ImGPS, we excluded the limitation of auxiliary enzymes or substrates, an overly large lag phase as well as inhibition by the final product α-ketoglutarate as the cause of this. Another possible explanation could be the presence of a slow binding inhibitor, which is able to change the shape of the progress curve. For this, glutamate is a possible source of inhibition, because it was previously shown that it can form the tetrahedral intermediate with a homologue of EcAII,^39^ and because it is present in low but substantial steady-state concentrations (low μM range according to reaction simulations with COPASI; https://copasi.org/). Increasing the GOX concentration, which means lowering the steady-state concentration of glutamate, in fact reduced the exponential behavior. Potential glutamate inhibition would further account for fluctuations within biological replicates owing to the high glutamate concentrations of up to 150 mM^40^ in *E. coli* cells. While the exact source and mechanism of the exponential progress curve remains to be investigated, the time span between irradiations was too small for a more accurate exponential fit forcing us to rely on the determination of *v* via a linear regression, which in this case is more prone to errors and might importantly contribute to the fluctuations in photocontrol efficiency. Despite these fluctuations, photocontrol of EcAII-S19AzoF and EcAII-T21AzoF was reproducibly reversible over several cycles of irradiation demonstrating a robust photocontrol system.

One reason why we chose EcAII as target for photoxenase engineering was its well-known induced fit mechanism between the enzyme E, the substrate S and the product P (eq 1).
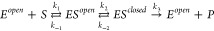
1Previous presteady state investigations^20^ thereby clarified that the rate of ASFL opening *k*_*–2*_ is slower than the chemical step *k*_*3*_ in asparagine hydrolysis so that *k*_*cat*_ = *k*_*3*_.^41^ In the simplest scenario, isomerization of AzoF would only hamper one of the three steps in eq 1. Indeed, our asparaginase steady state kinetics showed that photocontrol of EcAII-T21AzoF resulted primarily from changes in *k*_*cat*_ and hence *k*_*3*_. Analysis of the more light-responsive glutaminase reaction, which included an alteration of *k*_*cat*_ and *k*_*cat*_/*K*_*m*_, would be even more informative, however, the exact catalytic mechanism remains hitherto unclarified. Nevertheless, we could substantiate our hypothesis that photocontrol with AzoF is caused by light-induced conformational transitions within an enzyme. In this regard, we observed a shift of the conformational landscape comparing the 100% *E* with the 100% *Z* state of substrate bound EcAII-S19AzoF and EcAII-T21AzoF. The shifts particularly comprised changes in the closed-to-open transition of the ASFL and the catalytically relevant distances of T12 to asparagine/glutamine and Y25 to T12. Notably, these shifts were more complex in both glutamine-bound EcAII-photoxenases compared to the asparagine-bound systems, which correlates with its higher photocontrol potential. Based on this complexity, we assume that the light-induced isomerization of AzoF might lead to changes in more than one step of the catalytic mechanism that should be addressed in future kinetic and computational studies.

Most importantly, the comparison between asparagine- and glutamine-bound EcAII confirmed our findings in ImGPS that an enzymatic system with higher conformational heterogeneity is more prone toward photocontrol after incorporation of AzoF. Future engineering endeavors might thus be eased by choosing enzyme scaffolds with high conformational heterogeneity, which could be determined via MD simulations beforehand.^42^ In fact, our findings suggest that particularly enzymes that comprise an ASFL with a catalytic induced fit mechanism might be good targets. Furthermore, our results suggest a high success rate by limiting the selection of positions for AzoF incorporation to regions of high conformational heterogeneity. Specifically for enzymes with a substrate-induced ASFL closure, incorporation position within the loop might be the first choice.

Finally, the two photoxenases might benefit potential photocontrolled chemotherapy approaches as they allow for the spatially regulated stimulation of the therapeutic activity at the tumor site, while preventing side effects resulting from enzymatic activity in other parts of the body. The development of such systems is supported by light delivery approaches from the field of phototherapy, e.g., millimeter small and wireless electronic devices that can be injected with a syringe.^43,44^ Particularly, EcAII-S19AzoF is a good starting point for further enhancements toward an application in clinical studies since its activity is increased upon irradiation. These enhancements might thereby include the use of photoswitches that react to visible light and return thermally to the *E* isomer to prevent the systemic spreading of the activated photoxenase, as well as the reduction of sensitivity toward high salt concentrations, e.g., by reducing the affinity of cations via site-directed mutagenesis of the cation binding site.

In summary, our extensive biophysical and computational investigations facilitated a better understanding of photocontrol with photoswitchable UAAs, which promotes an improved design approach of photoxenases for various applications including biotherapy and biocatalysis, while setting the first step toward photocontrolled chemotherapy to reduce side effects of EcAII-based drugs.

## Experimental Section

### Strains, Expression Vectors, Enzymes, Chemicals and Irradiation Devices

Both expression strains were purchased from New England Biolabs (*E. coli* Shuffle T7 Express and *E. coli* NEB Turbo). The expression vectors were designed and produced as described below. The plasmid pEVOL_AzoF for incorporation of AzoF into proteins was provided by P. Schultz (Scripps Research Institute, La Jolla, CA).^7^ Glutamate dehydrogenase (GDH) was purchased from Roche Diagnostics. Glutamate oxidase (GOX) and horseradish peroxidase type I (HRP) were purchased from Sigma-Aldrich. AzoF and ProFAR were synthesized as described previously,^18^ for which the identity of products was determined through ^1^H NMR and the purity of >99% was determined by analytical HPLC. All other reagents and solvents were purchased in analytical grade or higher from commercial sources. Irradiation was performed using either a 365 nm LED (LED Engin Q65113A2058; settings: 850 mA, 20 V) or a 420 nm LED (Avonec 1W410420m; settings: 350 mA, 9 V). Intensities of irradiation were determined with a RM-12 radiometer and UVA+ sensor (Optysec; range: 0–2000 mW cm^–2^) to be 250 mW cm^–2^ (365 nm) and 60 mW cm^–2^ (420 nm).

### Subcloning of the ansB Gene

The gene encoding WT-EcAII from *E. coli* (Uniprot-ID: P00805) was purchased with BsaI cloning sites from GeneArt (Thermo Fisher Scientific) and inserted into the pET28a_BsaI vector^45^ via Golden Gate cloning.^46^ The final plasmid pET28a_EcAII was checked by Sanger Sequencing (Microsynth Seqlab) starting from the T7 promoter and terminator.

### Site-Directed Mutagenesis of the ansB Gene

The introduction of amber stop codon point mutations into the *ansB* gene in various positions was based on the protocol of the Phusion site-directed mutagenesis kit from Finnzymes (Thermo Fisher Scientific) with 5′-phosphorylated and HPLC-purified primers (Metabion). The polymerase chain reaction step was thereby performed either with Phusion or Q5 High-Fidelity DNA Polymerase (New England Biolabs). Correct mutagenesis was checked by Sanger Sequencing (Microsynth Seqlab) starting from the T7 promoter.

### Heterologous Gene Expression and Purification of EcAII

Production of WT-EcAII was performed in *E. coli* Shuffle T7 Express cells by heterologous gene expression. The cells containing pET28a_EcAII were grown in 600 mL of terrific broth (TB) medium at 30 °C until an OD¬600 of ∼5 was reached. Then, expression was induced by addition of 2 mM isopropyl β-d-thiogalactopyranoside (IPTG). After an incubation overnight at 30 °C, the cells were harvested by centrifugation at 4 °C and resuspended in 50 mM Tris/HCl (pH 8.0), 200 mM NaCl, 5% glycerol, and 10 mM imidazole. After sonication and repeated centrifugation steps, EcAII was purified from the supernatant using nickel-affinity chromatography (HisTrap FF Crude column, 5 mL, GE Healthcare). Elution was performed with a linear gradient of imidazole (10→750 mM). Fractions containing EcAII were identified by sodium dodecyl sulfate polyacrylamide gel electrophoresis (SDS-PAGE), pooled, and concentrated. EcAII was further purified using preparative size-exclusion chromatography (Superdex 200 HiLoad 26/600, GE Healthcare) at 4 °C. Fractions of EcAII were eluted with 50 mM Tris-HCl (pH 8.0), 100 mM NaCl, analyzed by SDS-PAGE, pooled, concentrated, and dripped into liquid nitrogen for storage at – 70 °C.

For the production of EcAII-AzoF variants a slightly adjusted protocol was used. Preparation of the expression strain thereby included the cotransformation of the respective pET28a_EcAII-AzoF plasmid and pEVOL_AzoF, encoding the required aaRS/tRNA pair. Furthermore, for the induction of *ansB* expression with 2 mM IPTG was supplemented with 0.25 mM AzoF and 0.02% l-arabinose for the induction of *aaRS* expression. Finally, since preparative size exclusion chromatography could not significantly increase the purity of WT-EcAII and led to a considerable reduction of protein yields, we decided to omit this step for the EcAII-AzoF variants. Instead, we performed dialysis with 50 mM Tris-HCl (pH 8.0), and 100 mM NaCl at 4 °C.

The two ImGPS subunits HisH and HisF were produced following a similar protocol for metal-affinity chromatography as described in detail elsewhere.^17,18^

### Multiple Sequence Alignment

To obtain the genes encoding for periplasmic l-asparaginase II (EC 3.5.1.1) from different organisms, the KEGG GENES database was used. The first 1000 sequence entries were used to create the alignment. The sequence logo was generated with WebLogo.^47,48^

### Asparaginase Activity Measurements in Dependence of Enzyme Concentration

WT-EcAII and EcAII-AzoF variants were diluted to various concentrations between 0.02 μM and 2 μM in Tris/HCl (pH 7.0) and placed in neighboring wells of a transparent 96 well conical bottom plate with a final volume of 80–100 μL. Next, 25–30 μL were aliquoted into 0.2 mL tubes and kept in the dark to retain their AIS. The remaining 55–70 μL in the 96 well plate were then irradiated with 365 nm for 2 s per well to establish the PSS^365^. After aliquoting another 25–30 μL of the proteins in their PSS^365^, the remaining samples in the 96 well plate were irradiated with 420 nm for 30 s per well to establish the PSS^420^. The irradiation durations of 2 s (365 nm) and 30 s (420 nm) were chosen from experience with other AzoF containing proteins and are specific for the chosen LEDs. Asparaginase activity was determined in a coupled enzymatic assay with glutamate-dehydrogenase (GDH, Sigma-Aldrich: Cat#10197734001) as auxiliary enzyme and NADH as cosubstrate (Figure S4A). For this, all reaction components except for the enzymes were prepared in a master mix and incubated for 45 min at room temperature to reduce possible l-aspartate contaminations: 5 mM l-asparagine, 0.25 mM NADH, 5 mM α-ketoglutarate and 20 U/mL GDH in 50 mM Tris/HCl (pH 7.0). Subsequently, 190 μL master mix were aliquoted into neighboring wells of a transparent 96 well flat bottom plate and its baseline absorbance was measured for several minutes at 340 nm and 37 °C using a plate reader (Tecan Infinite M200 Pro; bandwidth: 9 nm, number of flashes: 10). The reaction was initiated by addition of 10 μL enzyme prepared as described above. By this, the enzyme concentration was further diluted to values between 0.001 μM and 0.1 μM. The reactions for each enzyme concentration were measured in technical duplicates at 340 nm and 37 °C for ∼1.5 h. To determine the activity value *v* the initial quasi-linear phase of each reaction was fitted with a linear regression model. The resulting slopes *m* and their standard error of fit (SE) were converted into *v* ± SE (μM/s) using the Lambert–Beer eq (eq 2),
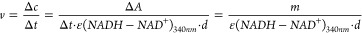
2with *Δc* as the change in NADH concentration over time (*Δt*), *ΔA* as the corresponding change in absorbance, *ε(NADH–NAD*^*+*^*)*_*340 nm*_ as the differential extinction coefficient of NADH (substrate) and NAD^+^ (product) with the value 6300 M^–1^ cm^–1^, and *d* as the path length with an approximate value of 5.35 mm as calculated from technical specifications of the 96 well plate. The obtained *v* ± SE (μM/s) were then plotted against each enzyme concentration. The duplicate values for each enzyme state were fitted together in a concatenated linear regression including a direct weighting to consider the SE of each *v* value. The slope *m′* ± SE of this fit corresponded to the *v/E*_*0*_ ± SE (s^–1^) values. Finally, the LRFs comparing the AIS and the PSS^365^ or PSS^365^ and PSS^420^, respectively, were determined by selecting the respective data set of duplicates (AIS, PSS^365^, PSS^420^) and fitting them with a global fit analysis using a linear regression interaction model (eq 3) and direct weighting.
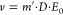
3

The slope *m′* was shared in the global fit and the dummy constant *D* of the slower reaction was fixed to 1. The fitted *D* ± SE value of the faster reaction then corresponded to the LRF ± SE as defined by eq 4,
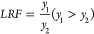
4with *y* as the respective activity constant (*v*, *v/E*_*0*_, *k*_*cat*_, *k*_*cat*_*/K*_*m*_ or *K*_*m*_).

### Glutaminase Activity Measurements in Dependence of Enzyme Concentration

WT-EcAII and EcAII-AzoF variants were diluted to various concentrations between 0.2 μM and 10 μM in Tris/HCl (pH 7.0) and placed in neighboring wells of a transparent 96 well conical bottom plate with a final volume of 80 μL. Next, 25 μL were aliquoted into 0.2 mL tubes and kept in the dark to retain their AIS. The remaining 55 μL in the 96 well plate were then irradiated with 365 nm for 2 s per well to establish the PSS^365^. After aliquoting another 25 μL of the proteins in their PSS^365^, the remaining samples in the 96 well plate were irradiated with 420 nm for 30 s per well to establish the PSS^420^. The irradiation durations of 2 s (365 nm) and 30 s (420 nm) were chosen from experience with other AzoF containing proteins and are specific for the chosen LEDs. Glutaminase activity was determined in a coupled enzymatic assay with glutamate-oxidase (GOX, Sigma-Aldrich Cat#G1924) and horse radish peroxidase (HRP, Sigma-Aldrich Cat#P8250) as auxiliary enzymes and a red-colored quinoneimine as the final product (Figure S5A). For this, all reaction components except for the enzymes were prepared in a master mix and incubated for at least 1 h at room temperature to reduce possible l-glutamate contaminations: 60 mM l-glutamine, 40 U/mL GOX, 109 U/mL HRP, 3 mM 4-aminoantipyrine, and 3 mM phenol in 50 mM Tris/HCl (pH 7.0). Subsequently, 90 μL master mix were aliquoted into neighboring wells of a transparent 96 well flat bottom plate and its baseline absorbance was measured for several minutes at 505 nm and 37 °C using a plate reader (Tecan Infinite M200 Pro; bandwidth: 9 nm, number of flashes: 10). The reaction was initiated by addition of 10 μL enzyme prepared as described above. By this, the enzyme concentration was further diluted to values between 0.002 μM and 1 μM. The reactions for each enzyme concentration were measured in technical duplicates at 505 nm and 37 °C for ∼1.5 h. The activity values *v* and *v/E*_*0*_ as well as the LRF were obtained as described above for the asparaginase activity using the extinction coefficient of 6400 M^–1^ cm^–1^.^49^

### Tryptic Digest and MS Analysis

The identity of EcAII and incorporation of AzoF were verified using mass spectrometry. Recombinant EcAII proteins were run on a 13.5% SDS-PAGE gel and stained with Coomassie (SimplyBlue SafeStain, Lifetech). Protein bands were cut out from the gel, washed with 50 mM NH_4_HCO_3_, a 50 mM NH_4_HCO_3_/acetonitrile mixture (3/1), and a 50 mM NH_4_HCO_3_/acetonitrile mixture (1/1) and lyophilized. After a reduction/alkylation treatment and additional washing steps, proteins were in gel digested with trypsin (Trypsin Gold, mass spectrometry grade, Promega) overnight at 37 °C. The resulting peptides were sequentially extracted with 50 mM NH_4_HCO_3_ and 50 mM NH_4_HCO_3_ in 50% acetonitrile. After lyophilization, peptides were reconstituted in 20 mL of 1% TFA and separated by reversed-phase chromatography. An UltiMate 3000 RSLCnano System (Thermo Fisher Scientific, Dreieich, Germany) equipped with a C18 Acclaim Pepmap100 preconcentration column [100 μm i.d. × 20 mm, Thermo Fisher Scientific] and an Acclaim Pepmap100 C18 nano column [75 mm i.d. × 250 mm, Thermo Fisher Scientific] was operated at a flow rate of 300 nL/min and a 60 min linear gradient of 4% to 40% acetonitrile in 0.1% formic acid. The liquid chromatograph was online-coupled to a maXis plus UHR-QTOF System (Bruker Daltonics, Billerica, MA, USA) via a Captive-Spray nanoflow electrospray source. Acquisition of MS/MS spectra after CID fragmentation was performed in data-dependent mode at a resolution of 60,000. The precursor scan rate was 2 Hz processing a mass range between *m*/*z* 175 and 2000. A dynamic method with a fixed cycle time of 3 s was applied via the Compass 1.7 acquisition and processing software (Bruker Daltonics). Prior to database searching with Protein Scape 3.1.3 (Bruker Daltonics) connected to Mascot 2.5.1 (Matrix Science), raw data were processed in Data Analysis 4.2 (Bruker Daltonics). A customized database comprising the *E. coli* entries from UniProt as well as manually added sequences of the mutated EcAII proteins and common contaminants was used for a database search with the following parameters: Enzyme specificity trypsin with two missed cleavages allowed, precursor tolerance 10 ppm, MS/MS tolerance 0.04 Da. General variable modifications included in the search were deamidation of asparagine and glutamine, oxidation of methionine, carbamidomethylation, or propionamide modification of cysteine. AzoF was detected as a specific variable modification of phenylalanine, why the position of AzoF incorporation was changed to a phenylalanine in the query. Spectra of peptides containing AzoF were inspected manually.

### Circular Dichroism Analysis

Circular dichroism spectra in the far-UV range of 195–260 nm were recorded in a Jasco J-815 spectrophotometer with five accumulations. The spectra were measured with 8.2–10 μM protein in 10 mM potassium phosphate, pH 8.0 in a 0.1 cm cuvette at 37 °C. Data were normalized to obtain the mean residue ellipticity as described by ref.^50^ To follow the temperature dependent unfolding process, melting curves were recorded at 220 nm by heating the sample from 25 to 95 °C at a rate of 1 °C per min. The curves were fitted with the Boltzmann eq 5 with A1 and A2 as values of minimum and maximum intensities, respectively, to determine the denaturation midpoint *T*_*m*_ (plotted and fitted in Origin 2019).
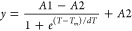
5Another spectrum was recorded after the heating step with the same settings described above to compare the protein folding before and after denaturation.

### Analytical Size-Exclusion Chromatography with Static Light Scattering

40 μM of all EcAII variants were subjected to a S200 10/300 GL (GE Healthcare) column pre-equilibrated in 50 mM Tris/HCl, pH 7.0 and 100 mM NaCl. Samples were eluted in the same buffer, and protein as well as AzoF were detected at 280 and 334 nm, respectively.

### Steady-State Kinetics

For the determination of steady-state constants, we used the same irradiation and reaction conditions as in our screening and chose enzyme concentrations from the lower third of the screened concentration range (cf. Figure S4, Figure S5). EcAII proteins were diluted to 0.2 μM (WT-EcAII and EcAII-S19AzoF) or 0.4 μM (EcAII-T21AzoF) in Tris/HCl (pH 7.0) for the asparaginase steady-state kinetics, or to 1 μM (WT-EcAII) or 3 μM (EcAII-S19AzoF, EcAII-T21AzoF) in Tris/HCl (pH 7.0) for the glutaminase steady-state kinetics. For each variant and each reaction the samples were then divided. One part was aliquoted into 0.2 mL tubes and kept in the dark to maintain the AIS. The second part was aliquoted into a transparent 96 well conical bottom plate and irradiated with 365 nm for 2 s per well to establish the PSS^365^. The third part was aliquoted into the same 96 well plate, irradiated with 365 nm for 2 s per well and subsequently with 420 nm for 30 s per well to establish the PSS^420^. Asparaginase activity was determined and *v/E0* values were obtained as described above for the asparaginase activity measurements in dependence of enzyme concentration. Reaction conditions included: 0.005–2 mM l-asparagine, 0.25 mM NADH, 5 mM α-ketoglutarate, 14 U/mL GDH and 0.01/0.02 μM EcAII (20-fold dilution) in 10 mM Tris/HCl (pH 7.0) at 37 °C. Glutaminase activity was also determined and *v/E*_*0*_ values obtained as described above for the glutaminase activity measurements in dependence of enzyme concentration. Reaction conditions included: 0.5 mM l-glutamine, 40 U/mL GOX, 109 U/mL HRP, 3 mM 4-aminoantipyrine, and 3 mM phenol and 0.1/0.3 μM EcAII (10-fold dilution) in 10 mM Tris/HCl (pH 7.0). For both reactions, three technical replicates of the same EcAII variant preparation were measured. The obtained *v/E*_*0*_ ± SE (s^–1^) values were then plotted against the l-asparagine or l-glutamine concentration [*c(S)*]. To determine the *k*_*cat*_ and *K*_*m*_ of each reaction, the triplicate values for each enzyme state were fitted together in a concatenated nonlinear regression model following the Michaelis–Menten law (eq 6) with direct weighting.
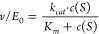
6

The *k*_*cat*_*/K*_*m*_ was obtained by fitting the triplicate values for each enzyme state using a converted version of eq 6 (eq 7).
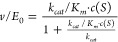
7

The LRFs comparing the AIS and the PSS^365^ or PSS^365^ and PSS^420^, respectively, were determined by selecting the respective data set of triplicates (AIS, PSS^365^, PSS^420^) and fitting them with a global fit analysis using a nonlinear regression interaction model, related to the Michalis-Menten eq (eq 6) and its derivative (eq 7), and direct weighting. The LRF ± SE for *k*_*cat*_ was obtained via eq 8,
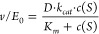
8by sharing *k*_*cat*_ in the global fit and setting the dummy constant *D* of the slower reaction to 1 obtaining the LRF from *D* ± SE of the faster reaction. The LRF ± SE for *K*_*m*_ was obtained via eq 9,
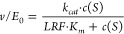
9by sharing *K*_*m*_ in the global fit and setting the dummy constant *D* of the reaction with the lower *K*_*m*_ value to 1 obtaining the LRF from *D* ± SE of the reaction with the higher *K*_*m*_ value. The LRF ± SE for *k*_*cat*_*/K*_*m*_ was obtained via eq 10,
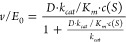
10by sharing *k*_*cat*_^*/*^*K*_*m*_ in the global fit and setting the dummy constant *D* of the slower reaction to 1 obtaining the LRF from *D* ± SE of the faster reaction.

### UV/Vis Analysis

UV/vis spectra of 15 μM EcAII in 10 mM Tris/HCl (pH 7.0) were recorded in a 96 well plate (Greiner Bio-One, UV-Star, Microplate, 96 well, f-bottom, μClear) at 25 °C in the range of 230–600 nm using a Tecan M Nano Platereader. Spectra of EcAII were either measured in its AIS or after subsequent irradiation with 365 nm UV light or 420 nm visible light. Irradiation durations are given in the figure descriptions of each experiment. All spectra were then baseline corrected at 600 nm. To follow the establishment of the PSS, the absorbance values at the ππ* transition (330 nm) were plotted against the irradiation time. The rate of isomerization *k*^365^ was obtained by fitting the decreasing signal points with eq 11
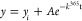
11in which *t* is the irradiation time, *y*_*i*_ is the *y* value at infinite times (also named plateau) and *A* is the span of the exponential curve between *y*_*i*_ and *y*_*0*_ (the *y* value when time = 0). The rate of isomerization *k*^420^ was likewise determined by fitting the increasing signal points with eq 12.
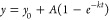
12Finally, the half-time of isomerization *t*_*1/2*_ was derived from the isomerization rates *k* with eq 13.
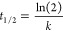
13

### Estimation of the E:Z Ratio from UV/Vis Spectra

The estimation of *E*:*Z* distributions for EcAII-S19AzoF and EcAII-T21AzoF was based on previously published protocols^51,52^ and is shown exemplarily in Figure S10. First, several UV/vis spectra spanning the various time-points between the AIS and the PSS^365^ were chosen from one experiment. The ππ* and nπ* peaks were deconvoluted between 310 and 600 nm using a Gaussian function (“multiple peak fit” tool in Origin 2024). By this, a cumulative Gauss fit of both peaks was produced that simulates the respective UV/vis spectrum. Second, these cumulative Gauss fits were used to determine a first estimate of the *E*:*Z* ratios. For this, the cumulative Gauss fit of the AIS was used as a 100% *E* reference. To obtain the fraction of *E* in another selected cumulative Gauss fit, the spectrum of 100% *Z* was simulated with the “simple curve math” tool in Origin 2024 using eq 14
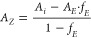
14in which *A*_*Z*_ is the simulated spectrum of the 100% *Z* isomer, *A*_*E*_ is the cumulative Gauss fit of the AIS (100% *E*), *A*_*i*_ is the cumulative Gauss fit of the spectrum in question, and *f*_*E*_ is the fraction of the *E* isomer. *f*_*E*_ was obtained in this simulation by manual adjustment until the signal of the ππ* peak reached zero. Third, we used the estimated *f*_*E*_ values to produce a standard curve for each experiment by plotting the values against the absorbance value at 330 nm from each original UV/vis spectrum. Linear regression of the plotted data finally obtained a linear fit equation that was used to estimate *f*_*E*_ in different evaluations such as the determination of rate constants.

### Asparaginase and Glutaminase Activities via Irradiation during the Measurement

Both reactions were measured using the coupled enzymatic assays described above. For this, all reaction components except for the enzymes were prepared in a master mix and incubated for 45 min to 1 h at room temperature to reduce possible aspartate or glutamate contaminations. The individual reaction compositions are provided in the figure descriptions of each experiment. Each reaction was then initiated by the addition of 10 μL enzyme in its AIS or PSS^3^_a_^6^_p_^5^_o_ using enzyme from the same expression (technical replicates) within each experiment and in part enzymes from different expressions (biological replicates) between each experiment. When the linear steady state phase was reached, the measurements were paused and the reactions were irradiated with either 365 nm for 2 s per 2 wells to establish the PSS^365^ or with 420 nm for 8 s per 2 wells to establish the PSS^420^. In some cases, reactions were run in parallel that were not irradiated. These control reactions were placed in wells of the 96 well plate that were as far away from the irradiated reactions as possible. During irradiation, aluminum foil was positioned above these wells as well as in neighboring wells to protect the reactions from exposure to light. To determine the activity value *v* the initial quasi-linear phase of each reaction was fitted with a linear regression model. The resulting slopes *m* and their standard error of fit (SE) were converted into *v* ± SE (μM s^–1^) using the Lambert–Beer equation (cf. eq 2). Normalization using the applied enzyme concentration obtained *v/E*_*0*_ ± SE (s^–1^) values. The LRFs comparing the AIS and the PSS^365^, the PSS^365^ and PSS^420^, or the PSS^420^ and the PSS^365^, respectively, were calculated from the *v/E*_*0*_ values using eq 2.

### Molecular Modeling System Preparation

The X-ray structure available for WT-EcAII in a closed conformation (PDB code: 3ECA) was used to generate the starting structures for the four systems (EcAII-S19AzoF^E^, EcAII-S19AzoF^Z^, EcAII-T21AzoF^E^ and EcAII-T21AzoF^Z^) with the multimer version of the AlphaFold2 (AF2)^53^ neural network. The AF2 models simulated had a predicted LDDT-Cα score (pLDDT) higher than 94. The water molecules added to each subunit were selected from the X-ray structure available for WT-EcAII in a closed conformation (PDB code: 3ECA). In the MD simulations, the water molecules clashing with the substrate or the protein were removed manually. The MD parameters for the substrates Asparagine and Glutamine and the unnatural amino acid AzoF in *Z* and *E* configurations were generated with the antechamber and parmchk2 modules of AMBER20^54^ using the second generation of the general amber force-field (GAFF2).^54,55^ The substrates and unnatural amino acid were optimized at the B3LYP/6–31G(d)^56,57^ level of theory including Grimme’s dispersion correction with Becke-Johnson Damping (D3-BJ)^58^ and the polarizable conductor model (PCM) with water as the solvent using Gaussian16.^59^ The partial charges (RESP model)^60^ were set to fit the electrostatic potential generated at the HF/6–31G(d) level of theory. The charges were calculated according to the Merz–Singh–Kollman^61^ scheme using Gaussian16.^59^ The protonation states were predicted using PROPKA.^62,63^ The enzyme structures were solvated in a pre-equilibrated truncated octahedral box of 12 Å edge distance using the OPC water model and neutralized by the addition of explicit counterions (i.e., Na^+^) using the AMBER20 leap module. All MD simulations were performed using a modification of the amber99 force field (ff19SB).^64^

### MD Simulation Details

MD equilibration phase was done following the protocol described by Roe and Brooks with small differences fine-tuned to our systems.^65^ The bonds involving hydrogen are constrained by the SHAKE algorithm during the nonminimization steps. Long-range electrostatic effects were modeled using the particle mesh-Ewald method.^66^ For Lennard–Jones and electrostatic interactions, a 10 Å cutoff was applied. The MD protocol starts with the minimization phase of 1500 steps of the steepest descent method followed by 3500 steps of the conjugate gradient method with a positional restrain (i.e., a force constant of 5.0 kcal·mol^–1^·Å^–2^) to the protein heavy atoms. In the following heating phase a temperature increment from 25 to 300 K during 20 ps of MD simulation time, a Langevin thermostat with a collision frequency of 5 ps^–1^, and a positional restrain (i.e., a force constant of 5.0 kcal·mol^–1^·Å^–2^) to the protein heavy atoms; are performed. A minimization and heating of all atoms in the system is the following step. This starts with two minimization stages of 1000 steps of the steepest descent method followed by 1500 steps of the conjugate gradient method each with a positional restrain (i.e., force constant of 2.0 kcal·mol^–1^·Å^–2^ in the first minimization and 0.1 kcal·mol^–1^·Å^–2^ in the second) to the protein heavy atoms. Following, a third minimization phase of 1500 steps of the steepest descent method followed by 3500 steps of the conjugate gradient method without any positional restraint is performed. The system is then heated in accordance with the previously established procedure. Finally, a five-round equilibration phase at the NPT ensemble with a constant pressure of 1 atm is performed. The first four rounds were done with the Berendsen barostat, whereas the fifth one was done with a Monte Carlo barostat. For all equilibration rounds, Langevin thermostat with a collision frequency of 1 ps^–1^ was used. A positional restraint to the protein-heavy atoms with a force constant of 1.0 and 0.5 kcal·mol^–1^·Å^–2^ was applied to the first and second equilibration rounds, respectively. In the third round of 10 ps equilibration, a positional restraint to the backbone-heavy atoms with a force constant of 0.5 kcal·mol^–1^·Å^–2^ was used. The fourth and fifth equilibration of 10 ps and 1 ns, respectively, were performed without any restraint. The production runs were performed at the NVT ensemble with the Langevin thermostat with a collision frequency of 1 ps^–1^ during 200 ns for all systems. A total of three replicas of equilibration and production runs were performed reaching a total simulation time of 0.6 μs/system (3 replicas × 200 ns). The MD trajectories were analyzed using the Python packages MDTraj,^67^ pytraj^68^ which is part of the cpptraj package,^65^ MDAnalysis,^69^ and PyEMMA.^70^

### Conformational Landscape (CL) Reconstruction

Molecular dynamics (MD) simulations allow the sampling of the population distribution of biomolecules by integrating Newton’s laws of motion. However, due to the vast number of atoms involved in the MD simulations, this probability distribution of molecular states is represented in an extremely high-dimensional space. This is usually solved by focusing on a selected set of degrees of freedom (DOF) relevant to the process of interest. In our case we used: the distance between Thr21(C_α_) and Met115(C_α_) for the closed-to-open transition of ASFL, the Thr12(OG1)-Tyr25(OH) distance for Thr12 deprotonation, the angle defined by Thr12(OG1)-Tyr25(C_α_)-Tyr25(OH) (*y* axis) and the distance between Thr12(OG1) and Asparagine or Glutamine (C=O) for the catalytic distance (*x* axis in all Figures). High dimensional data obtained from MD simulations can be projected onto these DOFs for obtaining the probability distributions. So, a maximum in the distribution corresponds to a most frequently visited conformation of the protein.

## Supporting Information Available

Raw data and secondary plots of experimental results as well as in-depth conformational landscapes derived from MD simulations (PDF)
