# Supplementary material for: Reversible Substrate-Specific Photocontrol of the Chemotherapeutic Asparaginase(-Glutaminase) from Escherichia coli
Source: ACS Catal. 2025 May 6;15(10):8462–78. doi: 10.1021/acscatal.5c01608 (PMC12090219; doi:10.1021/acscatal.5c01608)
Supplement: Supplementary file 1 — cs5c01608_si_001.pdf [file cs5c01608_si_001.pdf]

Supporting Information

Reversible Substrate-Specific Photocontrol of the Chemotherapeutic Asparaginase(-Glutaminase) from Escherichia coli

Mona Wieland,[a] Jonnely Luizaga,[b] Cristina Duran,[b] Barbara Germscheid,[a] Johanna Rein,[a] Astrid Bruckmann,[c] Caroline Hiefinger,[a] Sílvia Osuna,[b,d] Andrea Hupfeld[a]*

[a] M. Wieland, B. Germscheid, J. Rein, A. Bruckmann, A. Hupfeld (née Kneuttinger)
Institute of Biophysics and Physical Biochemistry and Regensburg Center for Biochemistry, University of Regensburg, Universitätsstraße 31, D-93053 Regensburg (Germany)
E-mail: andrea.hupfeld@ur.de
[b] J. Luizaga, C. Duran, S. Osuna
Institut de Química Computacional i Catàlisi and Departament de Química, Universitat de Girona, c/ Maria Aurèlia Capmany 69, 17003 Girona (Spain)
[c] A. Bruckmann
Institute of Biochemistry, Genetics and Microbiology, University of Regensburg, Universitatsstrasse 31, D-93053 Regensburg (Germany)
[d] S. Osuna
ICREA, Pg. Lluís Companys 23, 08010 Barcelona (Spain)

Table of Contents

SUPPLEMENTARY MATERIAL FOR THE INTRODUCTION 3

Figure S1. 3

SUPPLEMENTARY MATERIAL FOR THE RESULTS 4

IDENTIFICATION OF ECAII BASED PHOTOXENASES 4
Figure S2. 4
Figure S3. 4
Figure S4. 5
Table S1. 7
Figure S5. 8
Table S2. 10
KINETIC BEHAVIOR OF ECAII-S19AZOF AND ECAII-T21AZOF 11
Figure S6. 11
Figure S7. 12
Figure S8. 12
Figure S9. 13
Figure S10. 14
Figure S11. 15
Figure S12. 16
Table S3. 17
Table S4. 18
Extended Text S1. 19
Figure S13. 20
Extended Text S2. 21
Figure S14. 22
Figure S15. 23
Figure S16. 24
VALIDATION OF SUBSTRATE-SPECIFIC PHOTOCONTROL IN ECAII 25
Figure S17. 25
Figure S18. 27
Figure S19. 29
Figure S20. 30
Figure S21. 31
Figure S22. 31
Figure S23. 32
Figure S24. 33
EVALUATION OF THE REVERSIBILITY OF PHOTOCONTROL 34
Figure S25. 34
Figure S26. 34
Figure S27. 35
Extended Text S3. 35
Table S5. 36
Figure S28. 36
Figure S29. 37
CORRELATION OF PHOTOCONTROL EFFICIENCY WITH CONFORMATIONAL TRAITS OF ECAII 38
Figure S30. 38
Figure S31. 39
Figure S32. 40
Figure S33. 41
Extended Text S4. 42
Figure S34. 43
Figure S35. 44

SUPPLEMENTARY MATERIAL FOR THE DISCUSSION 44

Figure S36. 44

REFERENCES 45

SUPPLEMENTARY MATERIAL FOR THE INTRODUCTION

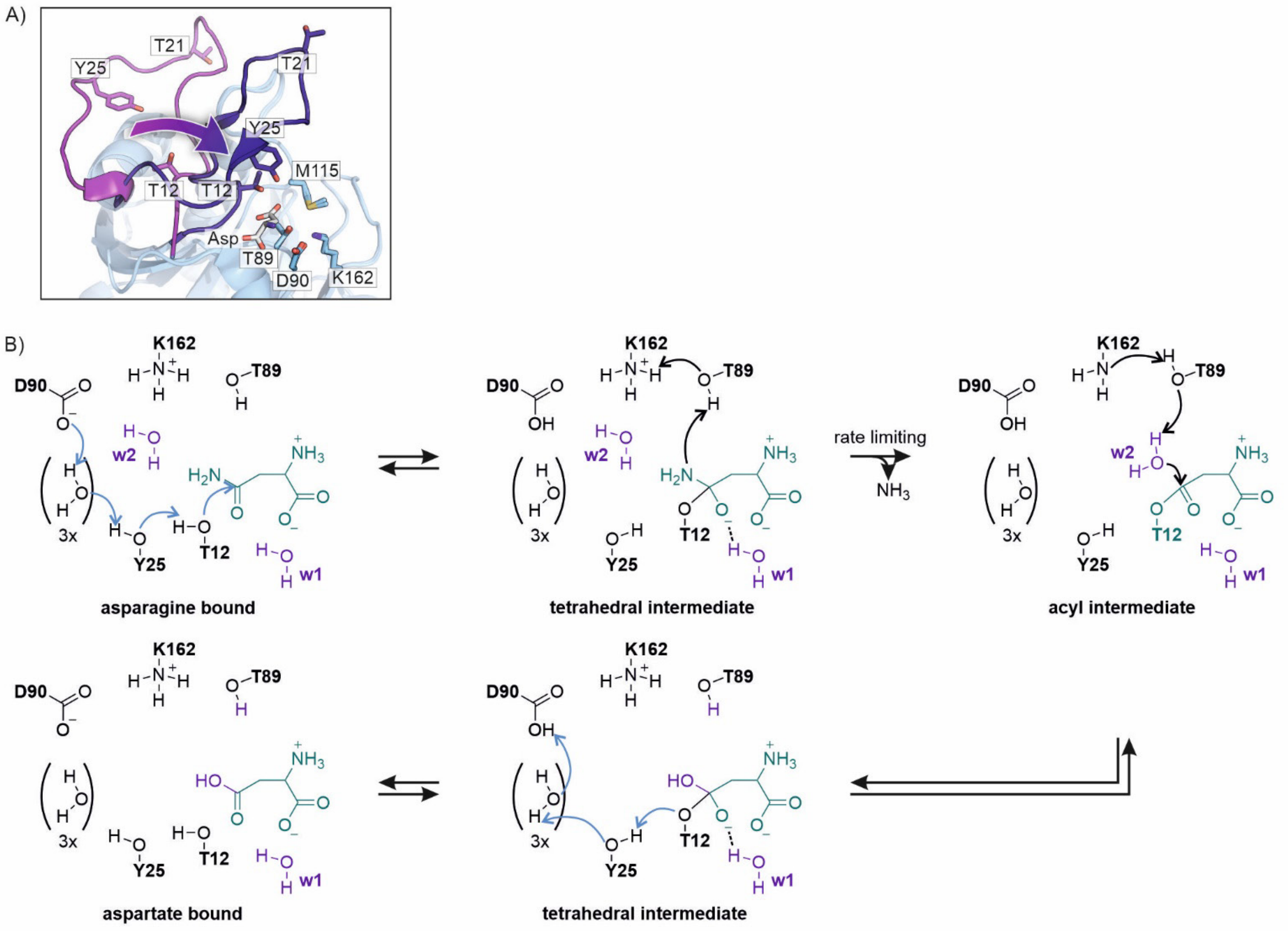

Figure S1. The catalytic mechanism of EcAII. A) Transition of the active site flexible loop (ASFL) in EcAII upon ligand binding and position of catalytic residues. PDB-IDs: 7p9c (open ASFL) and 3eca (closed ASFL). B) Double displacement mechanism of asparagine hydrolysis in EcAII.[1] A "proton shuttle" (blue arrows) from the catalytic residues T12 and Y25 to the catalytic residue D90 is presumed to increase the nucleophilicity of T12 by proton subtraction. Nucleophilic attack of T12 on the carboxamide carbon of asparagine (teal) generates a tetrahedral intermediate, which is stabilized by an oxyanion whole that comprises a conserved water molecule (w1, purple). The catalytic residues T89 and K162 supposedly protonate the amide group to release ammonium and an acyl intermediate in the rate-limiting step. Next, a second conserved water molecule (w2, purple) attacks the acyl intermediate to generate another tetrahedral intermediate. Finally, a "proton shuttle" back to T12 decreases the nucleophilicity of T12 and facilitates the release of the aspartate product.

SUPPLEMENTARY MATERIAL FOR THE RESULTS

Identification of EcAII based photoxenases

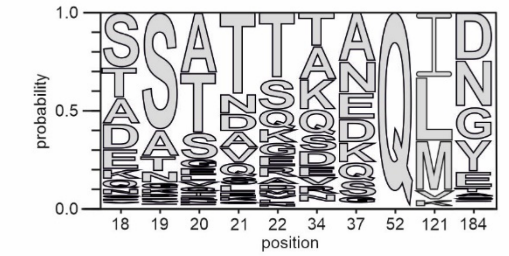

Figure S2. Sequence logo of the selected positions generated from a multiple sequence alignment.

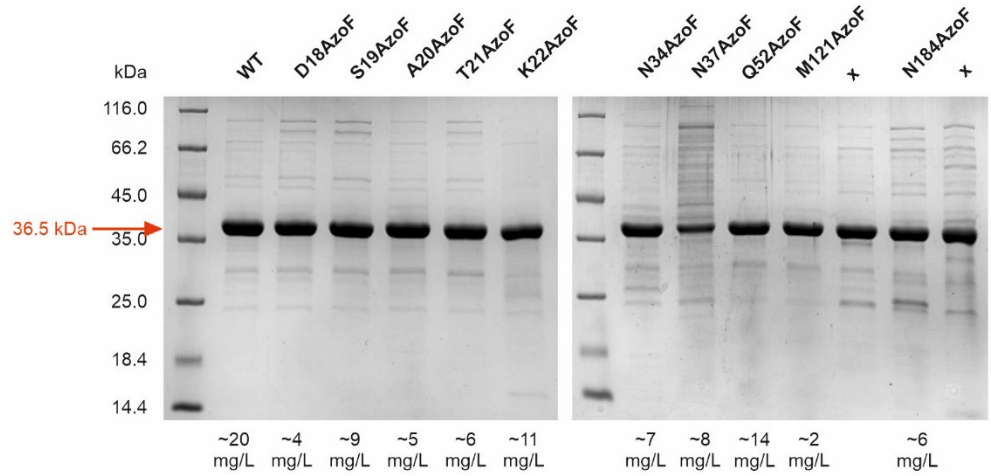

Figure S3. SDS-PAGE (3 µg protein) and purification yields (in mg protein per liter expression culture) of the EcAII variants after heterologous gene expression in *E. coli* and subsequent metal-affinity chromatography. WT-EcAII, D18AzoF, EcAII-S19AzoF, EcAII-A20AzoF, EcAII-T21AzoF, EcAII-K22AzoF, EcAII-Q52AzoF, and EcAII-M121AzoF show a purity of >95%, whereas EcAII-N184AzoF and EcAII-N37AzoF only show purities of >85% and >70%, respectively. Note: "x" marks two lanes with EcAII variants that are not relevant for this study. An additional preparative size exclusion chromatography step could not significantly increase the purity of WT-EcAII and led to a considerable reduction of protein yields. For this reason, we decided to omit this step for the EcAII-AzoF variants and accept the partly poor purity in favor of production yields.

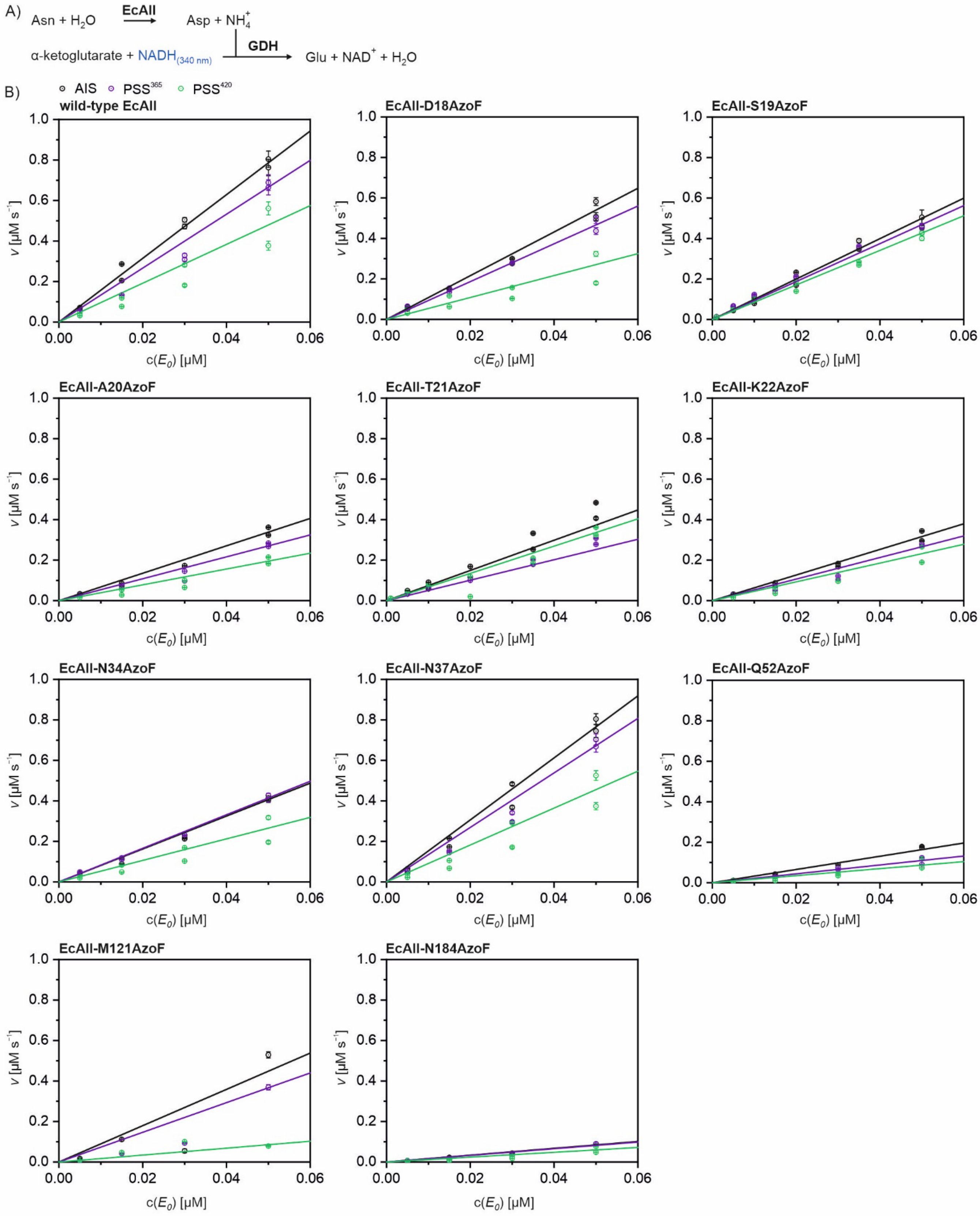

Figure S4. Screening of the asparaginase activity of EcAII-AzoF variants in their AIS, PSS^{365} and PSS^{420}. A) Asparaginase activity was determined by coupling the reaction to glutamate dehydrogenase (GDH) following the absorbance of NADH at 340 nm. B) The initial catalytic rates of all EcAII variants were plotted against the used enzyme concentration to ensure that EcAII is rate-limiting in the coupled enzymatic assay. *Irradiation:* 0.02–2 µM EcAII were either kept in the dark (AIS), irradiated 2 s with 365 nm (PSS^{365}), or irradiated 2 s with 365 nm and 30 s with 420 nm (PSS^{420}). *Reaction conditions:* 5 mM asparagine (saturated), 0.25 mM NADH, 5 mM α-ketoglutarate, 20 U/mL GDH and 0.001–0.1 µM

EcAII in 50 mM Tris/HCl (pH 7.0) at 37°C. *Statistics:* Each circled value represents the fitted initial catalytic rate v ± standard error of fit (SE); two technical replicates were measured for each enzyme concentration (except for EcAII-M121AzoF for which only one replicate for each enzyme concentration was measured).

Table S1. Fitting values and statistics for the asparaginase activity screening of EcAII-AzoF variants in **Figure S4**.

EcAII variant	state	v/E_0 [s^{-1}]	R^2 (v/E_0)	LRF1[a] a.i. → PSS^{365}	R^2 (LRF1)	LRF2[a] PSS^{365} → PSS^{420}	R^2 (LRF2)
WT	a.i.	15.73 ± 0.18	1.00	1.18 ± 0.03 ↓	0.96		
	PSS^{365}	13.31 ± 0.31	1.00			1.39 ± 0.10 ↓	0.85
	PSS^{420}	9.59 ± 0.70	0.96				
D18AzoF	a.i.	10.79 ± 0.30	0.99	1.16 ± 0.04 ↓	0.90		
	PSS^{365}	9.33 ± 0.21	1.00			1.72 ± 0.14 ↓	0.86
	PSS^{420}	5.41 ± 0.52	0.93				
S19AzoF	a.i.	9.99 ± 0.16	1.00	1.07 ± 0.03 ↓	0.98		
	PSS^{365}	9.37 ± 0.16	1.00			1.10 ± 0.02 ↓	0.98
	PSS^{420}	8.54 ± 0.06	1.00				
A20AzoF	a.i.	6.77 ± 0.19	0.99	1.25 ± 0.05 ↓	0.94		
	PSS^{365}	5.41 ± 0.19	0.99			1.38 ± 0.08 ↓	0.91
	PSS^{420}	3.91 ± 0.17	0.99				
T21AzoF	a.i.	7.46 ± 0.15	0.99	1.48 ± 0.05 ↓	0.95		
	PSS^{365}	5.05 ± 0.05	1.00			1.33 ± 0.03 ↑	0.97
	PSS^{420}	6.73 ± 0.12	1.00				
K22AzoF	a.i.	6.33 ± 0.18	0.99	1.19 ± 0.05 ↓	0.93		
	PSS^{365}	5.32 ± 0.17	0.99			1.15 ± 0.08 ↓	0.84
	PSS^{420}	4.64 ± 0.31	0.97				
N34AzoF	a.i.	8.13 ± 0.08	1.00	1.02 ± 0.01 ↑	0.98		
	PSS^{365}	8.29 ± 0.09	1.00			1.56 ± 0.11 ↓	0.85
	PSS^{420}	5.31 ± 0.45	0.94				
N37AzoF	a.i.	15.31 ± 0.34	1.00	1.14 ± 0.04 ↓	0.93		
	PSS^{365}	13.46 ± 0.36	0.99			1.48 ± 0.10 ↓	0.88
	PSS^{420}	9.12 ± 0.59	0.97				
Q52AzoF	a.i.	3.27 ± 0.15	0.98	1.49 ± 0.11 ↓	0.95		
	PSS^{365}	2.19 ± 0.11	0.98			1.26 ± 0.15 ↓	0.90
	PSS^{420}	1.74 ± 0.19	0.91				
M121AzoF	a.i.	8.97 ± 1.95	0.83	1.22 ± 0.33 ↓	0.58		
	PSS^{365}	7.33 ± 0.26	0.99			4.29 ± 1.15 ↓	0.97
	PSS^{420}	1.71 ± 0.27	0.91				
N184AzoF	a.i.	1.69 ± 0.07	0.99	1.03 ± 0.07 ↓	0.96		
	PSS^{365}	1.64 ± 0.08	0.98			1.37 ± 0.17 ↓	0.90
	PSS^{420}	1.20 ± 0.14	0.89				

[a]The activity change is indicated by an upwards (activity decrease) or downwards (activity decrease) arrow. Statistics: v/E_0 and LRF values are given as fitting value ± SE; R^2 represents the corrected R^2 as determined in Origin 2024.

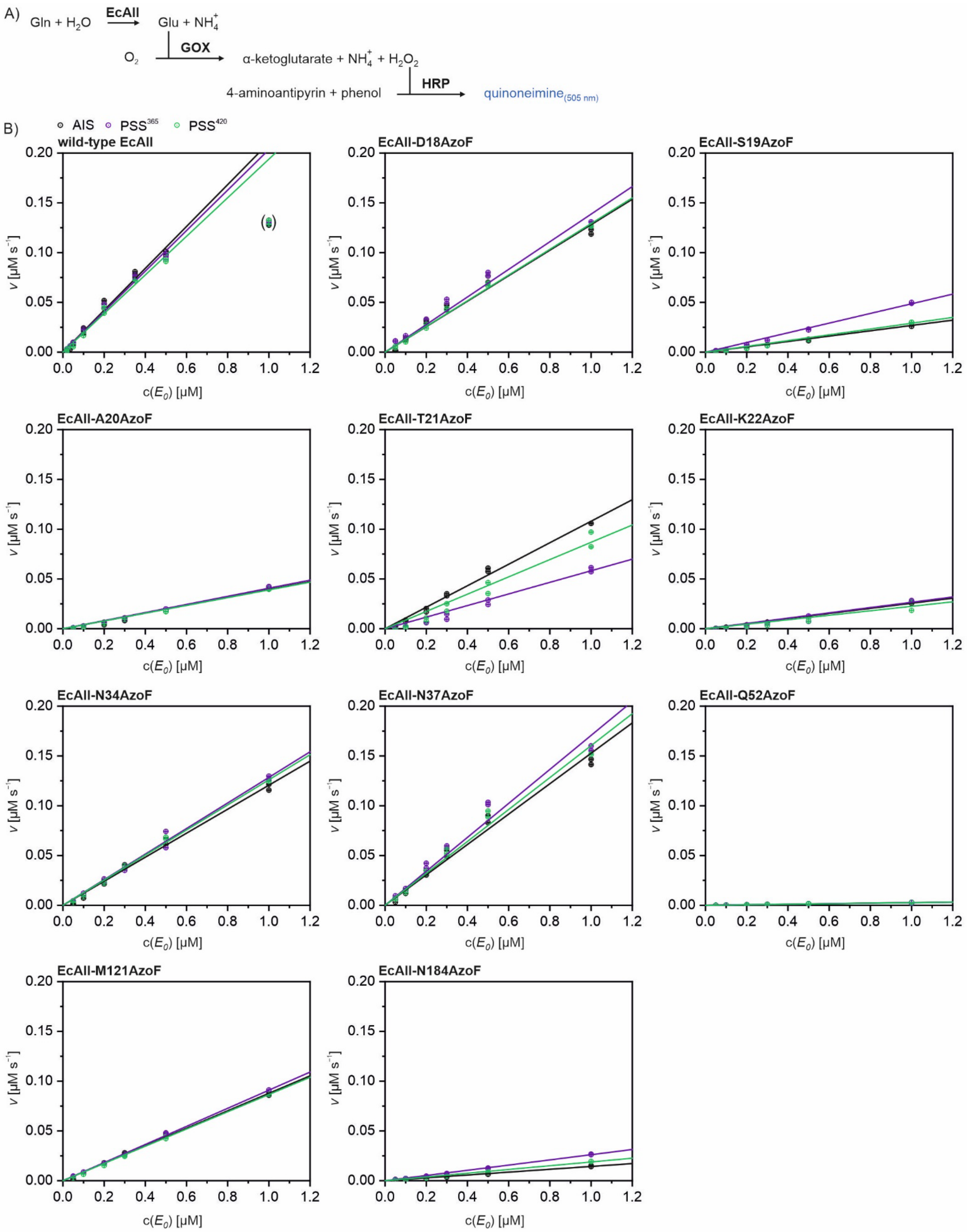

Figure S5. Screening of the glutaminase activity of EcAII-AzoF variants in their AIS, PSS^{365} and PSS^{420}. A) Glutaminase activity was determined by coupling the reaction to glutamate oxidase (GOX) and horse-radish peroxidase (HRP) following the absorbance of the produced quinoneimine at 505 nm. B) The initial catalytic rates of all EcAII variants were plotted against the used enzyme concentration to ensure that EcAII is rate-limiting in the coupled enzymatic assay. *Irradiation:* 0.2–10 µM EcAII were either kept in the dark (AIS), irradiated 2 s with 365 nm (PSS^{365}), or irradiated 2 s with 365 nm and 30 s with 420 nm

(PSS^{420}). *Reaction conditions:* 60 mM glutamine (saturated), 40 mU/mL GOX, 109 U/mL HRP, 3 mM 4-aminoantipyrine, 3 mM phenol and 0.002–1 µM EcAII in 50 mM Tris/HCl (pH 7.0) at 37°C. *Statistics:* Each circled value represents the fitted initial catalytic rate ± standard error of fit (SE); two to three technical replicates were measured for each enzyme concentration. *Note:* The activity of 1 µM WT-EcAII was smaller than expected for a linear behavior indicating that GOX and/or HRP might become rate-limiting; the values were excluded from the linear fit, respectively.

Table S2. Fitting values and statistics for the glutaminase activity screening of EcAII-AzoF variants in **Figure S5**.

EcAII variant	state	v/E_0 [s^{-1}]	R^2 (v/E_0)	LRF1 [a)] a.i. → PSS^{365}	R^2 (LRF1)	LRF2 [a)] PSS^{365} → PSS^{420}	R^2 (LRF2)
WT	a.i.	0.210 ± 0.004	0.99	1.03 ± 0.03 ↓	0.98		
	PSS^{365}	0.204 ± 0.004	0.99			1.05 ± 0.03 ↓	0.98
	PSS^{420}	0.194 ± 0.003	1.00				
D18AzoF	a.i.	0.128 ± 0.004	0.99	1.08 ± 0.05 ↑	0.96		
	PSS^{365}	0.139 ± 0.004	0.99			1.07 ± 0.03 ↓	0.98
	PSS^{420}	0.129 ± 0.002	1.00				
S19AzoF	a.i.	0.027 ± 0.001	0.99	1.81 ± 0.07 ↑	0.99		
	PSS^{365}	0.049 ± 0.001	1.00			1.67 ± 0.05 ↓	0.99
	PSS^{420}	0.029 ± 0.001	0.99				
A20AzoF	a.i.	0.039 ± 0.001	0.99	1.03 ± 0.04 ↑	0.98		
	PSS^{365}	0.041 ± 0.001	0.99			1.04 ± 0.04 ↓	0.99
	PSS^{420}	0.039 ± 0.001	0.99				
T21AzoF	a.i.	0.108 ± 0.002	1.00	1.85 ± 0.06 ↓	0.99		
	PSS^{365}	0.058 ± 0.001	0.99			1.49 ± 0.07 ↑	0.97
	PSS^{420}	0.087 ± 0.003	0.99				
K22AzoF	a.i.	0.026 ± 0.001	0.99	1.04 ± 0.05 ↑	0.97		
	PSS^{365}	0.027 ± 0.001	0.98			1.18 ± 0.09 ↓	0.94
	PSS^{420}	0.023 ± 0.001	0.96				
N34AzoF	a.i.	0.121 ± 0.002	1.00	1.06 ± 0.02 ↑	0.99		
	PSS^{365}	0.128 ± 0.002	1.00			1.02 ± 0.02 ↓	0.99
	PSS^{420}	0.126 ± 0.001	1.00				
N37AzoF	a.i.	0.153 ± 0.004	0.99	1.12 ± 0.05 ↑	0.95		
	PSS^{365}	0.171 ± 0.006	0.98			1.06 ± 0.05 ↓	0.95
	PSS^{420}	0.160 ± 0.004	0.99				
Q52AzoF	a.i.	0.003 ± 0.000	0.99	1.05 ± 0.06 ↓	0.96		
	PSS^{365}	0.003 ± 0.000	0.97			1.03 ± 0.07 ↑	0.95
	PSS^{420}	0.003 ± 0.000	0.97				
M121AzoF	a.i.	0.088 ± 0.001	1.00	1.04 ± 0.01 ↑	1.00		
	PSS^{365}	0.091 ± 0.001	1.00			1.05 ± 0.01 ↓	1.00
	PSS^{420}	0.087 ± 0.001	1.00				
N184AzoF	a.i.	0.014 ± 0.000	1.00	1.82 ± 0.05 ↑	1.00		
	PSS^{365}	0.026 ± 0.000	1.00			1.39 ± 0.03 ↓	0.99
	PSS^{420}	0.019 ± 0.000	1.00				

[a)]The activity change is indicated by an upwards (activity decrease) or downwards (activity decrease) arrow. Statistics: v/E_0 and LRF values are given as fitting value ± SE; R^2 represents the corrected R^2 as determined in Origin 2024.

Kinetic behavior of EcAII-S19AzoF and EcAII-T21AzoF

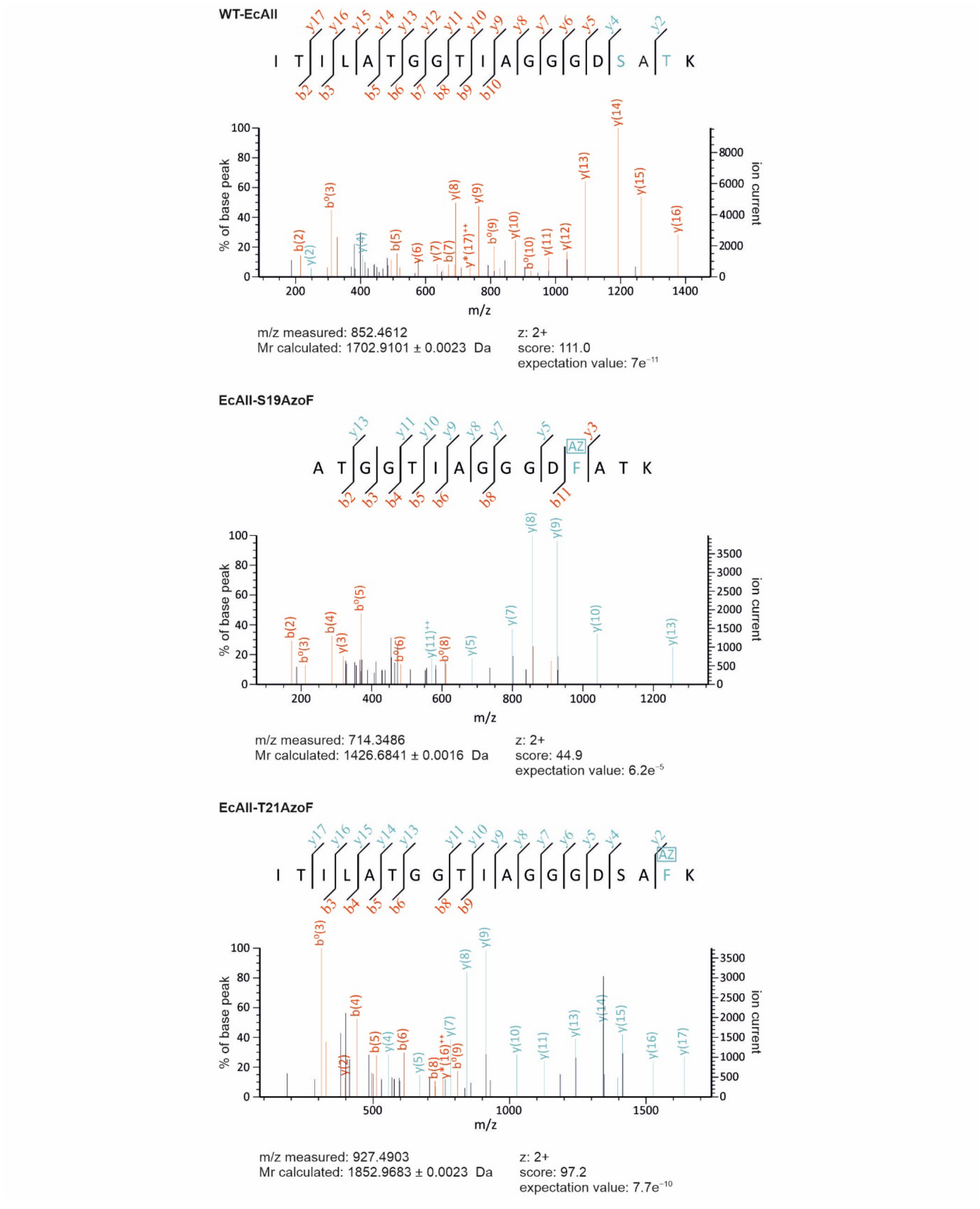

Figure S6. Confirmed identity of WT-EcAII, EcAII-S19AzoF and EcAII-T21AzoF. The fragment spectra of the peptides after tryptic digest and analysis via liquid mass spectrometry coupled to mass spectrometry (LC-ESI-MS/MS) is shown. Above the fragment spectrum, the semi-tryptic peptide harboring the two residue positions 19 and 21 (S, T, F highlighted in blue) gives an overview of all measured fragment ions; the y-fragment ions that confirm the incorporation of serine (S), threonine (T) or AzoF [identified as phenylalanine (F) with an azobenzene modification (Az)] are also highlighted in blue. All other fragment ions are colored red.

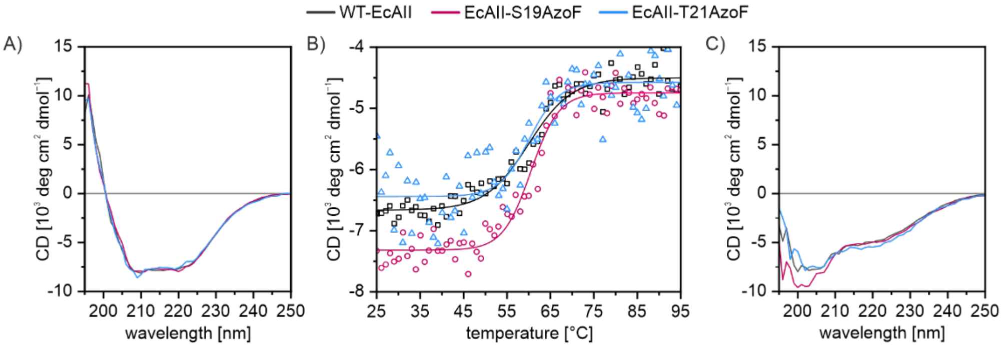

Figure S7. Circular dichroism (CD) analysis of WT-EcAII, EcAII-S19AzoF and EcAII-T21AzoF. A) Far-UV CD spectra of 8.2–10 µM EcAII proteins in 10 mM potassium phosphate buffer (pH 8.0) at room temperature indicate an intact overall fold of all three EcAII variants. B) CD thermal stability measurements of the same samples recorded at 210 nm provide denaturation midpoints of 60 °C (WT-EcAII and EcAII-T21AzoF) and 61 °C (EcAII-S19AzoF). C) Far-UV CD spectra of the same samples at 95 °C indicate that all three EcAII proteins retain some secondary structure elements indicating that EcAII might possess a second denaturation midpoint above 95°C.

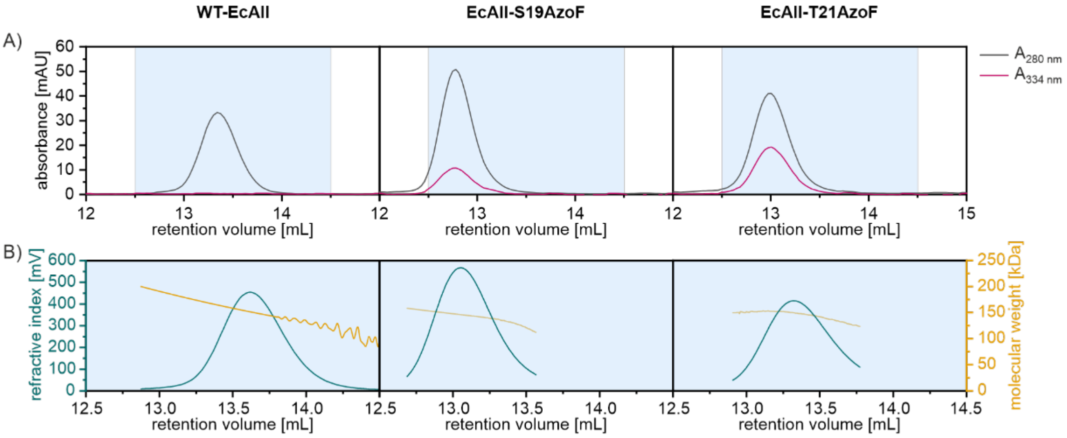

Figure S8. Tertiary structure and molecular weight analysis of WT-EcAII, EcAII-S19AzoF and EcAII-T21AzoF. A) Analytical size-exclusion chromatography (Superdex S200 column) of 40 µM EcAII proteins in 50 mM Tris/HCl (pH 7.0), 100 mM NaCl showing only one distinct signal visible at 280 nm (protein peak) and 334 nm (AzoF peak). B) Static light scattering signals of the peaks observed in (A) confirm that all three EcAII proteins consist as tetramer with mean molecular weights ± standard error of mean (SEM) of 141.8 ± 0.7 kDa (WT-EcAII; calculated: 146 kDa), 141.9 ± 0.4 kDa (EcAII-S19AzoF; calculated: 147 kDa) and 144.4 ± 0.3 kDa (EcAII-T21AzoF; calculated: 147 kDa).

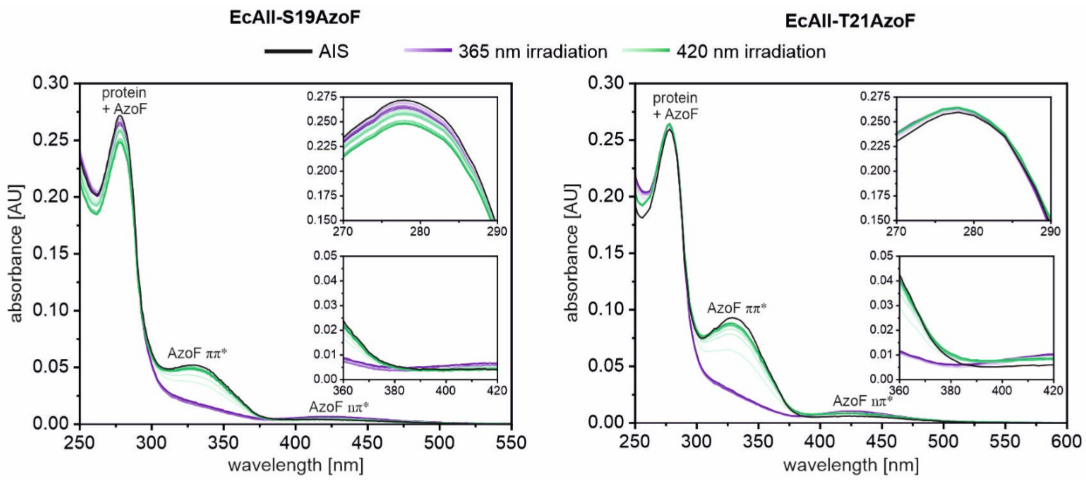

Figure S9. UV/Vis analysis of 15 µM EcAII-S19AzoF and EcAII-T21AzoF in 10 mM Tris/HCl (pH 7.0). The AIS was stepwise irradiated with 365 nm and spectra were acquired after 1 s, 2 s, 3 s, 4 s, 5 s and 20 s total irradiation time. Then the established PSS[365] was again stepwise irradiated with 420 nm and spectra were acquired after 1 s, 2 s, 3 s, 4 s, 5 s, 6 s, 7 s, 8 s, 20 s and 30 s total irradiation time. The insets show the regions where isosbestic points of AzoF isomerization should be. Between 270 nm and 290 nm, the UV/Vis signal decreases consistently instead of forming an isosbestic point, which indicates the presence of another reaction, e.g. the photocleavage of disulfide bonds. Between 360 nm and 420 nm, an unsharp isosbestic point is visible at ~382 nm and ~398 nm for EcAII-S19AzoF and EcAII-T21AzoF, respectively.

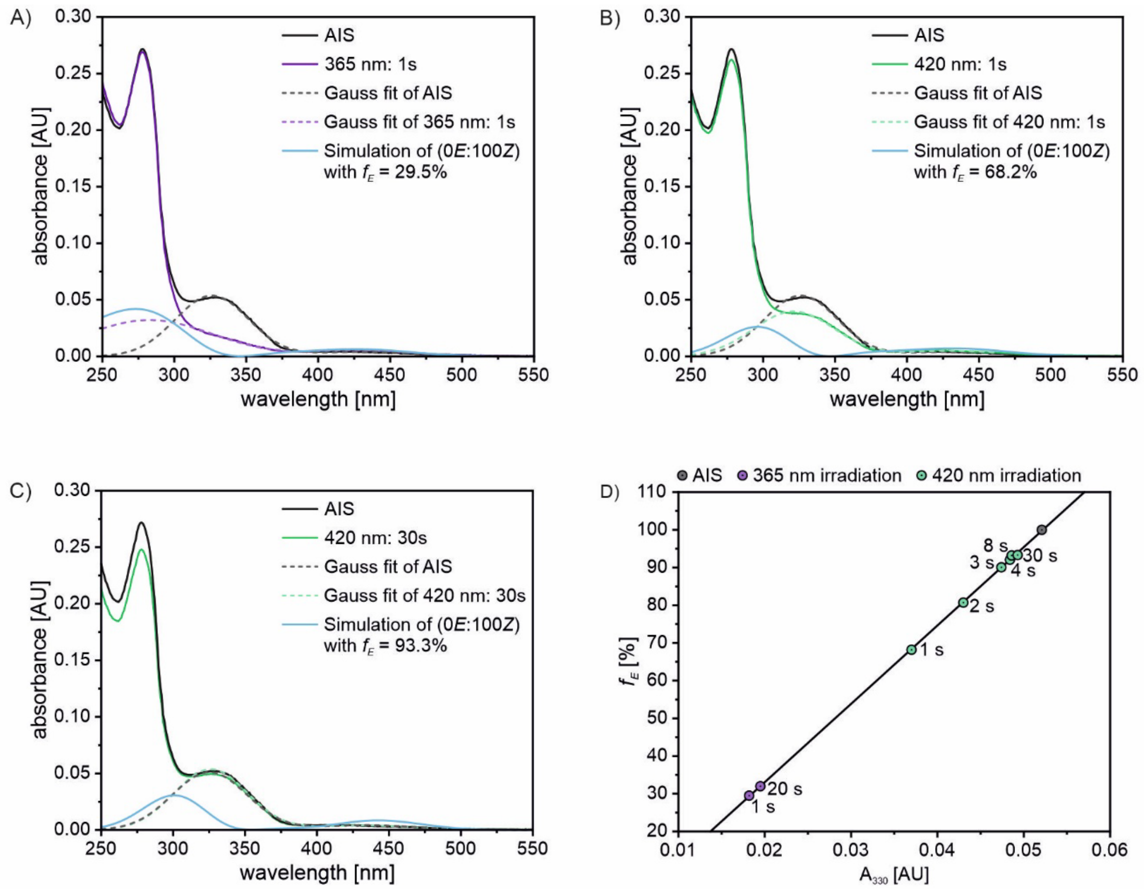

Figure S10. Determination of the *E*:*Z* distribution using peak deconvolution with Gaussian functions shown exemplarily for EcAII-S19AzoF spectra from **Figure S9**. A–C) UV/Vis spectra of 15 µM EcAII-S19AzoF in 10 mM Tris/HCl (pH 7.0) in its AIS and after 1 s of 365 nm (A), 1 s of 420 nm (B) or 30 s of 420 nm (C) irradiation (solid lines). The ππ* transition and nπ* transitions of AzoF were fitted with the multiple peak fitting tool in Origin 2024 using a Gaussian function, which yielded a cumulative fit ("Gauss fit"; dashed lines). To estimate the *E*:*Z* distribution at each timepoint of irradiation, the 0*E*:100*Z* spectrum was simulated using Equation 14, in which A_i corresponds to the Gauss fits of the spectra after irradiation, A_E to Gauss fit of the as-isolated spectrum and f_E to the fraction of the *E* isomer. f_E was obtained by manual adjustment of the simulation until the signal of the ππ* absorbance band approximates zero. The obtained f_E values are provided in each subpanel. Note: This approach is based on the assumption that the thermally equilibrated AIS contains 100% *E* isomer. D) The obtained f_E values were plotted against the measured absorbance at 330 nm and fitted with a linear regression model. The resulting linear equation was then used to generate a second y-axis showing the corresponding f_E values for all absorbance values in **Figure S11** and to calculate the *E*:*Z* distributions at the PSSs (PSD) from the exponential fits in **Figure S11**. The same procedure was repeated for EcAII-T21AzoF.

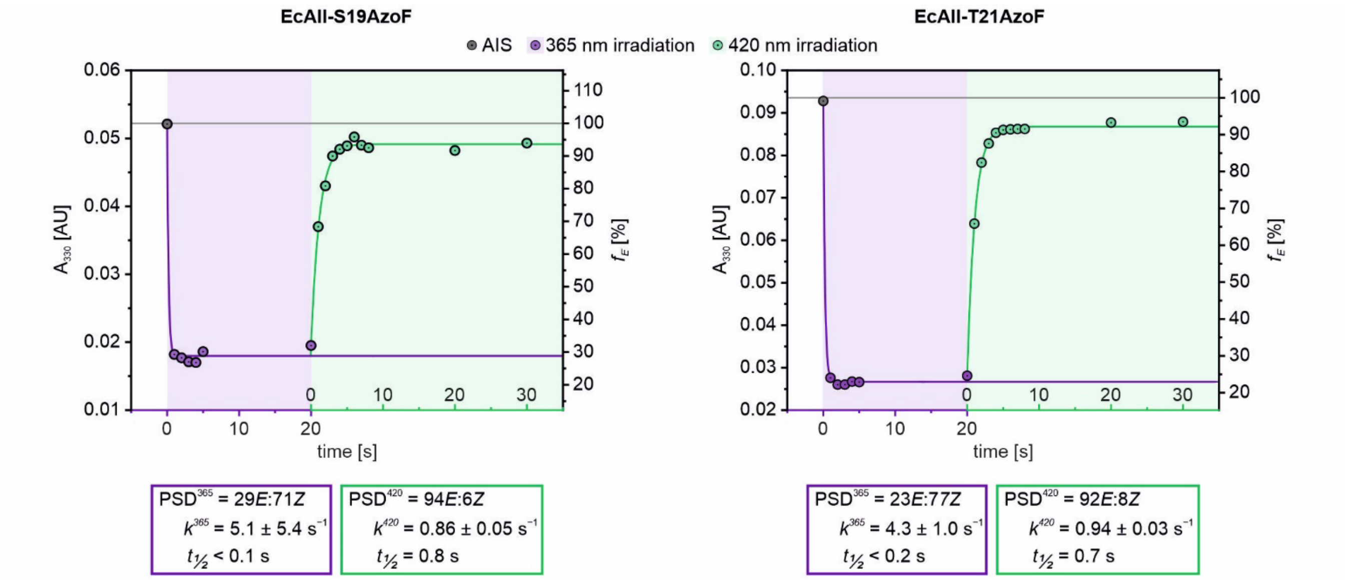

Figure S11. Progress curves following the timely change of the ππ* signal of AzoF at 330 nm from the UV/Vis analysis of EcAII-S19AzoF and EcAII-T21AzoF (cf. **Figure S9**). The signal decrease upon 365 nm irradiation as well as the signal increase upon 420 nm irradiation were fitted with mono-exponential equations. The plateau values of these fits were converted into f_E values using the equation obtained from the determination of *E*:*Z* distributions (cf. **Figure S10**), which define the PSD^{365} and PSD^{420}, respectively. The fit also derived the rate constants k^{365} and k^{420} (± SE) for the establishment of the PSS^{365} and the PSS^{420}, respectively, from which the half-times of isomerization $t_{½}$ were calculated.

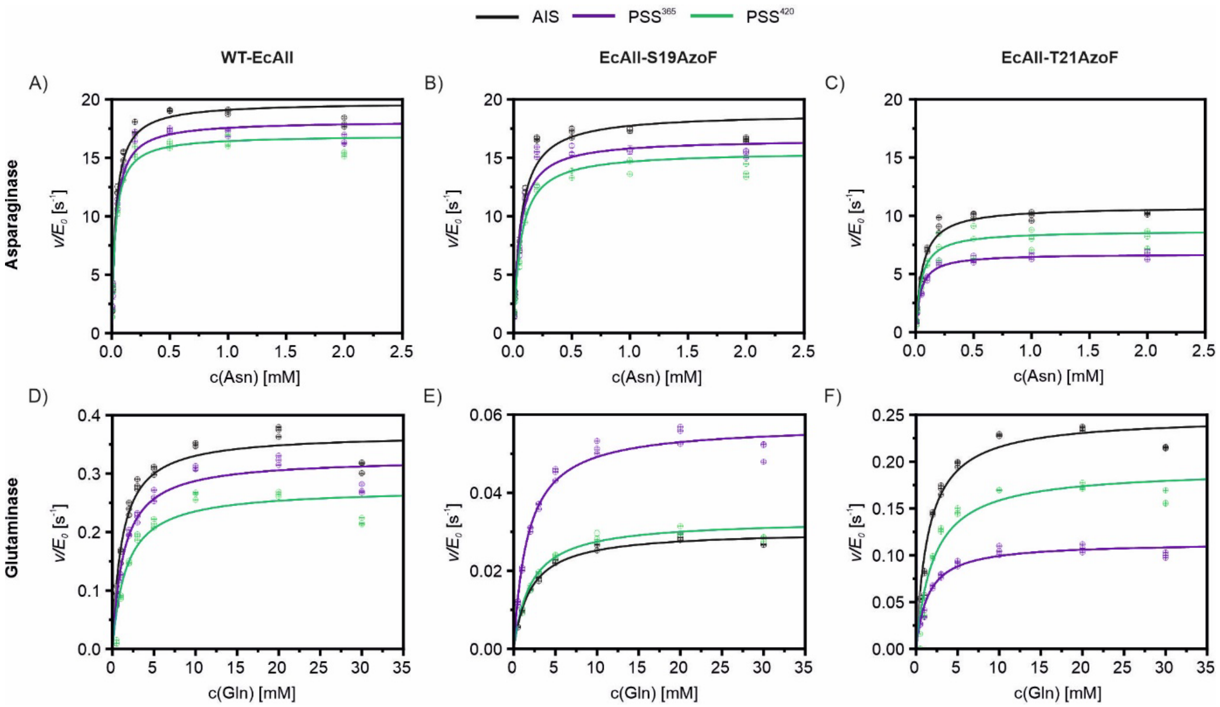

Figure S12. Michaelis-Menten curves for the asparaginase and glutaminase activity of WT-EcAII (A,D) EcAII-S19AzoF (B,E) and EcAII-T21AzoF (C,F). Steady-state kinetics were measured for all enzymes in their AIS, their PSS^{365}, and their PSS^{420}. *Irradiation (asparaginase):* 0.2 µM (WT-EcAII and EcAII-S19AzoF) or 0.4 µM (EcAII-T21AzoF) protein were either kept in the dark (AIS), irradiated 2 s with 365 nm (PSS^{365}), or irradiated 2 s with 365 nm and 30 s with 420 nm (PSS^{420}). *Reaction conditions (asparaginase):* 0.005–2 mM asparagine, 0.25 mM NADH, 5 mM α-ketoglutarate, 14 U/mL GDH and 0.01/0.02 µM EcAII (20-fold dilution) in 10 mM Tris/HCl (pH 7.0) at 37°C. *Irradiation (glutaminase):* 1 µM (WT-EcAII) or 3 µM (EcAII-S19AzoF and EcAII-T21AzoF) protein were either kept in the dark (AIS), irradiated 2 s with 365 nm (PSS^{365}), or irradiated 2 s with 365 nm and 30 s with 420 nm (PSS^{420}). *Reaction conditions (glutaminase):* 0.5–30 mM glutamine, 40 mU/mL GOX, 109 U/mL HRP, 3 mM 4-aminoantipyrine, and 3 mM phenol and 0.1/0.3 µM EcAII (10-fold dilution) in 10 mM Tris/HCl (pH 7.0) at 37°C. A–F) *Statistics:* Each circled value represents the fitted initial catalytic rate v/E_0 ± SE; three technical replicates were measured for each substrate concentration.

Table S3. Fitting values and statistics for the asparaginase steady-state kinetics of WT-EcAII, EcAII-S19AzoF and EcAII-T21AzoF in **Figure S12**.

**EcAII variant**	**state**	**k_{cat} [s^{-1}]**	**R^2**	**LRF1[a)] a.i. → PSS^{365}**	**R^2 (LRF1)**	**LRF2[a)] PSS^{365} → PSS^{420}**	**R^2 (LRF2)**
WT	a.i.	19.7 ± 0.3	0.99	1.09 ± 0.03 ↓	0.99		
	PSS^{365}	18.2 ± 0.3	0.99			1.07 ± 0.03 ↓	0.99
	PSS^{420}	16.9 ± 0.3	0.98				
S19AzoF	a.i.	18.8 ± 0.5	0.97	1.13 ± 0.04 ↓	0.97		
	PSS^{365}	16.6 ± 0.3	0.98			1.07 ± 0.05 ↓	0.98
	PSS^{420}	15.5 ± 0.5	0.97				
T21AzoF	a.i.	10.8 ± 0.2	0.98	1.60 ± 0.07 ↓	0.98		
	PSS^{365}	6.7 ± 0.2	0.98			1.29 ± 0.08 ↑	0.95
	PSS^{420}	8.7 ± 0.4	0.95				
**EcAII variant**	**state**	**K_m [mM]**	**R^2**	**LRF1[a)] a.i. → PSS^{365}**	**R^2 (LRF1)**	**LRF2[a)] PSS^{365} → PSS^{420}**	**R^2 (LRF2)**
WT	a.i.	0.034 ± 0.002	0.99	1.00 ± 0.09	0.99		
	PSS^{365}	0.034 ± 0.002	0.99			1.10 ± 0.11 ↓	0.99
	PSS^{420}	0.031 ± 0.002	0.98				
S19AzoF	a.i.	0.060 ± 0.006	0.97	1.18 ± 0.16 ↓	0.97		
	PSS^{365}	0.051 ± 0.005	0.98			1.18 ± 0.18 ↑	0.98
	PSS^{420}	0.060 ± 0.006	0.97				
T21AzoF	a.i.	0.057 ± 0.004	0.98	1.35 ± 0.21 ↓	0.98		
	PSS^{365}	0.042 ± 0.004	0.98			1.13 ± 0.24 ↑	0.95
	PSS^{420}	0.047 ± 0.007	0.95				
**EcAII variant**	**state**	**k_{cat}/K_m [10^3 s^{-1} M^{-1}]**	**R^2**	**LRF1[a)] a.i. → PSS^{365}**	**R^2 (LRF1)**	**LRF2[a)] PSS^{365} → PSS^{420}**	**R^2 (LRF2)**
WT	a.i.	582.3 ± 31.3	0.99	1.09 ± 0.09 ↓	0.99		
	PSS^{365}	534.9 ± 30.2	0.99			1.03 ± 0.08 ↑	0.99
	PSS^{420}	550.5 ± 30.3	0.98				
S19AzoF	a.i.	311.7 ± 23.8	0.97	1.04 ± 0.12 ↑	0.97		
	PSS^{365}	325.6 ± 26.5	0.98			1.26 ± 0.16 ↓	0.98
	PSS^{420}	258.8 ± 21.6	0.97				
T21AzoF	a.i.	190.6 ± 12.3	0.98	1.18 ± 0.15 ↓	0.98		
	PSS^{365}	161.2 ± 11.8	0.98			1.14 ± 0.20 ↑	0.95
	PSS^{420}	184.2 ± 19.5	0.95				

[a)]The activity change is indicated by an upwards (activity decrease) or downwards (activity decrease) arrow. Statistics: k_{cat}, k_{cat}/K_m, K_m and LRF values are given as fitting value ± SE; R^2 represents the corrected R^2 as determined in Origin 2024.

Table S4. Fitting values and statistics for the glutaminase steady-state kinetics of WT-EcAII, EcAII-S19AzoF and EcAII-T21AzoF in **Figure S12**.

EcAII variant	state	k_{cat} [s^{-1}]	R^2	LRF1[a)] a.i. → PSS^{365}	R^2 (LRF1)	LRF2 [a)] PSS^{365} → PSS^{420}	R^2 (LRF2)
WT	a.i.	0.368 ± 0.009	0.91	1.13 ± 0.04 ↓	0.91		
	PSS^{365}	0.326 ± 0.009	0.91			1.19 ± 0.06 ↓	0.90
	PSS^{420}	0.274 ± 0.011	0.87				
S19AzoF	a.i.	0.030 ± 0.000	0.98	1.90 ± 0.07 ↑	0.98		
	PSS^{365}	0.057 ± 0.001	0.97			1.74 ± 0.06 ↓	0.97
	PSS^{420}	0.033 ± 0.001	0.94				
T21AzoF	a.i.	0.248 ± 0.006	0.94	2.18 ± 0.11 ↓	0.97		
	PSS^{365}	0.114 ± 0.002	0.95			1.69 ± 0.11 ↑	0.92
	PSS^{420}	0.192 ± 0.009	0.90				

EcAII variant	state	K_m [mM]	R^2	LRF1[a)] a.i. → PSS^{365}	R^2 (LRF1)	LRF2 [a)] PSS^{365} → PSS^{420}	R^2 (LRF2)
WT	a.i.	1.14 ± 0.13	0.91	1.19 ± 0.21 ↑	0.91		
	PSS^{365}	1.35 ± 0.17	0.91			1.23 ± 0.27 ↑	0.90
	PSS^{420}	1.66 ± 0.30	0.87				
S19AzoF	a.i.	1.99 ± 0.12	0.98	1.18 ± 0.18 ↓	0.98		
	PSS^{365}	1.68 ± 0.14	0.97			1.21 ± 0.19 ↑	0.97
	PSS^{420}	2.03 ± 0.24	0.94				
T21AzoF	a.i.	1.50 ± 0.16	0.94	1.04 ± 0.24 ↓	0.97		
	PSS^{365}	1.44 ± 0.14	0.95			1.47 ± 0.43 ↑	0.92
	PSS^{420}	2.12 ± 0.38	0.90				

EcAII variant	state	k_{cat}/K_m [10^3 s^{-1} M^{-1}]	R^2	LRF1[a)] a.i. → PSS^{365}	R^2 (LRF1)	LRF2 [a)] PSS^{365} → PSS^{420}	R^2 (LRF2)
WT	a.i.	0.323 ± 0.032	0.91	1.34 ± 0.20 ↓	0.91		
	PSS^{365}	0.241 ± 0.025	0.91			1.46 ± 0.27 ↓	0.90
	PSS^{420}	0.165 ± 0.025	0.87				
S19AzoF	a.i.	0.015 ± 0.001	0.98	2.25 ± 0.28 ↑	0.98		
	PSS^{365}	0.034 ± 0.002	0.97			2.10 ± 0.27 ↓	0.97
	PSS^{420}	0.016 ± 0.002	0.94				
T21AzoF	a.i.	0.165 ± 0.015	0.94	2.10 ± 0.40 ↓	0.97		
	PSS^{365}	0.079 ± 0.007	0.95			1.15 ± 0.28 ↑	0.92
	PSS^{420}	0.091 ± 0.013	0.90				

[a)]The activity change is indicated by an upwards (activity decrease) or downwards (activity decrease) arrow. Statistics: k_{cat}, k_{cat}/K_m, K_m and LRF values are given as fitting value ± SE; R^2 represents the corrected R^2 as determined in Origin 2024.

Extended Text S1. Optimization of the length of irradiation during reaction measurements

We tested whether the auxiliary enzymes of both coupled assays were affected by irradiation (**Figure S13A**). Both FAD and heme cofactors of GOX and HRP absorb 365 nm light similarly strong or even stronger than 420 nm light.[2,3] Hence, we suspected that irradiation with 250 mW cm^{-2} of 365 nm might lead to similar or more serious damage of the auxiliary enzymes, owing to the higher intensity of irradiation, than 60 mW cm^{-2} of 420 nm. We irradiated the master mix containing all substrates, co-substrates and auxiliary enzymes for 30 s, 60 s or 90 s with 365 nm and then initiated the reaction with WT-EcAII. By this, we found that the asparaginase activity of WT-EcAII was largely unaffected indicating that GDH did not become rate-limiting. In comparison, WT-EcAII retained its glutaminase activity after 30 s irradiation but slowed down to ~30% of its initial activity after 90 s irradiation confirming that GOX and/or HRP were light-sensitive and became rate-limiting. Thus, we chose to irradiate less than 30 s with 365 nm and 420 nm in total in our subsequent assays.

We further aimed to reduce irradiation times to minimize the harmful photoinduced cleavage of the disulfide bond in EcAII. For this, we chose to irradiate with 365 nm for 1 s, 2 s, 3 s, 4 s, 5 s, 10 s, 15 s, 20 s or 30 s during the measurement and, hence, in the presence of EcAII (**Figure S13B–E**). While 1–10 s irradiation caused only a slight deactivation of both asparaginase and glutaminase activity of WT-EcAII with LRFs <1.5, 15–30 s irradiation led to a significant decrease specifically in the glutaminase reaction with LRFs >1.5. Similarly, the LRFs of both photoxenases were reduced after 15–30 s irradiation compared to 1–5 s irradiation. Thus, we decided to irradiate our direct photocontrol samples for <10 s with 250 mW cm^{-2} of 365 nm and up to 30 s with 60 mW cm^{-2} of 420 nm, of which we expected less photodamage. Moreover, we chose irradiation durations at which PSS formation was at least 99% completed defined by 6.69×$t_{1/2}$[4], i.e. 1.3 s with 365 nm (where $t_{1/2}$ = 0.2 s as determined in **Figure S11**) and 5.3 s with 420 nm (where $t_{1/2}$ = 0.8 s as determined in **Figure S11**).

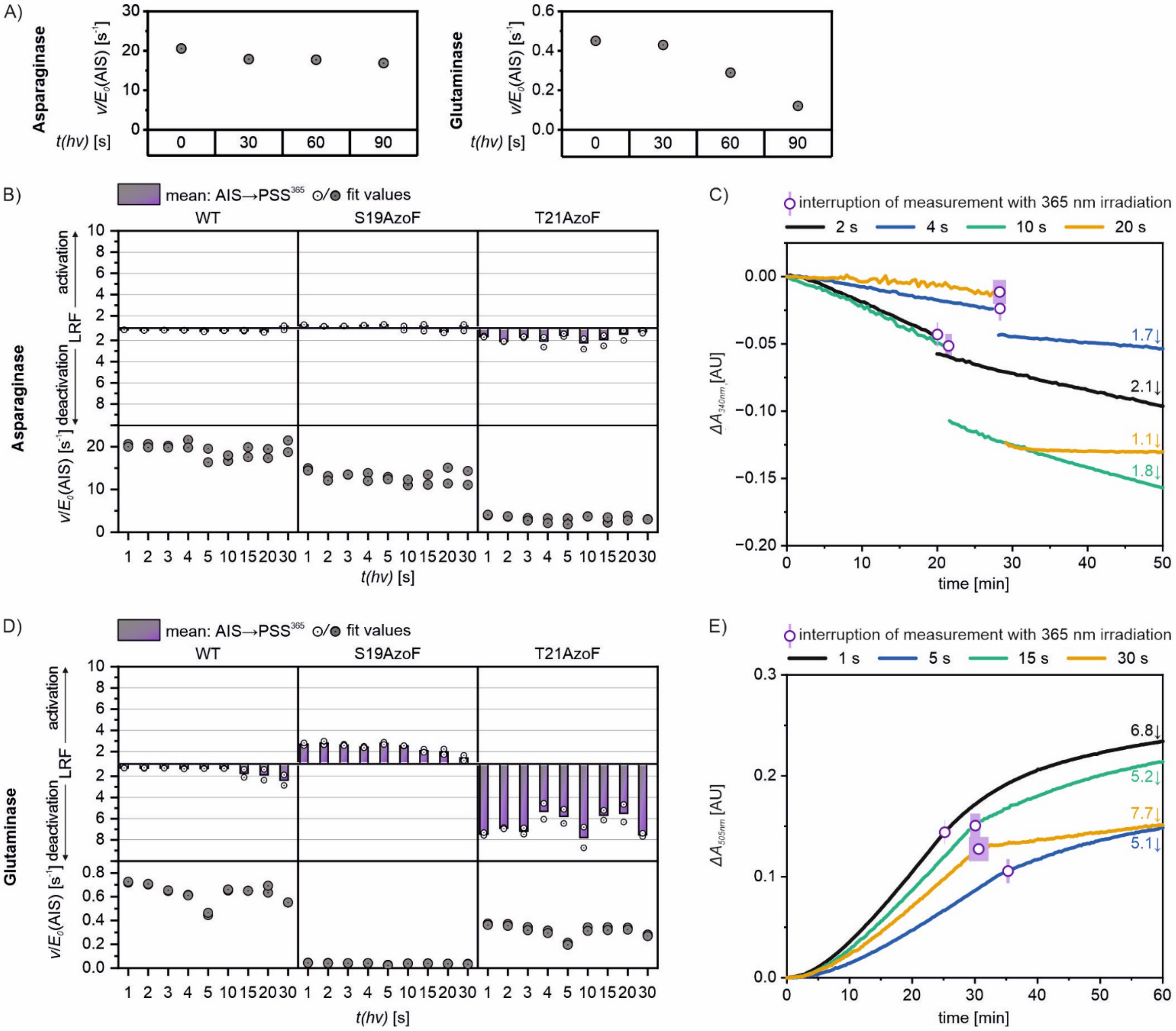

Figure S13. Optimization of the irradiation length for activity measurements of WT-EcAII, EcAII-S19AzoF and EcAII-T21AzoF. A) Asparaginase and glutaminase assays were irradiated for certain time periods *t*(*hv*) before the addition of WT-EcAII in its AIS. B–E) Asparaginase (B, C) and glutaminase (D, E) activities determined via 365 nm irradiation for various *t*(*hv*) during the turnover measurement. B, D) Summary of the obtained LRFs (top panels) and as control the v/E_0 activity values of the as-isolated variants (bottom panels), which indicate slight fluctuations in EcAII concentration. C, E) Exemplary baseline corrected raw data for EcAII-T21AzoF. The drop of absorbance after irradiating the asparaginase reaction is caused by a photoinduced degradation of NADH. *Irradiation:* Measurements were stopped after ≥25 min (full circles in C and E) and the reactions were irradiated for various time periods with 365 nm to establish the PSS^{365}. *Reaction conditions (asparaginase):* 6 mM asparagine, 14 U/mL GDH, 0.25 mM NADH, 5 mM α-ketoglutarate, and 2/3/4 nM WT-EcAII/EcAII-S19AzoF/EcAII-T21AzoF in 50 mM Tris/HCl (pH 7.0) at 37°C. *Reaction conditions (glutaminase):* 60 mM glutamine, 109 U/mL HRP, 80 mU/mL GOX, 3 mM 4-aminoantipyrine, 3 mM phenol, and 0.1/0.5/0.2 µM WT-EcAII/EcAII-S19AzoF/EcAII-T21AzoF in 10 mM Tris/HCl (pH 7.0) at 37°C. *Statistics:* Activity and LRF values were obtained by a linear fit of the different segments of the progress curve and are plotted as circles without the SE for two technical replicates. Mean LRF values are indicated by bars.

Extended Text S2. Photocontrol efficiencies upon irradiation of apo and holo EcAII.

To compare the photocontrol efficiencies obtained after irradiation of the apo or holo EcAII variants, we applied substrate in saturation (>60·K_m,) because the k_{cat} of asparagine and glutamine hydrolysis showed the largest effects upon irradiation.

We initially focused on the asparaginase activity. When we initiated the reaction with EcAII either in its AIS or its irradiated apo PSS^{365} (PSS^{365}_{apo}), we observed no change in activity for WT-EcAII (LRF ~1.2) and EcAII-S19AzoF (LRF ~1.1). Consistent with our previous findings, EcAII-T21AzoF exhibited mediocre photocontrol (LRF ~1.7; **Figure S14A**). To determine the photocontrol efficiencies upon irradiation of holo EcAII, we instead initiated the reaction with EcAII in its AIS, followed asparagine turnover until the steady state was reached for several minutes, paused the measurement to irradiate with 365 nm establishing PSS^{365}_{holo} and subsequently monitored the reaction until the new steady state was established for several minutes (**Figure S14B**).[5] This time, all three reactions were only minimally affected by irradiation [WT-EcAII: LRF ~1.1, EcAII-S19AzoF: LRF ~1.2, EcAII-T21AzoF: LRF ~1.1].

Next, we were interested whether the LRF also changes for glutamine turnover when apo or holo EcAII is irradiated. For this, we performed the same experiment with the glutaminase reaction. As expected from previous measurements, WT-EcAII retained activity (LRF ~1.1), EcAII-S19AzoF increased activity (LRF ~2.3) and EcAII-T21AzoF reduced activity (LRF ~2.4) in its PSS^{365}_{apo} compared to its AIS (**Figure S15A**). We then initiated the reaction with EcAII in its AIS and irradiated the reaction during the measurement to produce PSS^{365}_{holo} (**Figure S15B**). As a result, WT-EcAII was again unaffected by light (LRF ~1.0), but EcAII-S19AzoF (LRF ~3.0) and EcAII-T21AzoF (LRF ~3.2) showed a higher photocontrol efficiency in the PSS^{365}_{holo} than in the PSS^{365}_{apo}.

Altogether, irradiation of holo EcAII led to lower photocontrol efficiencies for the asparaginase reaction but higher photocontrol efficiencies for the glutaminase reaction compared to irradiation of apo EcAII. To further evaluate this apparent difference in LRF, we performed a fourth experiment, in which we initiated asparagine/glutamine turnover with EcAII in the PSS^{365}_{apo} and irradiated the reaction during the measurement to produce the PSS^{365}_{holo}. We thereby expected that activity will either be maintained, if the previously observed difference was experimental happenstance caused by replicate measurements, or changed, if the previously observed difference was caused by the formation of two interconvertible conformational states in PSS^{365}_{apo} and PSS^{365}_{holo}. Remarkably, while WT-EcAII was again unaffected by light, the glutaminase activities of EcAII-S19AzoF and EcAII-T21AzoF further increased or decreased upon irradiation (**Figure S15C**) and reached similar values for PSS^{365}_{holo} as after irradiation of the holo AIS (**Figure S15B**). This initially confirmed that irradiation of apo and holo EcAII promotes the formation of two different conformational states, PSS^{365}_{apo} and PSS^{365}_{holo}, that are interconvertible. In contrast to this conclusion, the asparaginase activity of EcAII-T21AzoF was maintained after one 365 nm irradiation step (**Figure S16**). To test

whether the interconversion of the conformational states in PSS^{365}_{apo} and PSS^{365}_{holo} might be more difficult with asparagine than with glutamine, we irradiated the reaction again with 420 nm and repeated both irradiation steps (365 nm and 420 nm) twice more. By this, we hoped to force the enzyme to change its conformation through repeated isomerization of AzoF. As a result, the activity indeed approximated the activity of the "AIS → PSS^{365}_{holo}" reaction (**Figure S14B**) after the last 365 nm irradiation step. As a control, the asparaginase activity of WT-EcAII and EcAII-S19AzoF remained the same throughout the measurements (**Figure S16**).

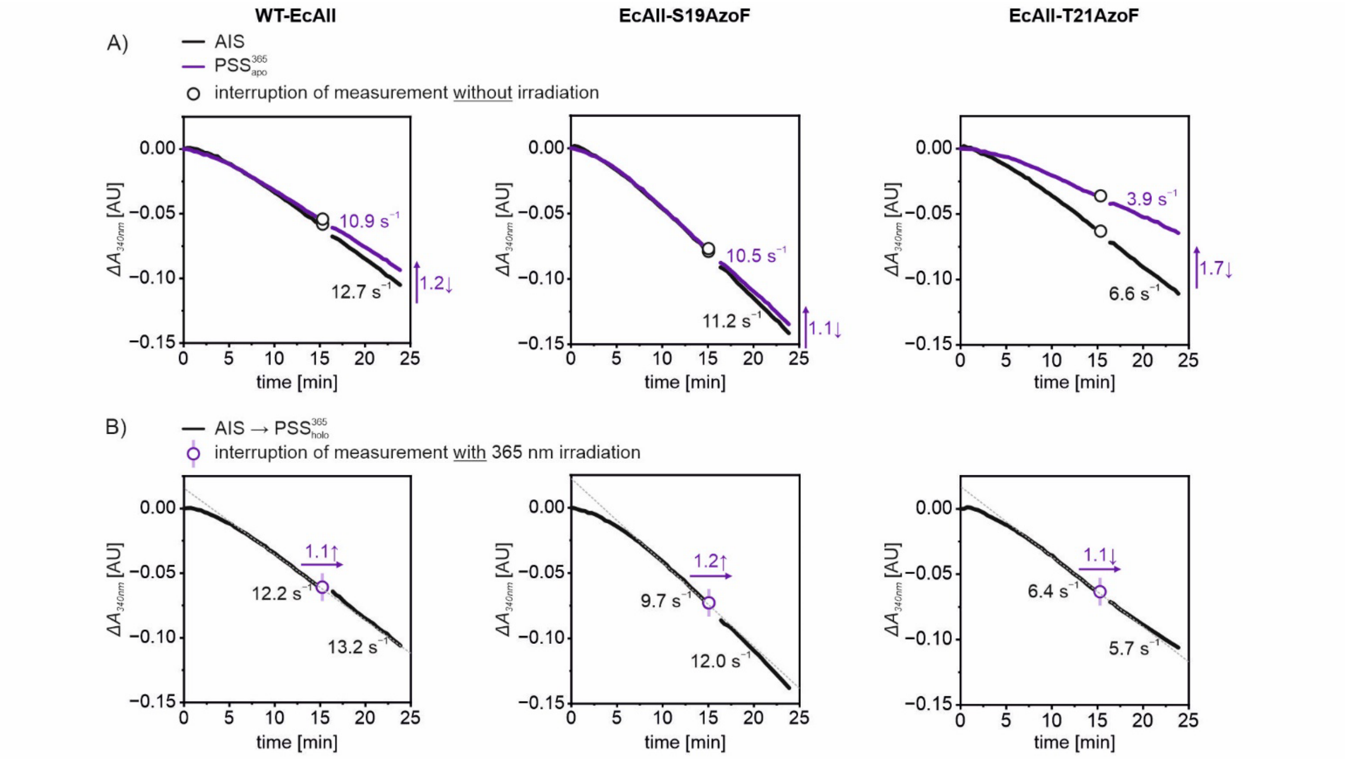

Figure S14. Comparison of photocontrol efficiencies for asparagine turnover via irradiation of apo or holo EcAII. A) The asparaginase reaction was started by the addition of either the enzyme in its non-irradiated apo AIS or in its apo state after 365 nm irradiation prior to the measurement (PSS^{365}_{apo}). B) The asparaginase reaction was started with non-irradiated AIS and irradiated during the measurement with 365 nm to establish PSS^{365}_{holo}. Grey dashed lines constitute a trendline for the linear reaction course of the first reaction phase. *Irradiation:* To generate PSS^{365}_{apo}, the as-isolated enzyme was irradiated for 10 s with 365 nm. To establish PSS^{365}_{holo}, the measurements were interrupted, and the samples were irradiated for 10 s with 365 nm. *Reaction conditions:* 5 mM asparagine (saturated), 14 U/mL GDH, 0.25 mM NADH, 5 mM α-ketoglutarate, and 2 nM WT-EcAII / 3 nM EcAII-S19AzoF / 4 nM EcAII-T21AzoF in 10 mM Tris/HCl (pH 7.0) at 37°C.

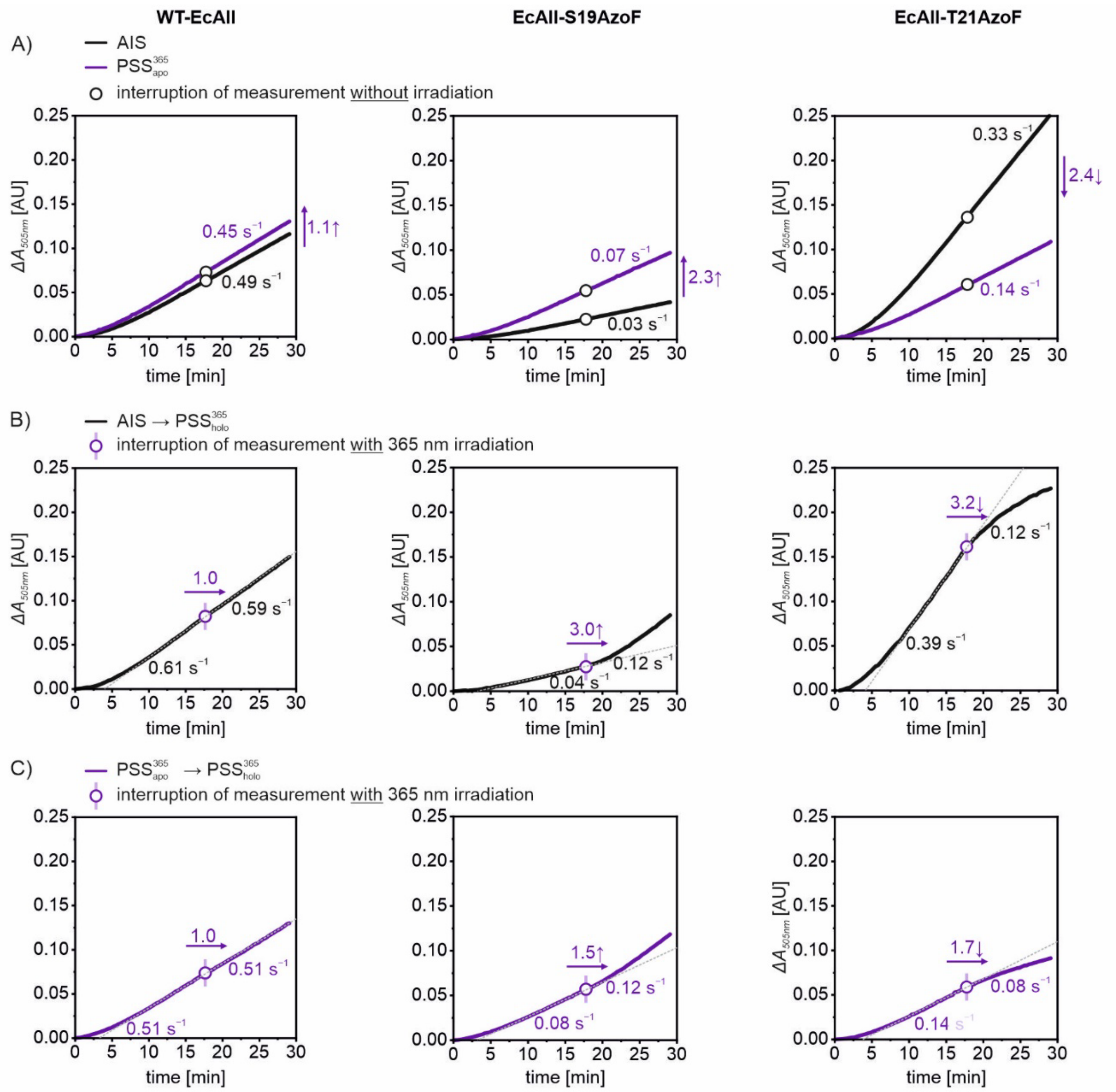

Figure S15. Comparison of photocontrol efficiencies for glutamine turnover via irradiation of apo or holo EcAII. A) The glutaminase reaction was started by the addition of either the enzyme in its non-irradiated apo AIS or in its apo state after 365 nm irradiation prior to the measurement (PSS^{365}_{apo}). B) The glutaminase reaction was started with non-irradiated AIS and irradiated during the measurement with 365 nm to establish PSS^{365}_{holo}. C) The glutaminase reaction was started with enzyme in its PSS^{365}_{apo} and irradiated during the measurement with 365 nm to establish PSS^{365}_{holo}. B,C) Grey dashed lines constitute a trendline for the linear reaction course of the first reaction phase. *Irradiation:* To generate PSS^{365}_{apo}, the as-isolated enzyme was irradiated for 10 s with 365 nm. To establish PSS^{365}_{holo}, the measurements were interrupted, and the samples were irradiated for 10 s with 365 nm. *Reaction conditions:* 60 mM glutamine, 109 U/mL HRP, 100 mU/mL GOX, 3 mM 4-aminoantipyrine, 3 mM phenol and 0.1 µM WT-EcAII / 0.5 µM EcAII-S19AzoF / 0.3 µM EcAII-T21AzoF in 10 mM Tris/HCl (pH 7.0) at 37°C.

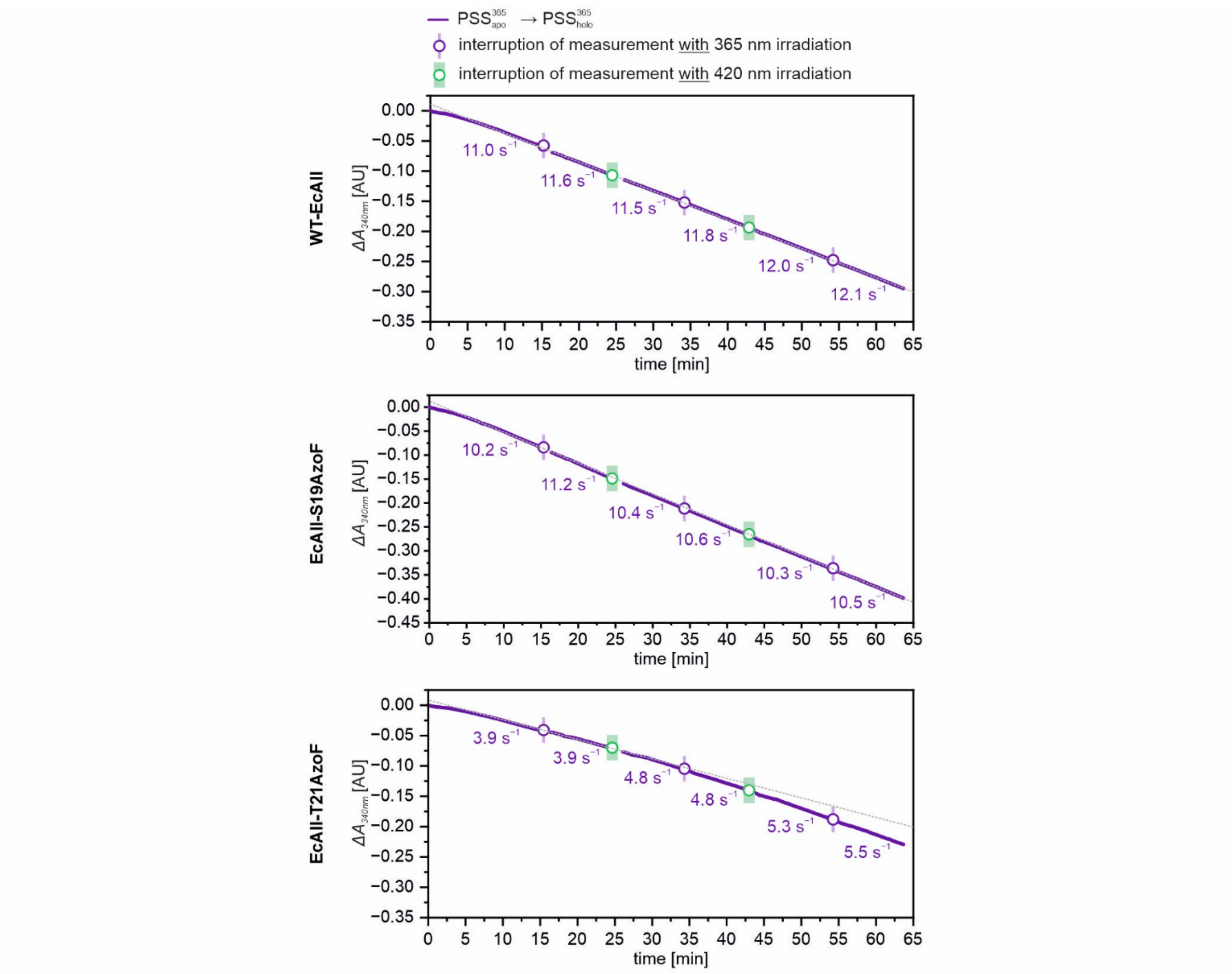

Figure S16. Progress curves for asparagine turnover with repeated 365/420 nm irradiation of WT-EcAII, EcAII-S19AzoF and EcAII-T21AzoF. The asparaginase reaction was started with enzyme in its PSS$^{365}_{apo}$ and repeatedly irradiated during the measurement with 365 nm and 420 nm to force the enzyme into the PSS$^{365}_{holo}$ conformation. *Irradiation:* The measurements were interrupted and the samples were irradiated for 10 s with 365 nm or for 30 s with 420 nm. *Reaction conditions:* 5 mM asparagine (saturated), 14 U/mL GDH, 0.25 mM NADH, 5 mM α-ketoglutarate, and 2 nM WT-EcAII / 3 nM EcAII-S19AzoF / 4 nM EcAII-T21AzoF in 10 mM Tris/HCl (pH 7.0) at 37°C.

Validation of substrate-specific photocontrol in EcAII

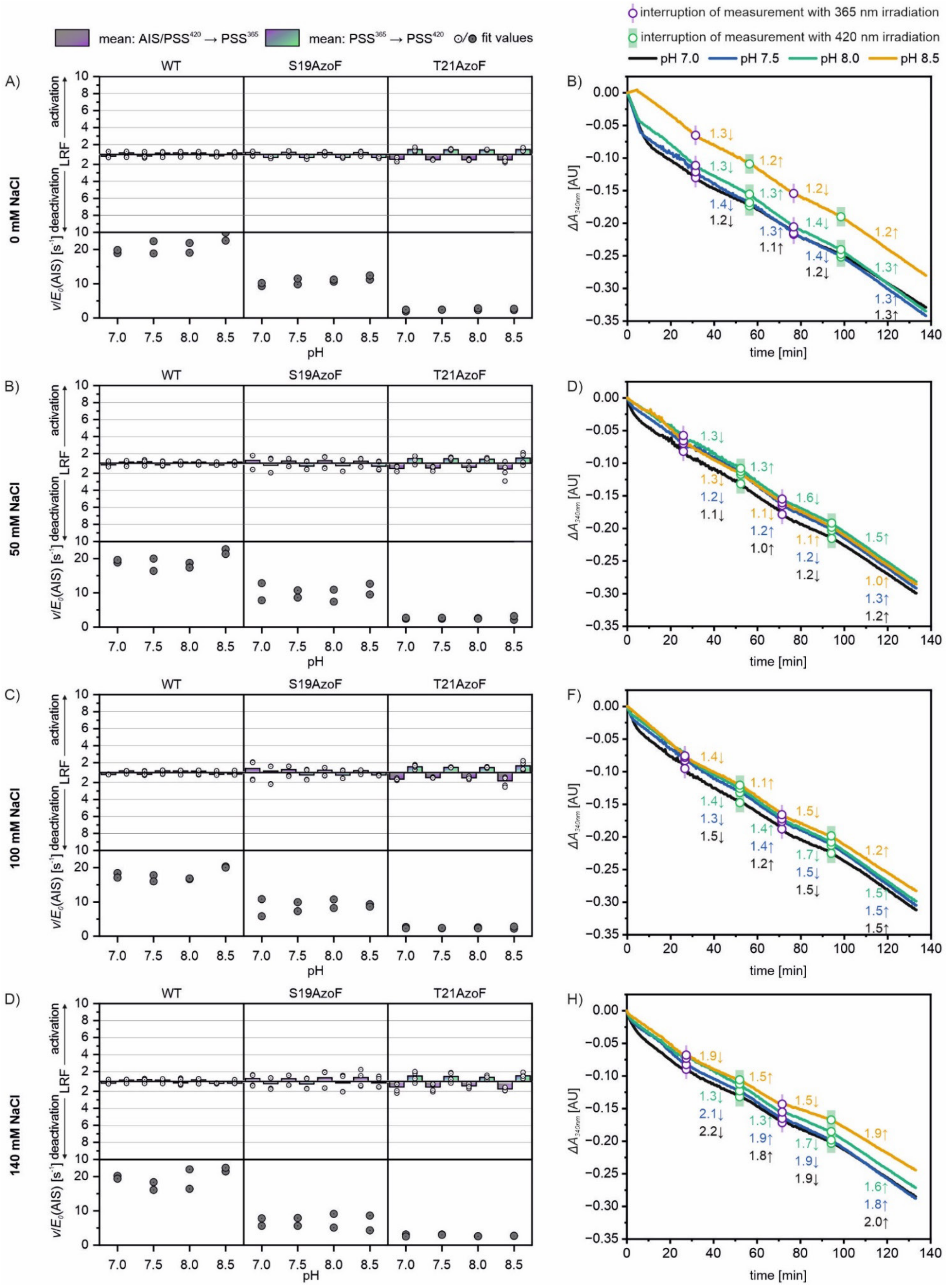

Figure S17. Asparaginase activities and LRFs at various pH for WT-EcAII, EcAII-S19AzoF and EcAII-T21AzoF determined via irradiation during the turnover measurement. A, C, E, G) Summary of the

obtained LRFs (top panels) and as control the *v/E₀* activity values of the as-isolated variants (bottom panels) for c(NaCl) of 0 mM (A), 50 mM (C), 100 mM (E) and 140 mM (G). B, D, F, H) Exemplary baseline corrected progress curves of EcAII-T21AzoF for c(NaCl) of 0 mM (B), 50 mM (D), 100 mM (F) and 140 mM (H). *Irradiation:* Measurements were stopped after ~25 min and the reactions were irradiated with 365 nm (2 s per 4 wells) or 420 nm (8 s per 4 wells) to establish the PSS^{365} or the PSS^{420}, respectively. *Reaction conditions:* 6 mM asparagine (saturated), 14 U/mL GDH, 0.25 mM NADH, 5 mM α-ketoglutarate, 0/50/100/140 mM NaCl, and 2/3/5 nM WT-EcAII/EcAII-S19AzoF/EcAII-T21AzoF in 50 mM Tris/HCl (pH 7.0/7.5/8.0/8.5) at 37°C. *Statistics:* Activity and LRF values were obtained by a linear fit of the different segments of the progress curve and are plotted as circles without the SE for two technical replicates. Mean LRF values are indicated by bars.

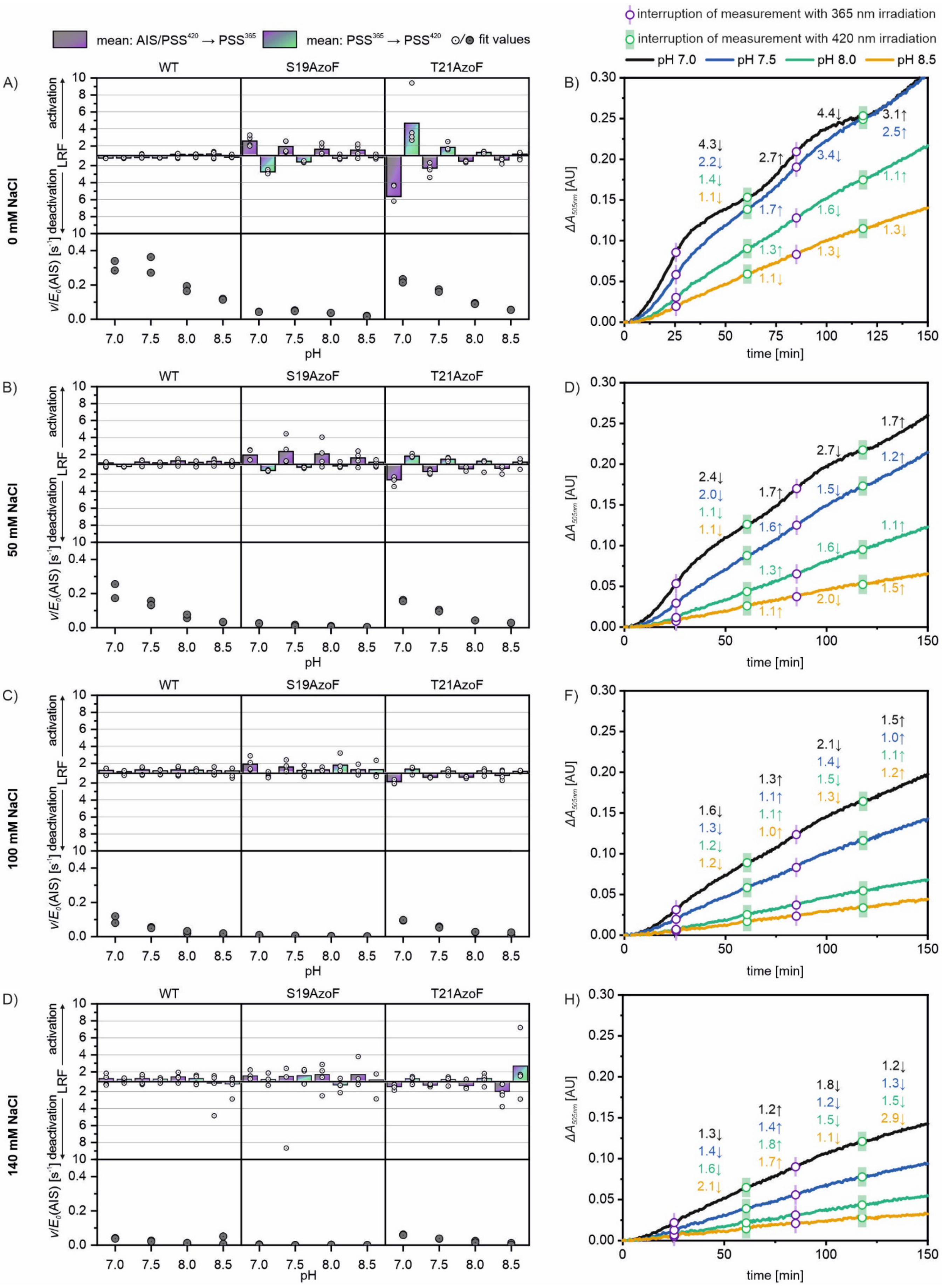

Figure S18. Glutaminase activities and LRFs at various pH for WT-EcAII, EcAII-S19AzoF and EcAII-T21AzoF determined via irradiation during the turnover measurement. A, C, E, G) Summary of the

obtained LRFs (top panels) and as control the *v/E₀* activity values of the as-isolated variants (bottom panels) for c(NaCl) of 0 mM (A), 50 mM (C), 100 mM (E) and 140 mM (G). B, D, F, H) Exemplary baseline corrected progress curves of EcAII-T21AzoF for c(NaCl) of 0 mM (B), 50 mM (D), 100 mM (F) and 140 mM (H). *Irradiation:* Measurements were stopped after ~25 min and the reactions were irradiated with 365 nm (2 s per 4 wells) or 420 nm (8 s per 4 wells) to establish the PSS^{365} or the PSS^{420}, respectively. *Reaction conditions:* 60 mM glutamine (saturated), 109 U/mL HRP, 100 mU/mL GOX, 3 mM 4-aminoantipyrine, 3 mM phenol, 0/50/100/140 mM NaCl, and 0.1/0.5/0.2 µM WT-EcAII/EcAII-S19AzoF/EcAII-T21AzoF in 50 mM Tris/HCl (pH 7.0/7.5/8.0/8.5) at 37°C. *Statistics:* Activity and LRF values were obtained by a linear fit of the different segments of the progress curve and are plotted as circles without the SE for two technical replicates. Mean LRF values are indicated by bars.

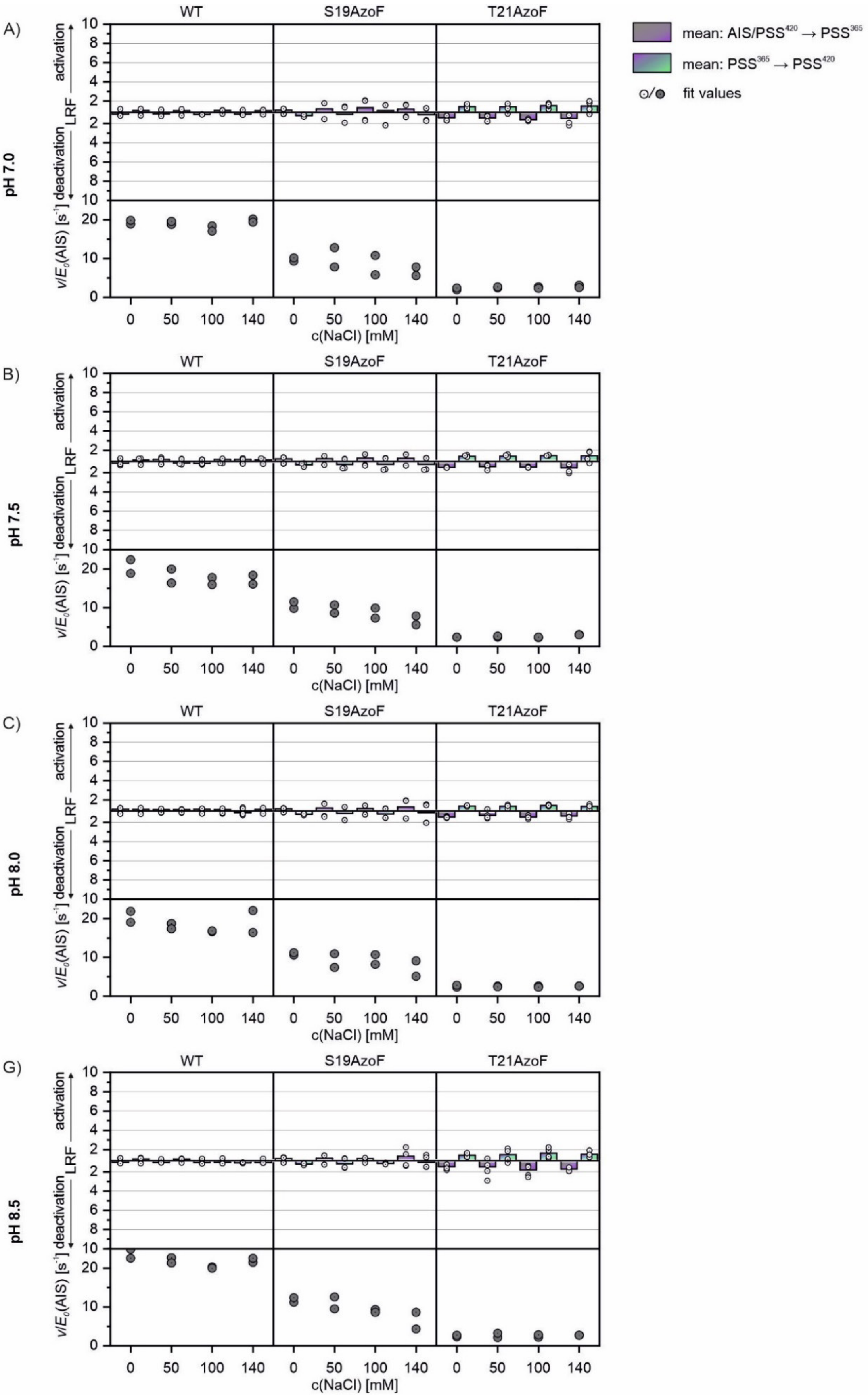

Figure S19. Asparaginase activities and LRFs at various pH for WT-EcAII, EcAII-S19AzoF and EcAII-T21AzoF determined via irradiation during the turnover measurement. Summary of the obtained LRFs (top panels) and as control the v/E_0 activity values of the as-isolated variants (bottom panels) for pH values of 7.0 (A), 7.5 (B), 8.0 (C) and 8.5 (D). For details on irradiation, reaction conditions and statistics as well as exemplary progress curves see **Figure S17**.

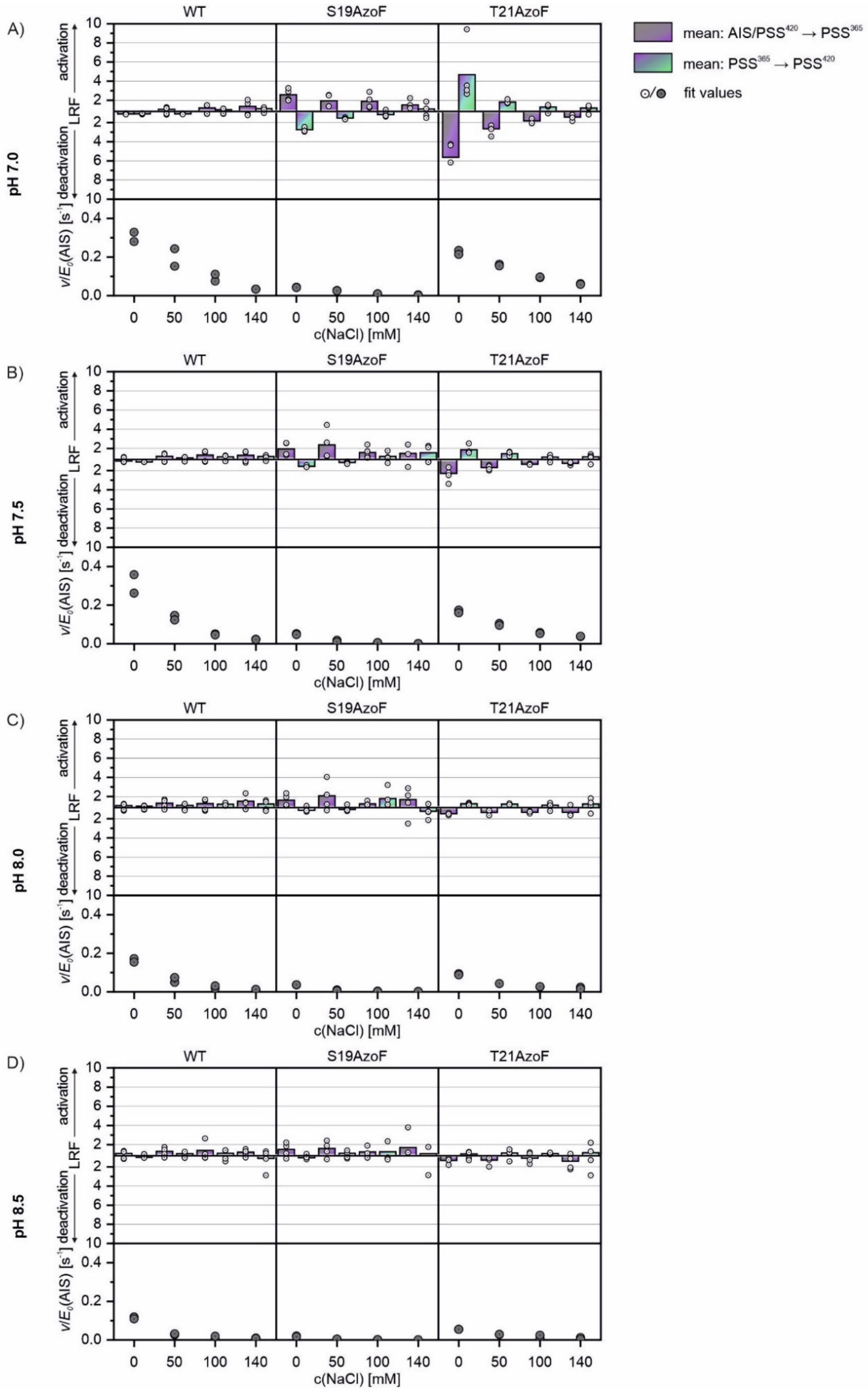

Figure S20. Glutaminase activities and LRFs at various pH for WT-EcAII, EcAII-S19AzoF and EcAII-T21AzoF determined via irradiation during the turnover measurement. Summary of the obtained LRFs (top panels) and as control the v/E_0 activity values of the as-isolated variants (bottom panels) for pH values of 7.0 (A), 7.5 (B), 8.0 (C) and 8.5 (D). For details on irradiation, reaction conditions and statistics as well as exemplary progress curves see **Figure S18**.

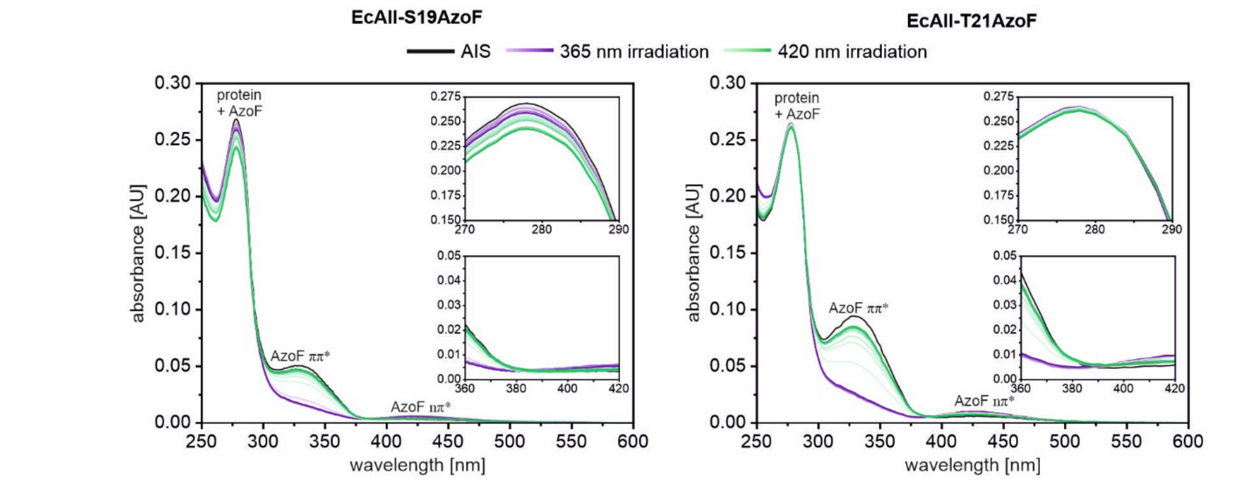

Figure S21. UV/Vis analysis of 15 µM EcAII-S19AzoF and EcAII-T21AzoF in 10 mM Tris/HCl (pH 7.0), 140mM NaCl. The AIS was stepwise irradiated with 365 nm and spectra were acquired after 1 s, 2 s, 3 s, 4 s, 5 s and 20 s total irradiation time. Then the established PSS^{365} was again stepwise irradiated with 420 nm and spectra were acquired after 1 s, 2 s, 3 s, 4 s, 5 s, 6 s, 7 s, 8 s, 20 s and 30 s total irradiation time. The insets show the regions where isosbestic points of AzoF isomerization should be. Between 270 nm and 290 nm, the UV/Vis signal decreases consistently instead of forming an isosbestic point, which indicates the presence of another reaction, e.g. the photocleavage of disulfide bonds. Between 360 nm and 420 nm, an unsharp isosbestic point is visible at ~386 nm and ~392 nm for EcAII-S19AzoF and EcAII-T21AzoF, respectively.

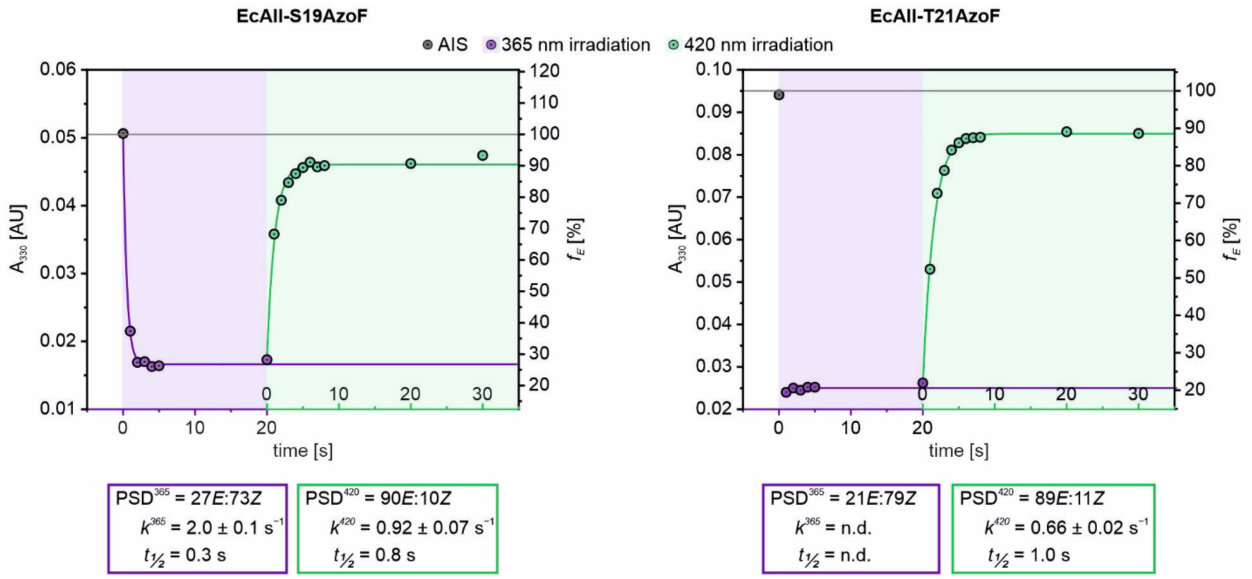

Figure S22. Progress curves following the timely change of the ππ* signal of AzoF at 330 nm from the UV/Vis analysis of EcAII-S19AzoF and EcAII-T21AzoF in 140 mM NaCl (cf. **Figure S21**). The signal decrease upon 365 nm irradiation as well as the signal increase upon 420 nm irradiation were fitted with mono-exponential equations. The plateau values of these fits were converted into f_E values using the equation obtained from the determination of *E*:*Z* distributions (cf. **Figure S10**), which define the PSD^{365} and PSD^{420}, respectively. The fit also derived the rate constants k^{365} and k^{420} (± SE) for the establishment of the PSS^{365} and the PSS^{420}, respectively, from which the half-times $t_{½}$ were calculated. Note: n.d. = not determinable.

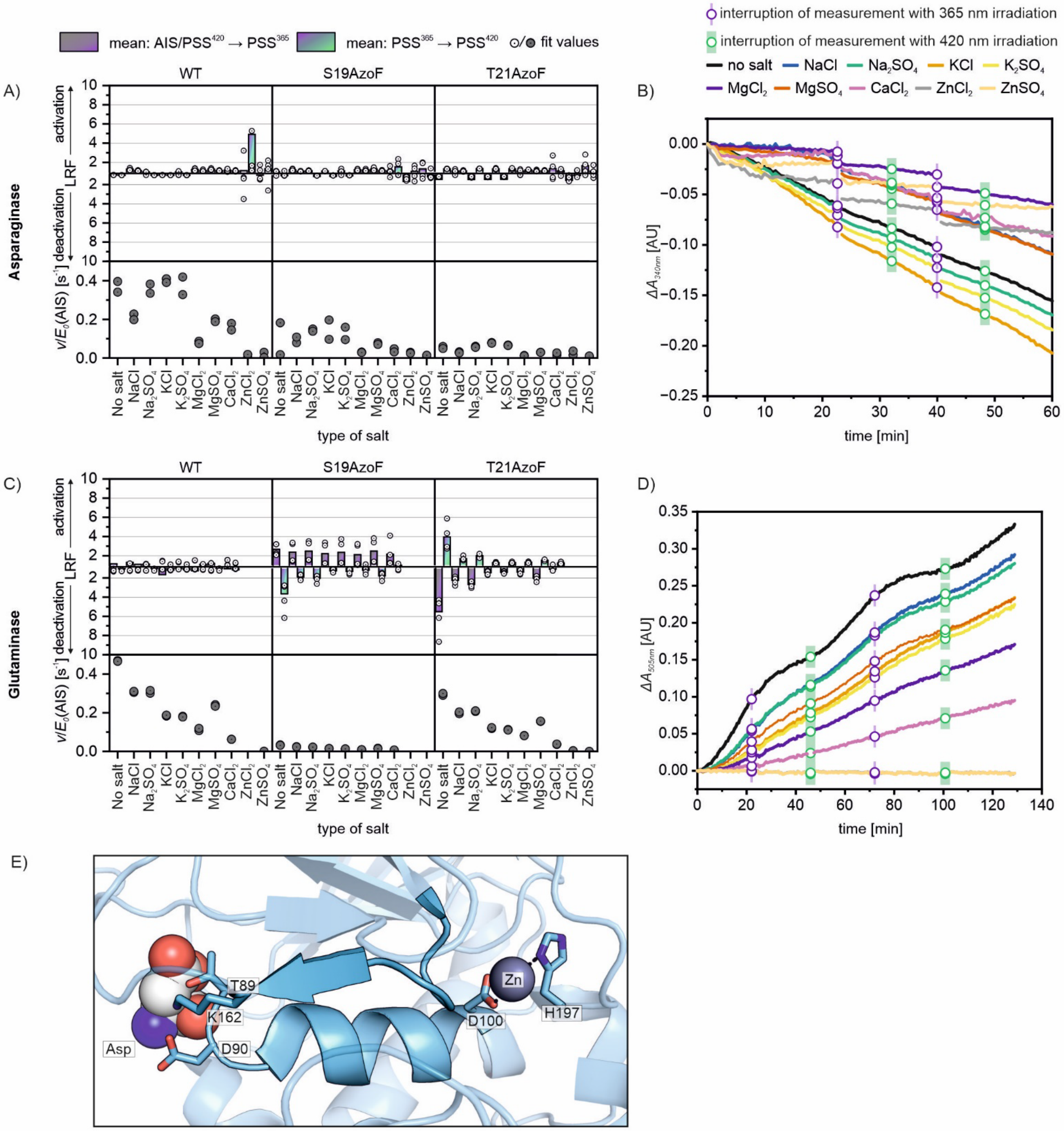

Figure S23. Asparaginase (A, B) and glutaminase (C, D) activities using various types of salt (50 mM) for WT-EcAII, EcAII-S19AzoF and EcAII-T21AzoF determined via irradiation during the turnover measurement. A, C) Summary of the obtained LRFs (top panels) and as control the v/E_0 activity values of the as-isolated variants (bottom panels). Note: In some cases, enzyme activity in the presence of Zn^{2+} was too low to be determined. B, D) Exemplary baseline corrected progress curves of EcAII-T21AzoF. *Irradiation:* Measurements were stopped after ~25 min and reactions were irradiated for various time periods with 365 nm (2 s per 4 wells) or 420 nm (8 s per 4 wells) to establish the PSS^{365} or the PSS^{420}, respectively. *Reaction conditions (asparaginase):* 6 mM asparagine (saturated), 14 U/mL GDH, 0.25 mM NADH, 5 mM α-ketoglutarate, 0/50 mM salt, and 2/3/5 nM WT-EcAII/EcAII-S19AzoF/EcAII-T21AzoF in 50 mM Tris/HCl (pH 7.0) at 37°C. *Reaction conditions (glutaminase):* 60 mM glutamine (saturated), 109 U/mL HRP, 100 mU/mL GOX, 3 mM 4-aminoantipyrine, 3 mM phenol, 0/50 mM salt, and 0.1/0.5/0.3 µM WT-EcAII/EcAII-S19AzoF/EcAII-T21AzoF in 50 mM Tris/HCl (pH 7.0) at 37°C. *Statistics:* Activity and LRF values were obtained by a linear fit of the different segments of the progress

curve and are plotted as circles without the SE for two technical replicates. Mean LRF values are indicated by bars. E) EcAII harbors a metal binding site marked by a Zn^{2+} coordinated in a tetrahedral fashion by D100 and H197 and two water molecules positioned by D156, which is directly connected to the catalytic residues T89 and D90 via an alpha helix, and K162 via a beta sheet. Apo WT-EcAII (PDB-ID: 6eok) was superposed with aspartate (PDB-ID: 3eca).

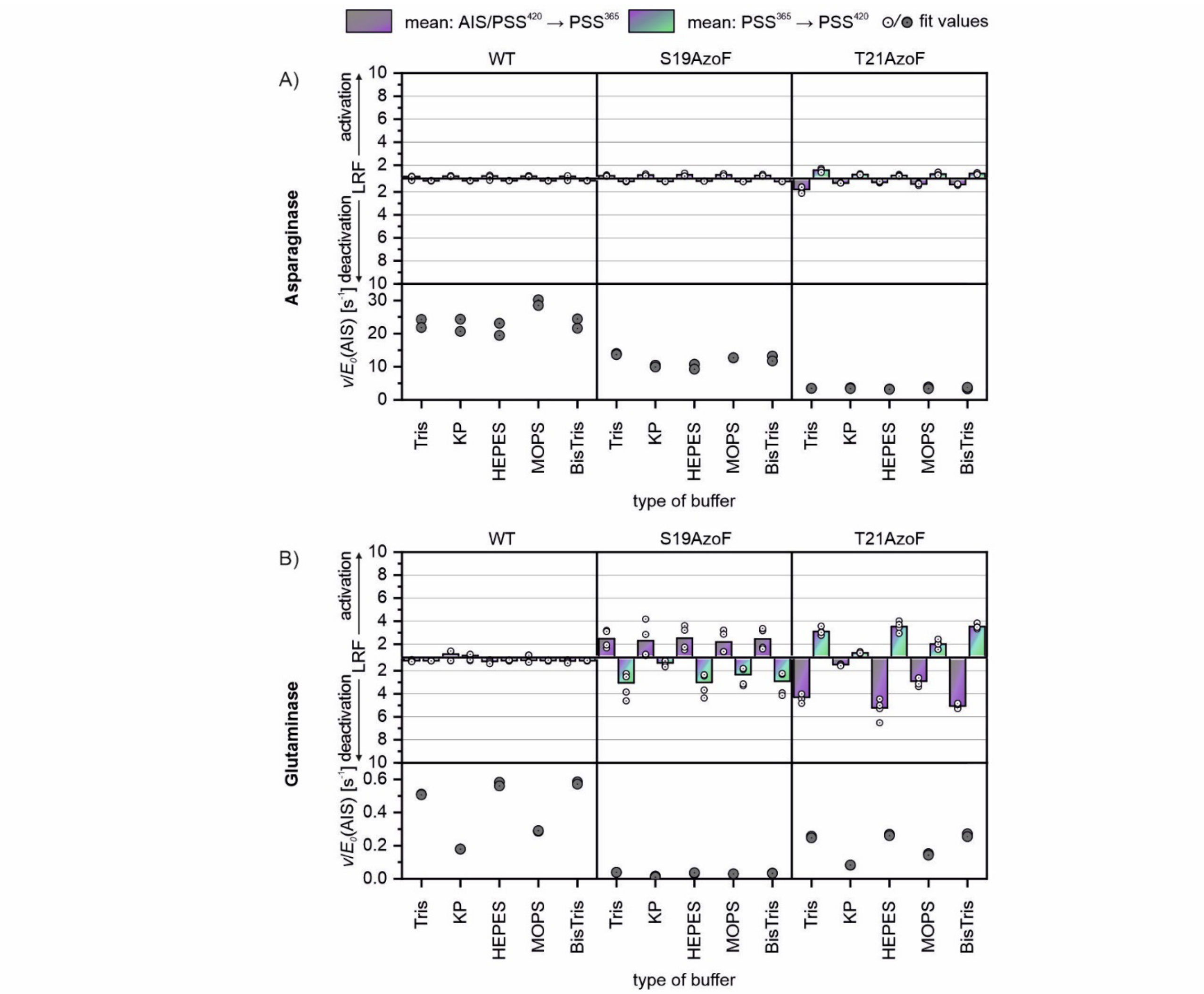

Figure S24. Asparaginase (A) and glutaminase (B) activities using various types of buffer for WT-EcAII, EcAII-S19AzoF and EcAII-T21AzoF determined via irradiation during the turnover measurement. Shown is a summary of the obtained LRFs (top panels) and as control the v/E_0 activity values of the as-isolated variants (bottom panels). *Irradiation:* Measurements were stopped after ~10 min (ASNase) and ~25 min (GLNase) and reactions were irradiated for various time periods with 365 nm (2 s per 4 wells) or 420 nm (8 s per 4 wells) to establish the PSS^{365} or the PSS^{420}, respectively. *Reaction conditions (asparaginase):* 6 mM asparagine (saturated), 14 U/mL GDH, 0.25 mM NADH, 5 mM α-ketoglutarate, and 2/3/4 nM WT-EcAII/EcAII-S19AzoF/EcAII-T21AzoF in 50 mM buffer (pH 7.0) at 37°C. *Reaction conditions (glutaminase):* 60 mM glutamine (saturated), 109 U/mL HRP, 100 mU/mL GOX, 3 mM 4-aminoantipyrine, 3 mM phenol, and 0.1/0.5/0.2 µM WT-EcAII/EcAII-S19AzoF/EcAII-T21AzoF in 50 mM buffer (pH 7.0) at 37°C. *Statistics:* Activity and LRF values were obtained by a linear fit of the different segments of the progress curve and are plotted as circles without the SE for two technical replicates. Mean LRF values are indicated by bars.

Evaluation of the reversibility of photocontrol

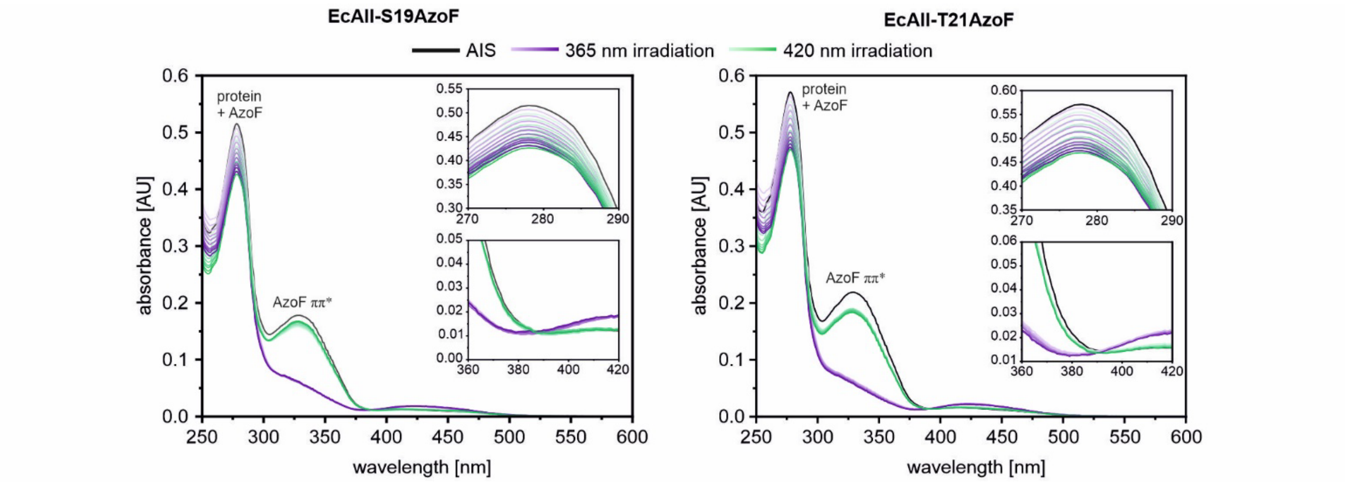

Figure S25. UV/Vis analysis of 20 µM EcAII-S19AzoF and EcAII-T21AzoF (in 50 mM Tris/HCl pH 7.0, 50 mM NaCl). The AIS was repeatedly irradiated with 365 nm (2 s) and 420 nm (8 s). After each step, spectra were acquired. The insets show the regions where isosbestic points of AzoF isomerization should be. Between 270 nm and 290 nm, the UV/Vis signal decreases consistently instead of forming an isosbestic point, which indicates the presence of another reaction, e.g. the photocleavage of disulfide bonds. Between 360 nm and 420 nm, an isosbestic point is visible at ~388 nm and ~391 nm for EcAII-S19AzoF and EcAII-T21AzoF, respectively. *E*:*Z* distributions were determined as described in **Figure S10**.

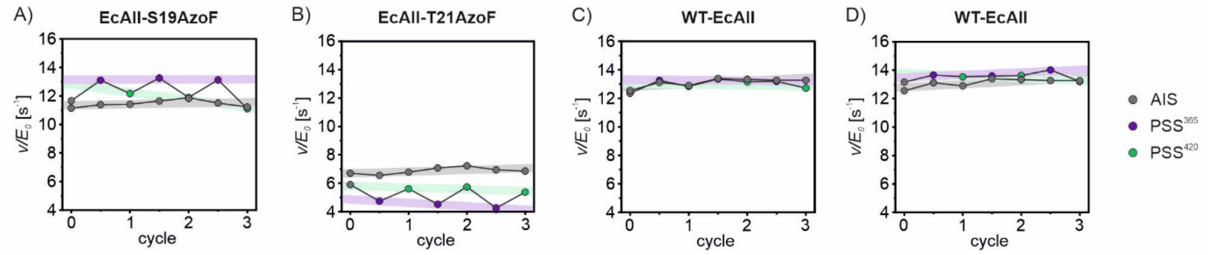

Figure S26. Cycle performance of asparaginase photocontrol for EcAII-S19AzoF (A), EcAII-T21AzoF (B) and WT-EcAII (C,D). Shown are the results of the first (C) and second (A, B, D) replicate. Repeated irradiation during the turnover measurement demonstrates good reversibility of the minimal photocontrol in the two photoxenases and no photocontrol in WT-EcAII. *Irradiation:* Measurements were stopped after ~10 min and reactions were irradiated with 365 nm (2 s per 4 wells) or 420 nm (8 s per 4 wells) to establish the PSS^{365} or the PSS^{420}, respectively. *Reaction conditions:* 5 mM asparagine (saturated), 14 U/mL GDH, 0.25 mM NADH, 5 mM α-ketoglutarate, and 3 nM EcAII-S19AzoF, 4 nM EcAII-T21AzoF, and 2 nM WT-EcAII in 10 mM Tris/HCl (pH 7.0) at 37°C. *Statistics:* Measurements were performed in technical replicates.

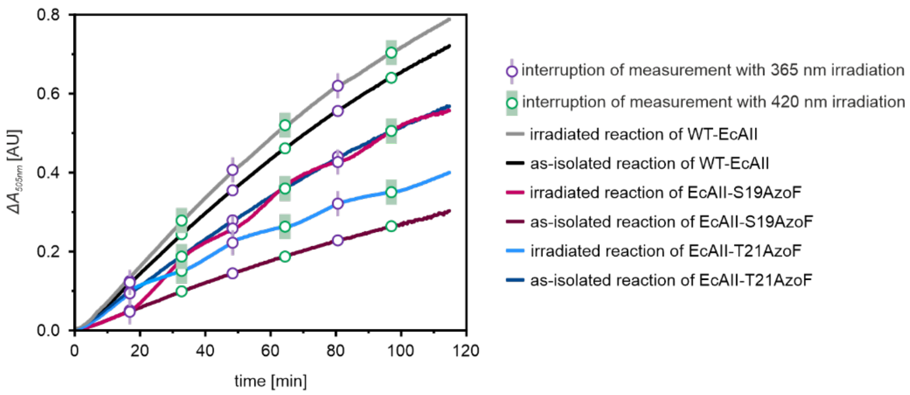

Figure S27. Cycle performance of glutaminase photocontrol for EcAII-S19AzoF, EcAII-T21AzoF and WT-EcAII as baseline corrected progress curves (exemplarily shown for the second replicate). Repeated irradiation during the turnover measurement demonstrates good reversibility of the photocontrol in the two irradiated photoxenases and no photocontrol in WT-EcAII as well as in the AIS reactions of the photoxenases. *Irradiation:* Measurements were stopped after ~15 min and reactions were irradiated with 365 nm (2 s per 4 wells) or 420 nm (8 s per 4 wells) to establish the PSS^{365} or the PSS^{420}, respectively. *Reaction conditions:* 60 mM glutamine (saturated), 540 U/mL HRP, 300 mU/mL GOX, 3 mM 4-aminoantipyrine, 3 mM phenol, and 0.1/0.5/0.3 µM WT-EcAII/EcAII-S19AzoF/EcAII-T21AzoF in 10 mM Tris/HCl (pH 7.0) at 37°C.

Extended Text S3. Adjustment of the coupled glutaminase assay

In coupled enzymatic assays, a lag phase describes the time needed for all substrates and enzymes to reach the steady state and is highly dependent on the v_{max} of the auxiliary enzymes. To determine the lag phase of our coupled enzymatic assay in various reaction conditions, we therefore recorded the activity v_{max} (in µM s^{-1}) of the limiting auxiliary enzyme GOX (100 mU/mL) coupled to HRP (109 U/mL) using saturated concentrations of glutamate concentrations (15 mM), 4-aminoantipyrin (3 mM) and 3 mM phenol, and in the presence of various concentrations of NaCl in 10 mM Tris/HCl (various pH) at 37 °C. Literature provided a K_m value of ~0.2 mM for GOX from *Streptomyces sp.*.[6] Since we aimed to approximate the lag phase for various reaction conditions, in which the K_m value might change, we used 1 mM for a rough estimation of the lag time τ (= K_m/v_{max}). With this we determined the time the coupled GOX/HRP assay requires to reach 90% ($t_{90\%ss}$ = 2.29τ) or 99% ($t_{99\%ss}$ = 4.64τ) of the stationary state (**Table S5**).[4,7] For most reaction conditions $t_{90\%ss}$ lay below 2 min and $t_{99\%ss}$ below 4 min. Only at pH 7.0 the $t_{90\%ss}$ increased up to 19 min and the $t_{99\%ss}$ up to 37 min. This explained the relatively long lag phase in photocontrol measurements at pH 7.0, in which we run the reaction for at least ~25 min after irradiation achieving 90% of the stationary state (e.g. **Figure S18B**, black). While 90% stationary state is adequate for a large screen of activities, we decided to increase the GOX concentration to 300 mU/mL decreasing $t_{99\%ss}$ threefold to ~12 min for the examination of reversibility. As a result, the lag phase disappeared and the transitions of the progress curve after each irradiation step were sharper (**Figure S27**, light-blue and pink)

Table S5. Fitted activity values *v* of GOX coupled to HRP and estimated lag times *t* until 90% or 99% of the stationary state (ss) in the GOX/HRP coupled enzymatic assay of EcAII should be reached.

pH	c(NaCl)	*v* [µM s^{-1}]	$t_{90\%ss}$ [min]	$t_{99\%ss}$ [min]
7.0	0 mM	2.7	14	29
7.5		28.5	1	3
8.0		37.1	1	2
8.5		29.9	1	3
7.0	50 mM	2.6	15	29
7.5		28.2	1	3
8.0		37.3	1	2
8.5		27.4	1	3
7.0	100 mM	2.1	19	37
7.5		29.8	1	3
8.0		34.0	1	2
8.5		20.8	2	4
7.0	140 mM	2.1	18	37
7.5		27.2	1	3
8.0		34.6	1	2

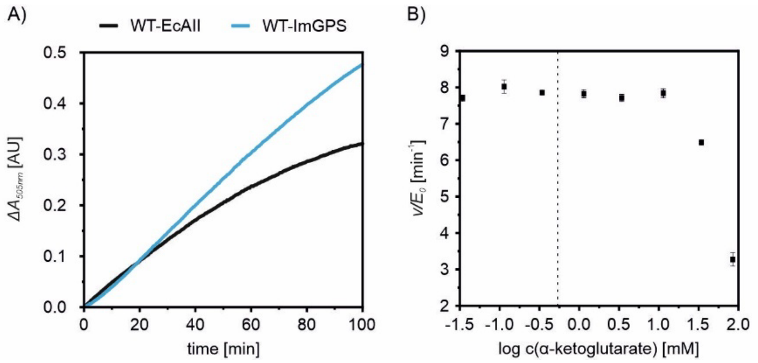

Figure S28. Evaluation of the exponential course of the EcAII glutaminase progress curve. A) Comparison of the glutaminase reaction progress in substrate saturation of WT-EcAII and WT-ImGPS using enzyme concentrations that achieve similar activities. While the WT-EcAII reaction follows an exponential course, the WT-ImGPS reaction is quasi-linear as expected for reactions in substrate saturation. *Reaction conditions*: 60 mM glutamine, 300 mU/mL GOX, 540 U/mL HRP, 3 mM 4-aminoantipyrine, 3 mM phenol, and 70 µM ProFAR (in the ImGPS reaction) and 0.1 µM EcAII (10-fold dilution) or 0.13 µM ImGPS (10-fold dilution) in 10 mM Tris/HCl (pH 7.0) at 37°C. B) Glutaminase activities v/E_0 of WT-EcAII in the presence of varying concentrations of α-ketoglutarate, one of the final products of the coupled GOX/HRP assay. Inhibition occurs at concentrations significantly higher than those produced during the reaction course (dashed line; equivalent to the produced quinoneimine concentrations; cf. **Figure S5A**). *Reaction conditions*: 1.14 mM glutamine (near K_m), 100 mU/mL GOX, 120 U/mL HRP, 3 mM 4-aminoantipyrine, 3 mM phenol, 0.0342-85 mM α-ketoglutarate, and 0.2 µM EcAII (10-fold dilution) in 70 mM Tris/HCl (pH 7.0) at 37°C. The protein was incubated with the corresponding amount of α-ketoglutarate on ice for one hour prior the addition to the assay.

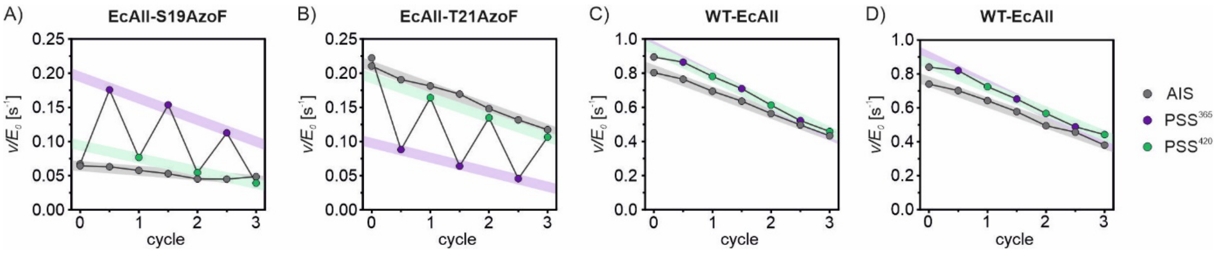

Figure S29. Cycle performance of glutaminase photocontrol for EcAII-S19AzoF (A), EcAII-T21AzoF (B) and WT-EcAII (C). Shown are the results of the first (C, left) and second (A, B, C, right) replicate. Repeated irradiation during the turnover measurement demonstrates good reversibility of the photocontrol in the two photoxenases and no photocontrol in WT-EcAII. For irradiation and reaction conditions see **Figure S27**. *Statistics:* Measurements were performed in a technical replicate for EcAII-S19AzoF and WT-EcAII, and in a biological replicate for EcAII-T21AzoF.

Correlation of photocontrol efficiency with conformational traits of EcAII

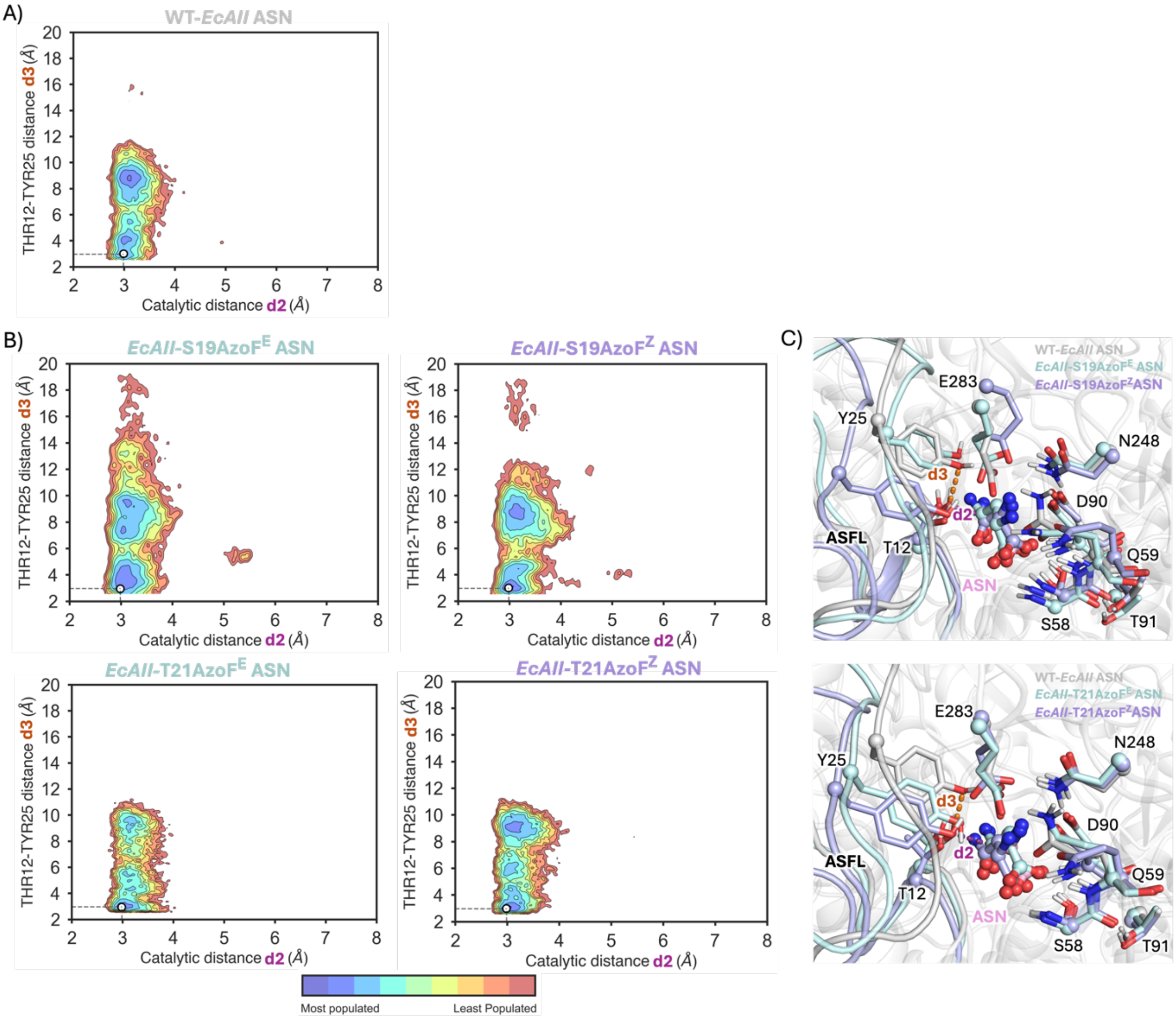

Figure S30. Reconstructed conformational landscapes for asparagine-bound WT-EcAII (A), and EcAII-photoxenases (B,C). A, B) Conformational landscapes derived from MD simulations using distances d2 and d3. As a reference, the position of the minima found in the case of WT-EcAII in presence of asparagine is marked using a white dot. C) Overlay of a representative structure extracted from the most populated minimum for EcAII-S19/T21AzoF^E (cyan), EcAII-S19/T21AzoF^Z (purple) and WT-EcAII (grey) as reference. Asparagine is shown in a ball-and-stick representation.

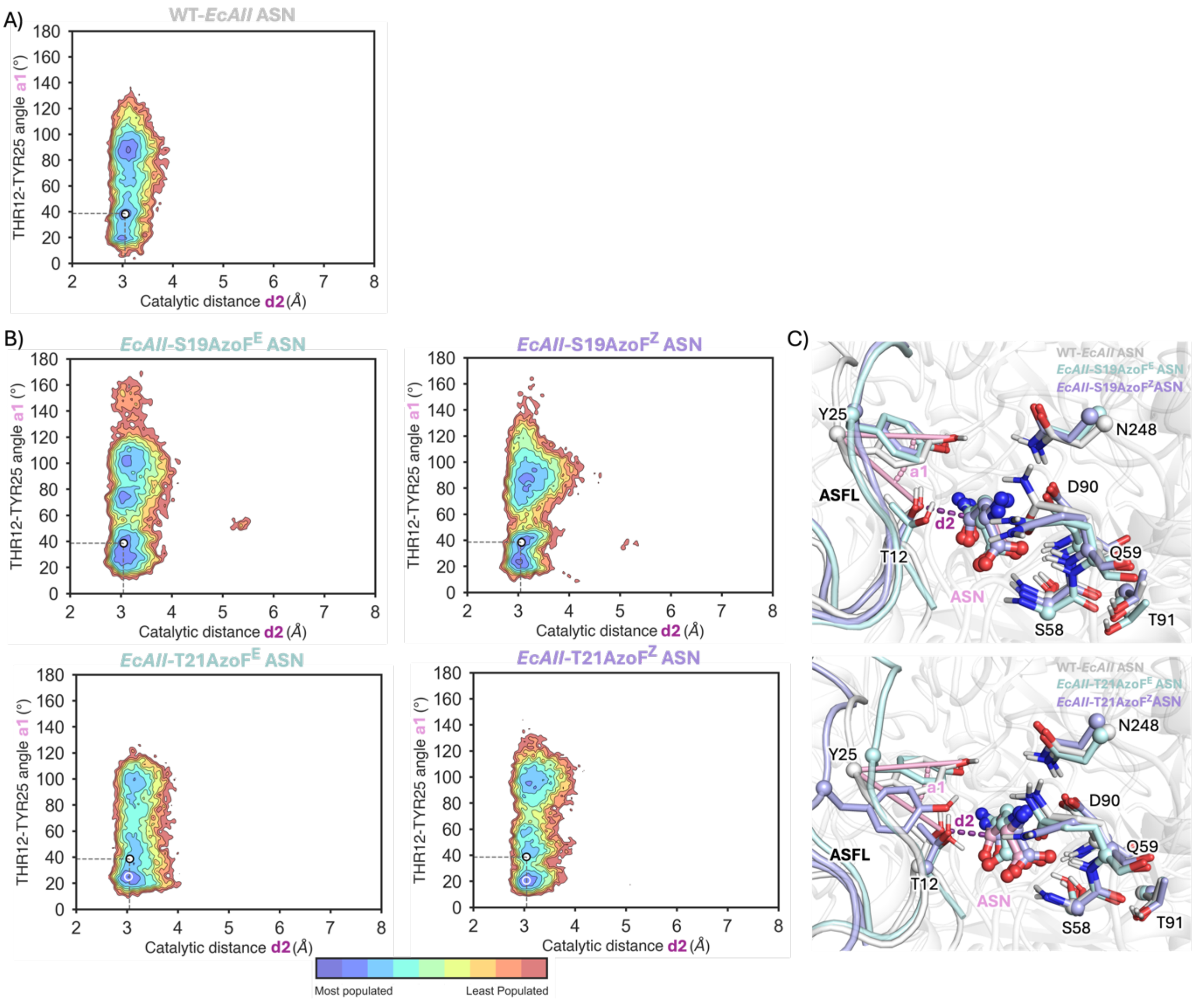

Figure S31. Reconstructed conformational landscapes for asparagine-bound WT-EcAII (A), and EcAII-photoxenases (A,C). A,B) Conformational landscapes derived from MD simulations using distances d2 and a1. As a reference, the position of the minima found in the case of WT-EcAII in presence of asparagine is marked using a white dot. C) Overlay of a representative structure extracted from the most populated minimum, marked with a white non-filled circle in the conformational landscape, for EcAII-S19/T21AzoF^{E} (cyan), EcAII-S19/T21AzoF^{Z} (purple) and WT-EcAII (grey) as reference. Glutamine is shown in a ball-and-stick representation.

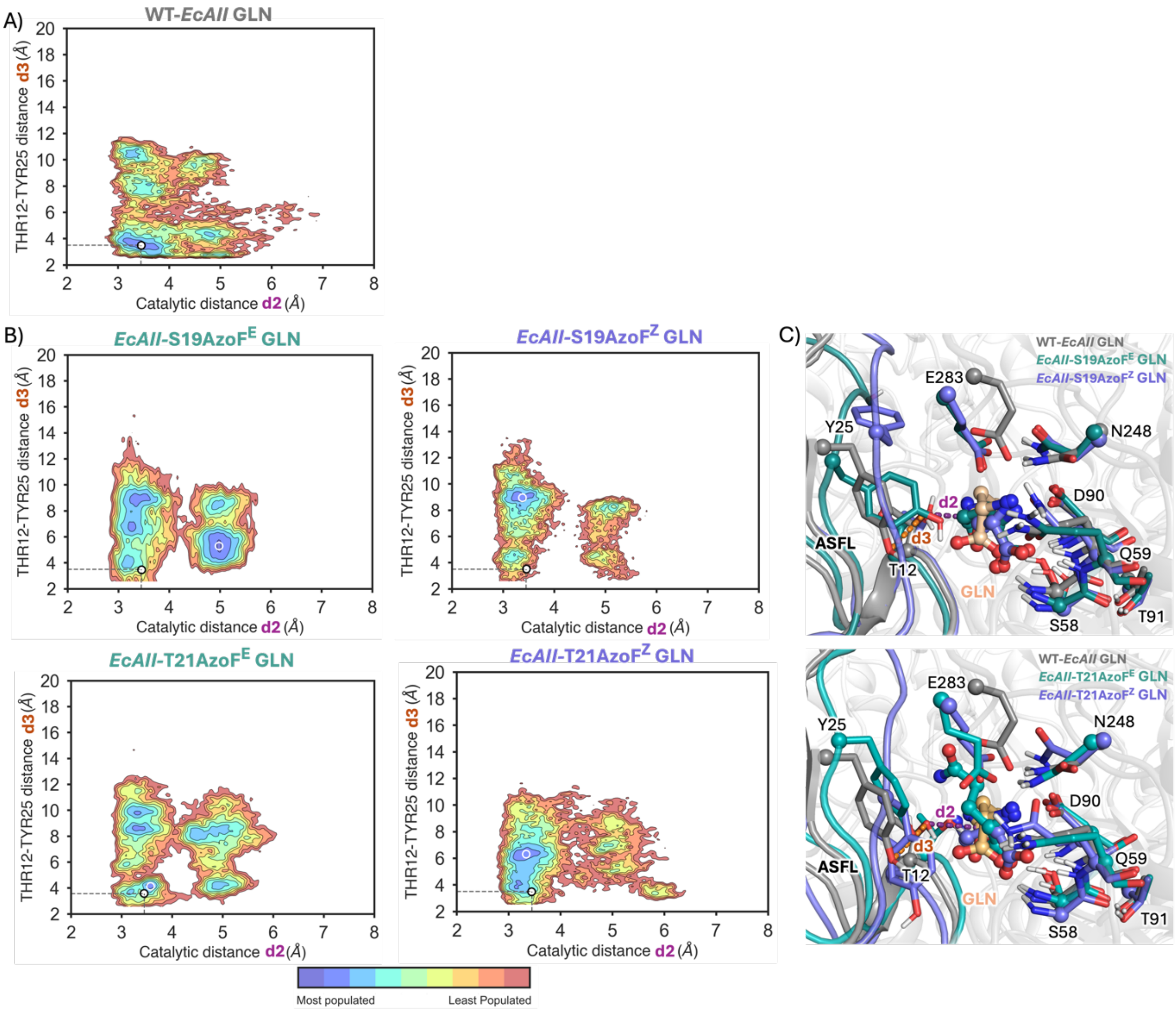

Figure S32. Reconstructed conformational landscapes for glutamine-bound WT-EcAII (A), and EcAII-photoxenases (B,C). A,B) Conformational landscapes derived from MD simulations using distances d2 and d3. As a reference, the position of the minima found in the case of WT-EcAII in presence of glutamine is marked using a white dot. C) Overlay of a representative structure extracted from the most populated minimum, marked with a white non-filled circle in the conformational landscape, for EcAII-S19/T21AzoF^{E} (cyan), EcAII-S19/T21AzoF^{Z} (purple) and WT-EcAII (grey) as reference. Glutamine is shown in a ball-and-stick representation.

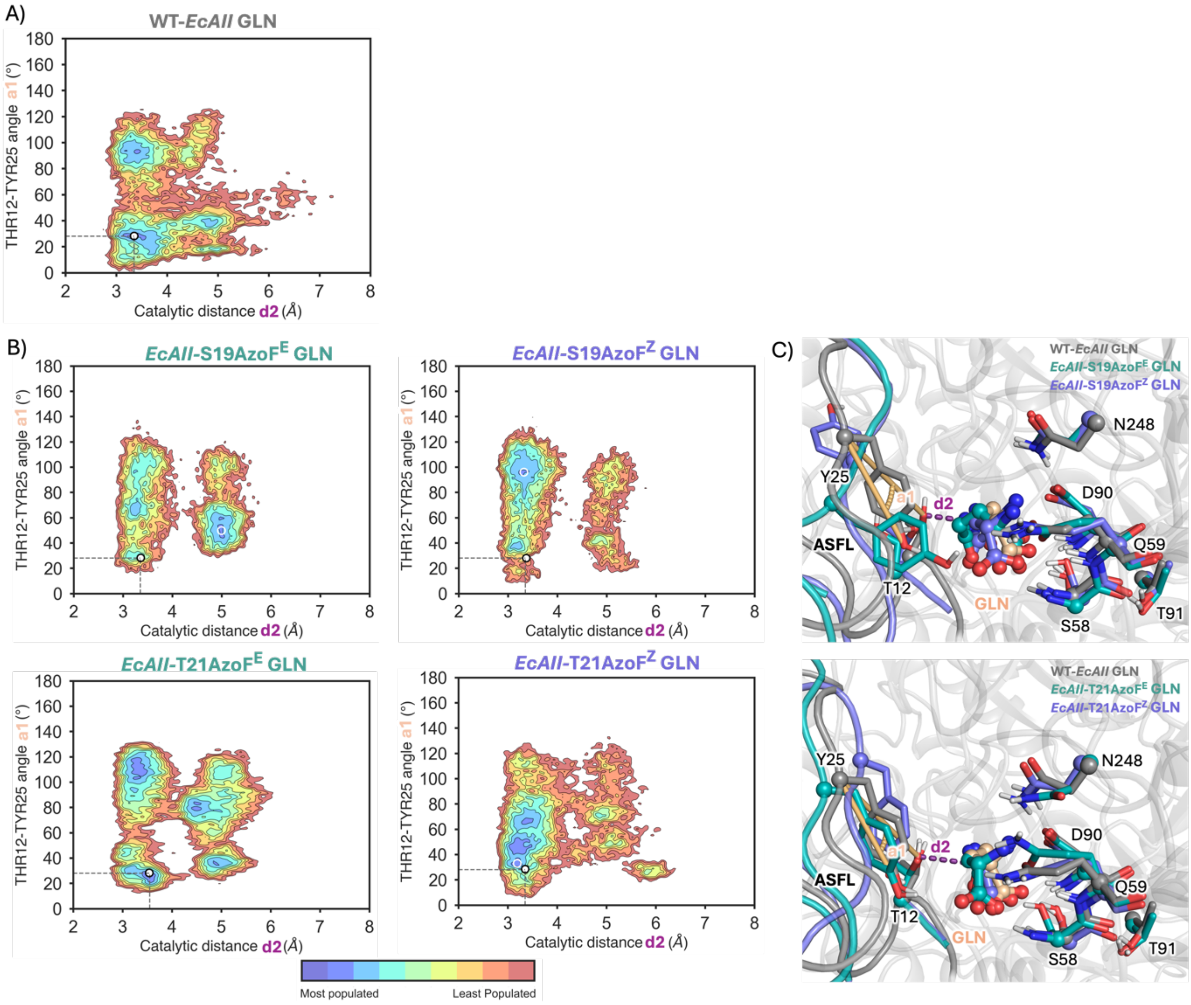

Figure S33. Reconstructed conformational landscapes for glutamine-bound WT-EcAII (A), and EcAII-photoxenases (B,C). A,B) Conformational landscapes derived from MD simulations using distances d2 and a1. As a reference, the position of the minima found in the case of WT-EcAII in presence of glutamine is marked using a white dot. C) Overlay of a representative structure extracted from the most populated minimum, marked with a white non-filled circle in the conformational landscape, for EcAII-S19/T21AzoF^E (cyan), EcAII-S19/T21AzoF^Z (purple) and WT-EcAII (grey) as reference. Glutamine is shown in a ball-and-stick representation.

Extended Text S4. MD simulations explain the reduced catalytic activity of EcAII-photoxenases.

We evaluated our MD simulations regarding a possible explanation for the decreased asparaginase k_{cat} of EcAII-T21AzoF as well as reduced glutaminase k_{cat} of EcAII-S19AzoF. Notably, while EcAII-S19AzoF$^{E/Z}$ demonstrates a similar closure and conformation of the ASFL as well as the same productive binding of asparagine compared to WT-EcAII, EcAII-T21AzoF$^{E/Z}$ shows deviations particularly in the ASFL conformation as determined in the overlay of representative structures from the most populated minima (**Figure 6B**). Together with a less optimal conformation of Y25 for T12 deprotonation (a1) compared to the most populated WT-EcAII structure (**Figure 31C**), this likely explains the two-fold reduced k_{cat} of EcAII-T21AzoF compared to WT-EcAII (cf. **Figure 2**). Furthermore, the overlay of a representative structure of the most populated minima for glutamine-bound EcAII-S19AzoF and EcAII-T21AzoF showed large deviations in terms of ASFL conformation in comparison to WT-EcAII (**Figure 6D**), especially in the case of EcAII-S19AzoF, which explains its substantially lower kinetic values as compared to WT-EcAII. In this regard, we were particularly interested in the comparison of EcAII-S19AzoF, which exhibited a 12-fold reduced k_{cat} and a 21-fold reduced k_{cat}/K_m compared with WT-EcAII in the presence of glutamine. To elucidate the reasons for this major deactivation we focused on the comparison of the catalytically relevant nucleophilic distances d2 and d3 (**Figure S34**) and the Y25-T12 angle a1 (**Figure S33**). The presence of the bulkier glutamine in EcAII-S19AzoF favors a different conformation of ASFL, which in turn affects the positioning of Y25 that is displaced out of the active site (d2 and d3 > 3.5 Å, (**Figure S34A,B; Figure S31B**). The overlay of the most stable conformations of EcAII-S19AzoF in the presence of either asparagine or glutamine (**Figure S34C,D**) and comparison with the ones of WT-EcAII shows no major differences in terms of asparagine or glutamine binding in the active site pocket, however, substantial deviations in ASFL and Y25 conformation are observed especially in the case of EcAII-S19AzoF (**Figure S33C**). We further found that AzoF incorporated at position 19 can establish hydrophobic interactions with the adjacent alpha$_{32\text{-}46}$-helix from the other subunit in the presence of glutamine (**Figure S35**). Such interaction is absent when the smaller asparagine is bound in the active site and favors an alternative catalytically less favorable conformation of the ASFL.

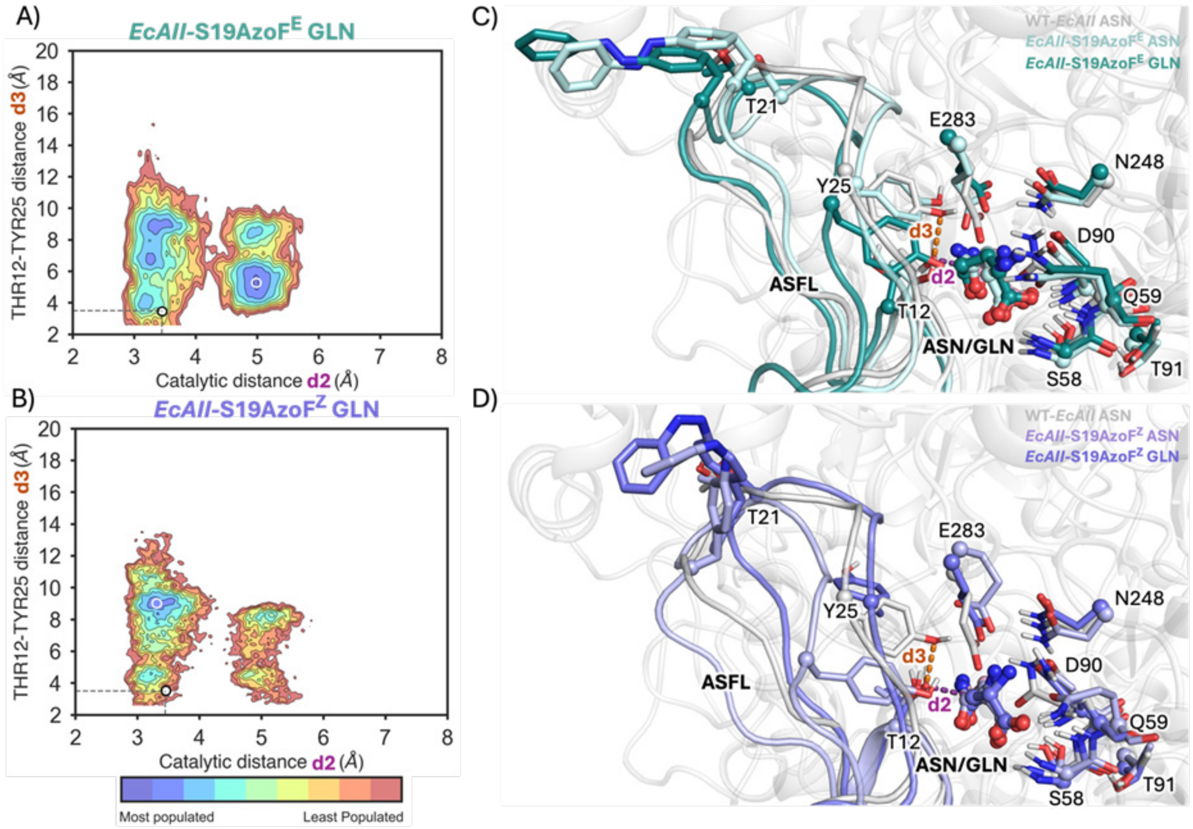

Figure S34. Reconstructed conformational landscapes for glutamine-bound EcAII-S19AzoF^{E} (A), EcAII-S19AzoF^{Z} (B) focusing on the catalytically relevant d2 and d3 distances (in Å). As a reference, the position of the minima found in the case of WT-EcAII in presence of glutamine is marked using a white dot. C, D) Comparison of representative structures extracted from the most populated minima, marked using a white non-filled circle in the conformational landscapes A-B, for EcAII-S19AzoF^{Z} (purple) and EcAII-S19AzoF^{E} (cyan) in the presence of either asparagine (structure shown in lighter color) and glutamine (brighter color). In all cases a representative structure of WT-EcAII in the presence of asparagine is also provided (shown in grey).

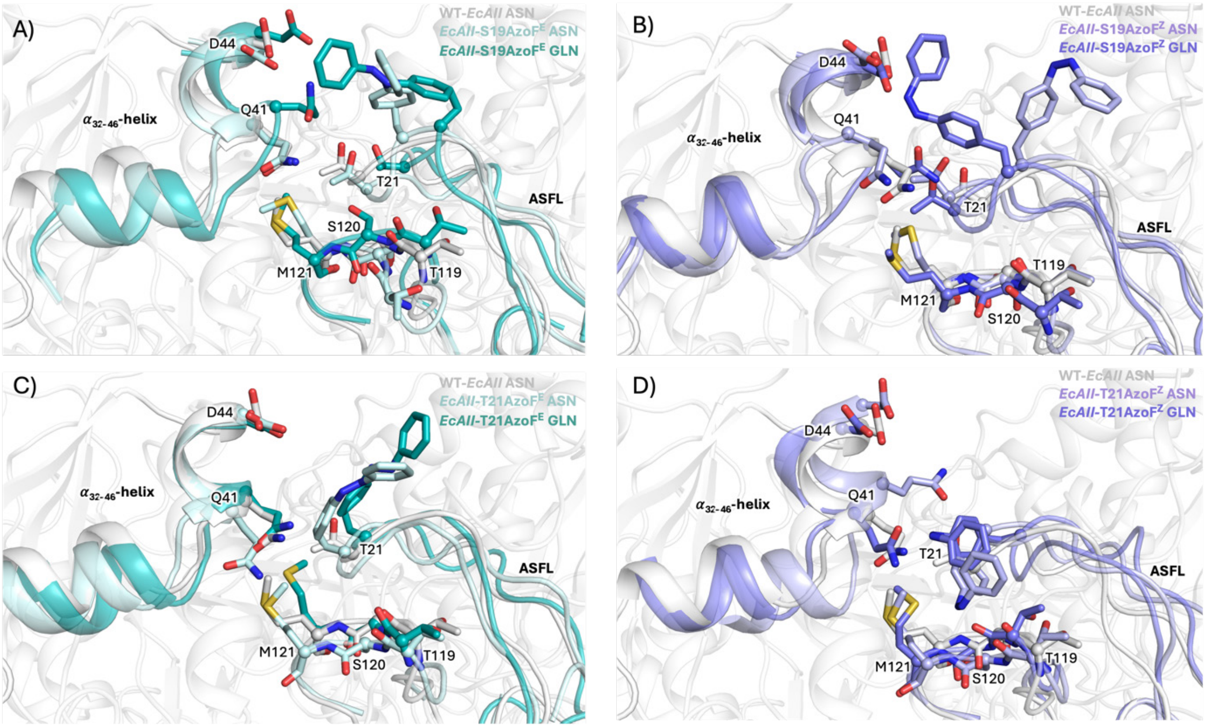

Figure S35. Overlay of a representative structure extracted from the most populated minimum (cf. **Figure 5**, **Figure 6**) for EcAII-S19AzoF^E (A), EcAII-S19AzoF^Z (B) EcAII-T21AzoF^E (C), EcAII-T21AzoF^Z (D) in the presence of either asparagine (structure shown in lighter color) and glutamine (brighter color). WT-EcAII in the presence of asparagine is also provided as a reference (in grey).

SUPPLEMENTARY MATERIAL FOR THE DISCUSSION

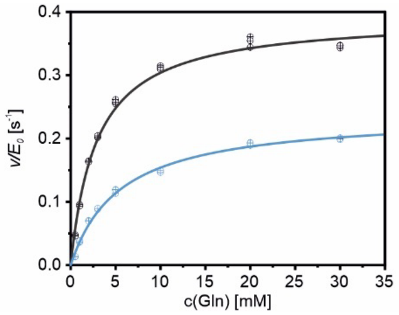

	k_{cat} [s^{-1}]	K_m [mM]	k_{cat}/K_m [10^3 s^{-1} M^{-1}]	R^2
**0 mM NaCl**	0.39 ± 0.01	2.92 ± 0.22	0.134 ± 0.006	0.99
**140 mM NaCl**	0.24 ± 0.02	5.51 ± 0.75	0.043 ± 0.004	0.98
**allosteric inactivation**	1.6x	1.9x	3.1x	

Figure S36. Michaelis-Menten curves, fitting values and statistics for the glutaminase activity of EcAII-T21AzoF in the presence and absence of NaCl and at physiological pH. Steady-state kinetics were measured in the AIS. *Reaction conditions:* 0.5–30 mM glutamine, 120 mU/mL GOX, 300 U/mL HRP, 3 mM 4-aminoantipyrine, 3 mM phenol, and 0 mM (dark grey) or 140 mM (blue) NaCl and 0.3 µM EcAII (10-fold dilution) in 10 mM Tris/HCl (pH 7.4) at 37°C. *Statistics:* Each circled value represents the fitted initial catalytic rate v/E_0; three technical replicates were measured for each substrate concentration. k_{cat}, k_{cat}/K_m *and* K_m values are given as fitting value ± SE; R^2 represents the corrected R^2 as determined in Origin 2024.

References

(1) Lubkowski, J.; Vanegas, J.; Chan, W.-K.; Lorenzi, P. L.; Weinstein, J. N.; Sukharev, S.; Fushman, D.; Rempe, S.; Anishkin, A.; Wlodawer, A. Mechanism of catalysis by L-asparaginase. *Biochemistry* **2020**, *59* (20), 1927–1945. DOI: 10.1021/acs.biochem.0c00116.

(2) Schwinn, K.; Ferré, N.; Huix-Rotllant, M. UV-visible absorption spectrum of FAD and its reduced forms embedded in a cryptochrome protein. *Phys. Chem. Chem. Phys.* **2020**, *22* (22), 12447–12455. DOI: 10.1039/D0CP01714K.

(3) Kincaid, J. R.; Zheng, Y.; Al-Mustafa, J.; Czarnecki, K. Resonance Raman spectra of native and mesoheme-reconstituted horseradish peroxidase and their catalytic intermediates. *J. Biol. Chem.* **1996**, *271* (46), 28805–28811. DOI: 10.1074/jbc.271.46.28805.

(4) Copeland, R. A. Chemical Bonds and Reactions in Biochemistry. In *Enzymes:A practical introduction to structure, mechanism, and data analysis,* 3rd ed.; Copeland, R. A., Ed.; John Wiley & Sons, Inc., 2023; pp 11–38. DOI: 10.1002/9781119793304.ch2.

(5) Hiefinger, C.; Mandl, S.; Wieland, M.; Kneuttinger, A. Chapter Eight:Rational design, production and in vitro analysis of photoxenoproteins. In *Integrated Methods in Protein Biochemistry: Part C*; Shukla, A. K., Ed.; Methods in Enzymology; Academic Press, 2023; pp 247–288. DOI: 10.1016/bs.mie.2022.12.003.

(6) Utsumi, T.; Arima, J.; Sakaguchi, C.; Tamura, T.; Sasaki, C.; Kusakabe, H.; Sugio, S.; Inagaki, K. Arg305 of *Streptomyces* l-glutamate oxidase plays a crucial role for substrate recognition. *Biochem. Biophys. Res. Commun.* **2012**, *417* (3), 951–955. DOI: 10.1016/j.bbrc.2011.12.033.

(7) Copeland, R. A. Experimental measures of steady-state enzyme activity. In *Enzymes:A practical introduction to structure, mechanism, and data analysis,* 3rd ed.; Copeland, R. A., Ed.; John Wiley & Sons, Inc., 2023; pp 193–260. DOI: 10.1002/9781119793304.ch7.
